# Supplementary material for: When Doctor Means Teacher: An Interactive Workshop on Patient-Centered Education
Source: MedEdPORTAL. 2020 Dec 10;16:11053. doi: 10.15766/mep_2374-8265.11053 (PMC7732137; doi:10.15766/mep_2374-8265.11053)
Supplement: Supplementary file 1 — Facilitator Guide.docxPresurvey.docxSession 1 Patient Education Diagnoses.pptxVideo.mp4Session 1 Role-Play Scenarios.docxSession 1 Postsurvey.docxMedication Research Worksheet.docxSession 2 Patient Education Medications.pptxSession 2 Role-Play Scenarios.docxSession 2 Postsurvey.docx [file mep_2374-8265.11053-s001.zip › A. Facilitator Guide.docx]

**When Doctor Means Teacher: Facilitator Guide**

**Total Workshop Time:** 1.5 hours x 2 sessions

**Intended Audience:** Medical students on clinical clerkships (though can be modified for other learners)

**Intended Number of Participants:** Variable (original group of 20-30 participants, though can be scaled to any size group; role-play scenarios written for groups of 3)

**Educational Objectives:**

By the end of this activity, learners will be able to:

1. Explain the importance of providing patient-centered education for diagnoses and medications
2. Describe five key elements of patient-centered education diagnoses and medications
3. Demonstrate confidence in patient-centered education diagnoses and medications

**Appendices:**

1. Facilitator Guide.docx
2. Pre-Survey.docx
3. Session 1 Patient Education Diagnoses.pptx
4. Video.mp4
5. Session 1 Role-Play Scenarios.docx
6. Session 1 Post-Survey.docx
7. Medication Research Worksheet.docx
8. Session 2 Patient Education Medications.pptx
9. Session 2 Role-Play Scenarios.docx
10. Session 2 Post-Survey.docx

**Session One – Patient-Centered Education for Diagnoses** *(1.5 hours)*

*See PowerPoint notes for more detailed facilitation tips

1. Introduction and pre-survey *(slides 1-3; 5 minutes)*

Slide 1: Explain the etymology of the word “doctor” from the Latin “docere (teach)” and how the goal for the workshop is to aspire students to become medical “teachers” for their patients.

Slide 2: List the objectives.

Slide 3: Handout and have students complete the pre-survey (Appendix B).

1. Didactic lecture: the importance of patient-centered education *(slides 4-10; 10 minutes)*

Slide 4: Discuss how hospitalization and being a patient is an extremely disempowering experience that results in a power differential between patients and providers. A large goal of patient education is to transfer power back to our patients.

Slide 5: Use a real-world example that students may relate to – being “educated” by a car mechanic with confusing mechanic lingo.

Slide 6: Outline the key conclusions from the data that suggest patient education is an important skill.

Slides 7-10: Review key results from the literature that support the conclusions mentioned in slide 6.

1. Interactive discussion: the key elements of patient-centered education *(slides 11-19; 20 minutes)*

Slide 11: Introduce and watch the video (Appendix D), prompting students to watch carefully for positive and negative aspects of patient education that they can discuss and learn from.

Slide 12: Using key takeaways from the video AND student experience, generate two columns on a whiteboard labelled “How to Do” and “How NOT to Do” quality patient-centered education.

Slides 13-14: Reference the whiteboard list of best practices (“How to Do” list) and refer to the assigned pre-reading “Health Literacy in Primary Care Practice” by Hersh et al. (2015) to delineate a list of core practices necessary when providing patient-centered education. At the least, these should include “avoiding medical jargon, breaking down information or instructions into small concrete steps, limiting the focus of a visit to three key points or tasks, and assessing for comprehension.”

Slides 15-16: Further discuss key elements by referring to the Agency for Healthcare Research and Quality (AHRQ) toolkit, specifically focusing on the Teach-Back Method to assess for comprehension. Use example phrases to consider how to assess for comprehension in a non-judgmental and open-ended way.

Slide 17: Have students practice these skills in pairs by translating confusing medical jargon. First, model this skill by explaining a coronary artery bypass graft (CABG) in patient-centered language. After modelling, students should have 4-5 minutes to practice in pairs. Then, have students share their experiences, especially helpful explanations given by their partners. It may be beneficial to ask students at random to share their definitions.

Slides 18-19: Explain that sometimes quick diagrams can also be used as part of patient-centered education. Although students will not have time to practice this skill, model this using the PowerPoint animations to demonstrate educating patients about the clinical course of bipolar disorder (slide 16) and the anatomy of a pneumothorax (slide 17) through simple diagrams.

1. Group role-plays: practicing and demonstrating confidence in patient-centered education *(slides 20-25; 45 minutes)*

Slide 20: Explain the purpose of interactive role-play activities to learn clinical skills. Encourage students to take the activity seriously. Briefly explain the following: groups of 3 students; each student has the opportunity to play the role of doctor, patient, and observer; 3 separate scenarios based on educating the patient about a given diagnosis – either hypertension, diabetes, or depression.

Slides 21-24: Describe the 3 roles in depth using snipped images from the role-play handout materials. It is important to highlight the active role of the observer.

Slide 25: Distribute role-play materials (Appendix E) to each group of 3 students and set a time for students to return to the main room, approximately 45 minutes later. Throughout the exercise, the facilitator may float between rooms to observe and offer real-time feedback.

1. Wrap up and post-survey *(slides 26-29; 10 minutes)*

Slide 26: Solicit feedback from students regarding the lessons learned. Especially consider addressing key challenges and highlighting good performances that were witnessed by facilitators or student observers.

Slide 27: Discuss homework assignments to be completed prior to the second session which include: 1) Practicing these skills with at least one patient within the clerkship; and 2) Researching 3 medications (fluoxetine, metformin, lithium) using the template (Appendix G) to prepare for similar role-play scenarios during the next session.

Slide 28: Handout and have students complete the post-survey (Appendix F).

Slide 29: References.

**Session Two – Patient-Centered Education for Medications** *(1.5 hours)*

*See PowerPoint notes for more detailed facilitation tips

1. Introduction and recap *(slides 1-3; 5 minutes)*

Slide 1: Explain the etymology of the word “doctor” from the Latin “docere (teach)” and how the goal for the workshop is to aspire students to become medical “teachers” for their patients.

Slide 2: List the objectives.

Slide 3: Allow students the chance to share reflections from the first session and lessons learned after practicing these skills with patients in their clerkship in the interim period between sessions.

1. Didactic lecture: definitions and the importance of medication education *(slides 4-9; 10 minutes)*

Slide 4: Explain the relationship between the relevant definitions of patient education, shared decision-making, and informed consent using the Venn Diagram. The focus of this session’s material incorporates all three concepts.

Slide 5: Using the PowerPoint diagram, explain shared decision-making. Highlight the importance of providers creating a safe and open space for patients to share their preferences and values.

Slide 6: Describe the AMA’s definition of informed consent.

Slide 7: Highlight that while formal informed consent may not be legally required for medications, it is ethically required, and ought to be provided at least informally.

Slide 8: Discuss the evidence from the literature suggesting that providers do not educate patients about their treatments adequately or effectively.

Slide 9: Discuss the evidence suggesting that patients want to know fully about their treatments, but also have a hard time remembering things have been told.

1. Interactive discussion: the key elements of medication education *(slides 10-13; 20 minutes)*

Slide 10: Refer back to the pre-reading from the first session to remind students about the key skills for providing patient-centered education, explaining that these still apply fully when discussing treatments and medications.

Slide 11: Highlight the “Integrated Model of Medical Advising” (Feng et al., 2011) to explain the components of discussing medications with patients.

Slide 12: Introduce a conceptual approach to deciding which medication side effects to discuss with patients using the PowerPoint diagram. Patient’s ought to be aware of: 1) Severe but rare side effects, for safety reasons; and 2) Mild but common side effects, for their own expectations.

Slide 13: Have students practice these skills in pairs by explaining various aspects about a medication to their partner using patient-centered language. Allow students 4-5 minutes to practice in pairs. Then, have students share their experiences, especially helpful explanations given by their partners.

1. Group role-plays: practicing and demonstrating confidence in medication education *(slides 14-19; 45 minutes)*

Slide 14: Explain the purpose of interactive role-play activities to learn clinical skills. Encourage students to take the activity seriously. Briefly explain the following: groups of 3 students; each student has the opportunity to play the role of doctor, patient, and observer; 3 separate scenarios based on educating the patient about a given medication – either lithium, metformin, or fluoxetine.

Slides 15-18: Describe the 3 roles in depth using snipped images from the role-play handout materials. It is important to highlight the active role of the observer.

Slide 19: Distribute role-play materials (Appendix I) to each group of 3 students and set a time for students to return to the main room, approximately 45 minutes later. Throughout the exercise, the facilitator may float between rooms to observe and offer real-time feedback.

1. Wrap up and post-survey *(slides 20-22; 10 minutes)*

Slide 20: Solicit feedback from students regarding the lessons learned. Especially consider addressing key challenges and highlighting good performances that were witnessed by facilitators or student observers.

Slide 21: Handout and have students complete the post-survey (Appendix J).

Slide 22: References.

**Potential Modifications:**

1. Content of role-play scenarios could be modified from diagnoses/medications to include informed consent for procedures, explanation of non-surgical interventions, education about general wellness issues, alternative medications etc.
2. Audience could be modified to include faculty, resident physicians, or other healthcare learners (e.g. PA, nursing students) with addition of appropriate and relevant content.
3. Emphasis on role-play scenarios could be decreased by only using one or two scenarios and separating learners into pairs.
4. Workshop could be easily adapted to a virtual platform and aided by the use of the breakout room feature available with many virtual meeting platforms.
