# Supplementary material for: When Doctor Means Teacher: An Interactive Workshop on Patient-Centered Education
Source: MedEdPORTAL. 2020 Dec 10;16:11053. doi: 10.15766/mep_2374-8265.11053 (PMC7732137; doi:10.15766/mep_2374-8265.11053)
Supplement: Supplementary file 1 — Facilitator Guide.docxPresurvey.docxSession 1 Patient Education Diagnoses.pptxVideo.mp4Session 1 Role-Play Scenarios.docxSession 1 Postsurvey.docxMedication Research Worksheet.docxSession 2 Patient Education Medications.pptxSession 2 Role-Play Scenarios.docxSession 2 Postsurvey.docx [file mep_2374-8265.11053-s001.zip › B. Presurvey.docx]

**Patient-Centered Education Workshop – PRE-Survey**

Please rate your agreement with the following statements (circle your answers):

1. **Delivering patient-centered education is an important skill for health care providers.**

Strongly Disagree Disagree Neutral Agree Strongly Agree

1. **I can name the important elements of patient-centered education.**

Strongly Disagree Disagree Neutral Agree Strongly Agree

1. **I am confident in my ability to deliver patient-centered education.**

Strongly Disagree Disagree Neutral Agree Strongly Agree

1. **I have had opportunities to practice delivering patient-centered education.**

Strongly Disagree Disagree Neutral Agree Strongly Agree

1. **Role-plays are an effective tool for learning patient-centered education.**

Strongly Disagree Disagree Neutral Agree Strongly Agree

- - - - - - - - - - - - - - - - - - - - - - - - - - - - - - - - - - - - - - - - - - - - - - - - - - - - - - - - - - - - - - - - - - - - - - - - - - - - - - - - - - - -
