# Supplementary material for: When Doctor Means Teacher: An Interactive Workshop on Patient-Centered Education
Source: MedEdPORTAL. 2020 Dec 10;16:11053. doi: 10.15766/mep_2374-8265.11053 (PMC7732137; doi:10.15766/mep_2374-8265.11053)
Supplement: Supplementary file 1 — Facilitator Guide.docxPresurvey.docxSession 1 Patient Education Diagnoses.pptxVideo.mp4Session 1 Role-Play Scenarios.docxSession 1 Postsurvey.docxMedication Research Worksheet.docxSession 2 Patient Education Medications.pptxSession 2 Role-Play Scenarios.docxSession 2 Postsurvey.docx [file mep_2374-8265.11053-s001.zip › C. Session 1 Patient Education Diagnoses.pptx]

## Slide 1
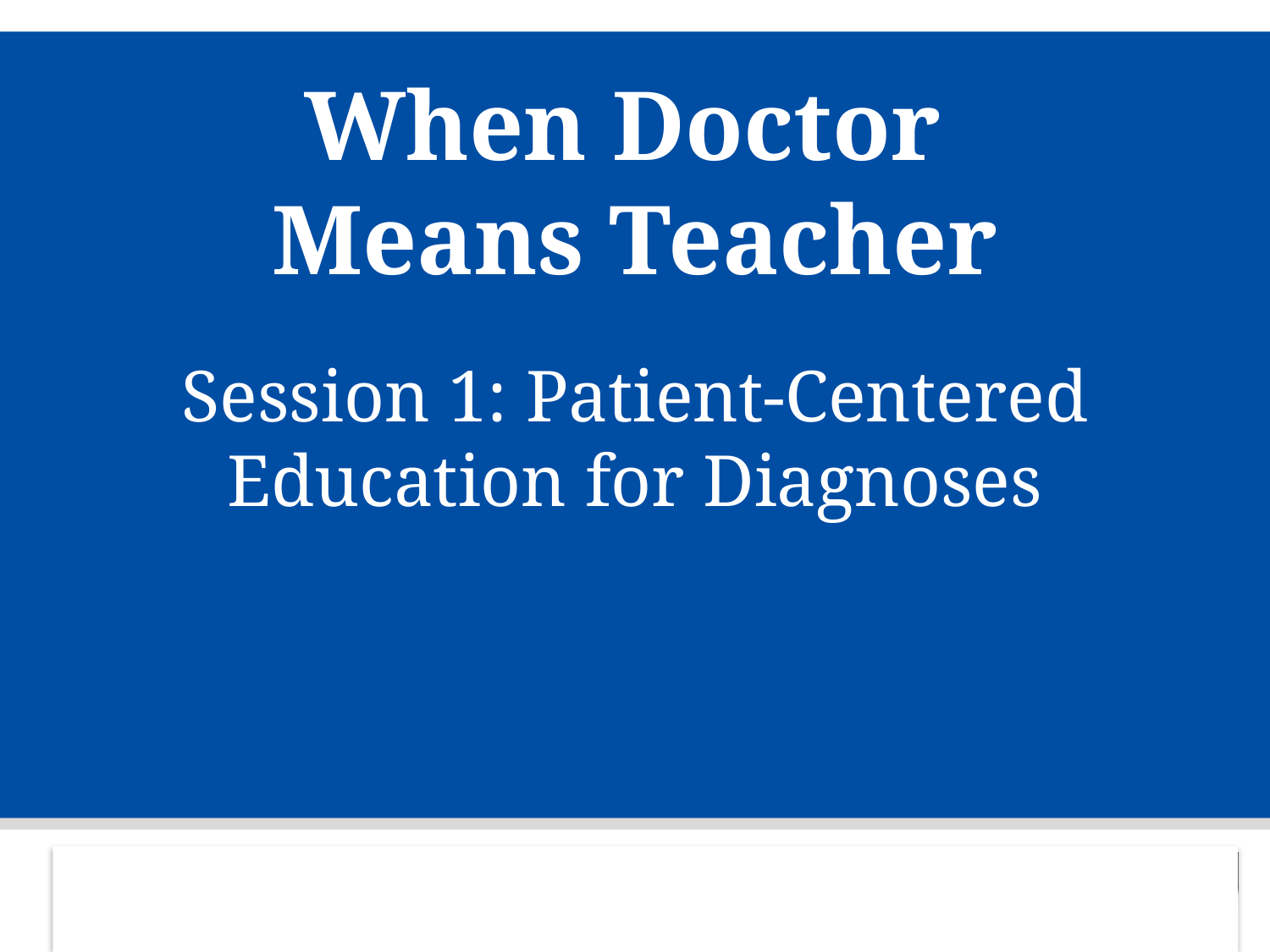

# When Doctor Means Teacher
Session 1: Patient-Centered Education for Diagnoses

## Slide 2
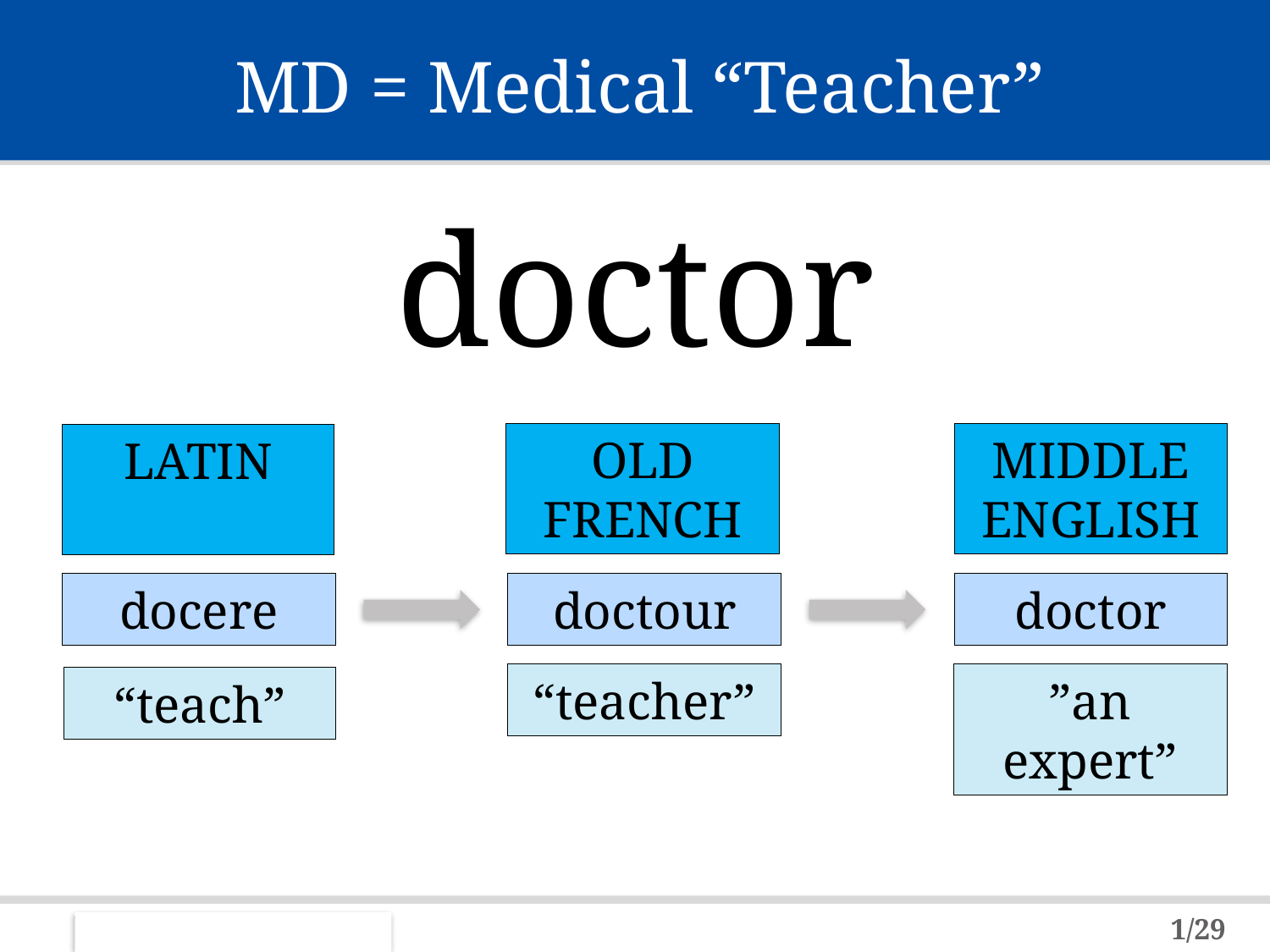

# MD = Medical “Teacher”
MIDDLE ENGLISH
LATIN
OLD FRENCH
docere
doctor
doctour
“teacher”
”an expert”
“teach”
1/29

## Slide 3
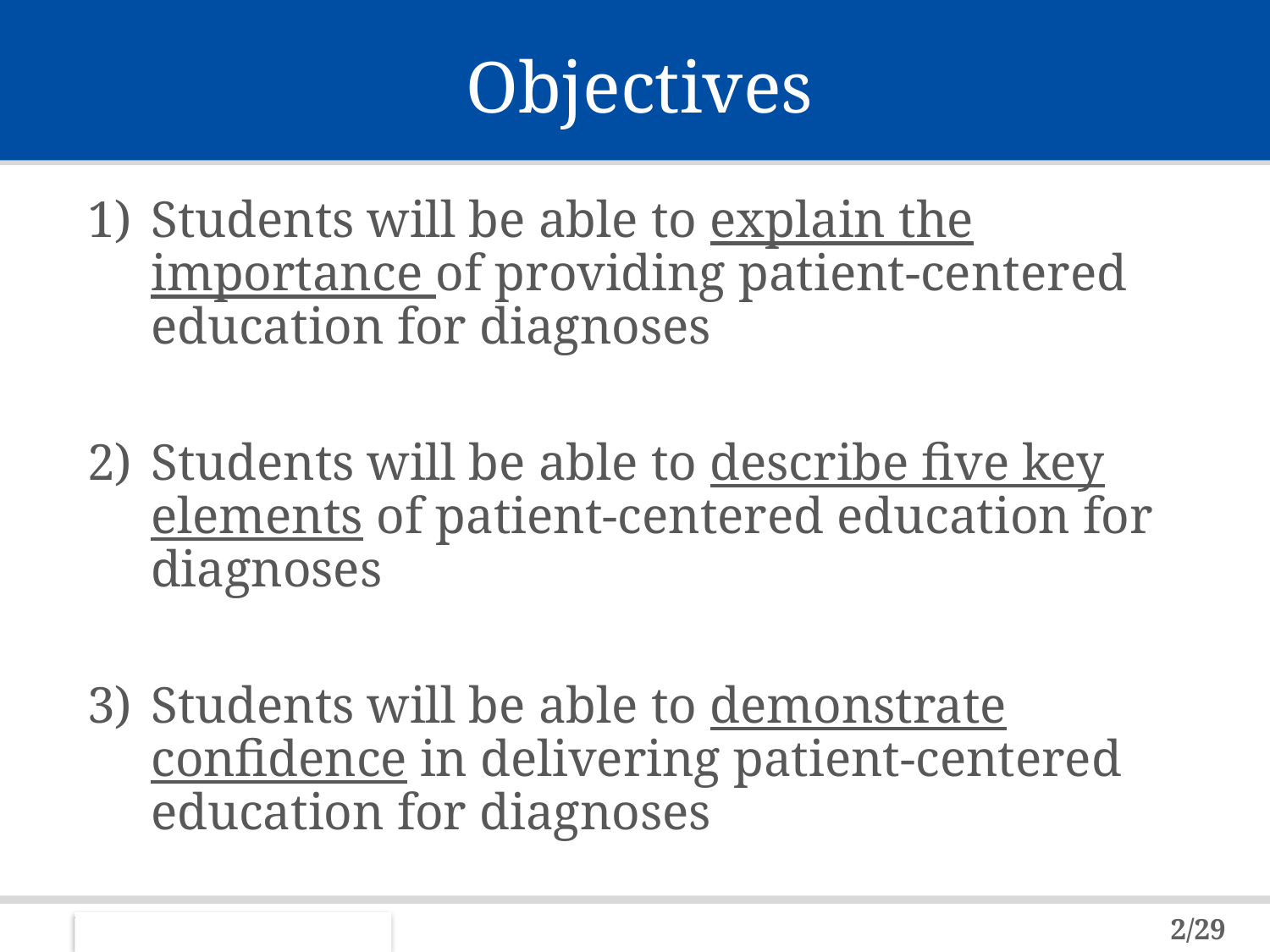

# Objectives
Students will be able to explain the importance of providing patient-centered education for diagnoses
Students will be able to describe five key elements of patient-centered education for diagnoses
Students will be able to demonstrate confidence in delivering patient-centered education for diagnoses
2/29

## Slide 4
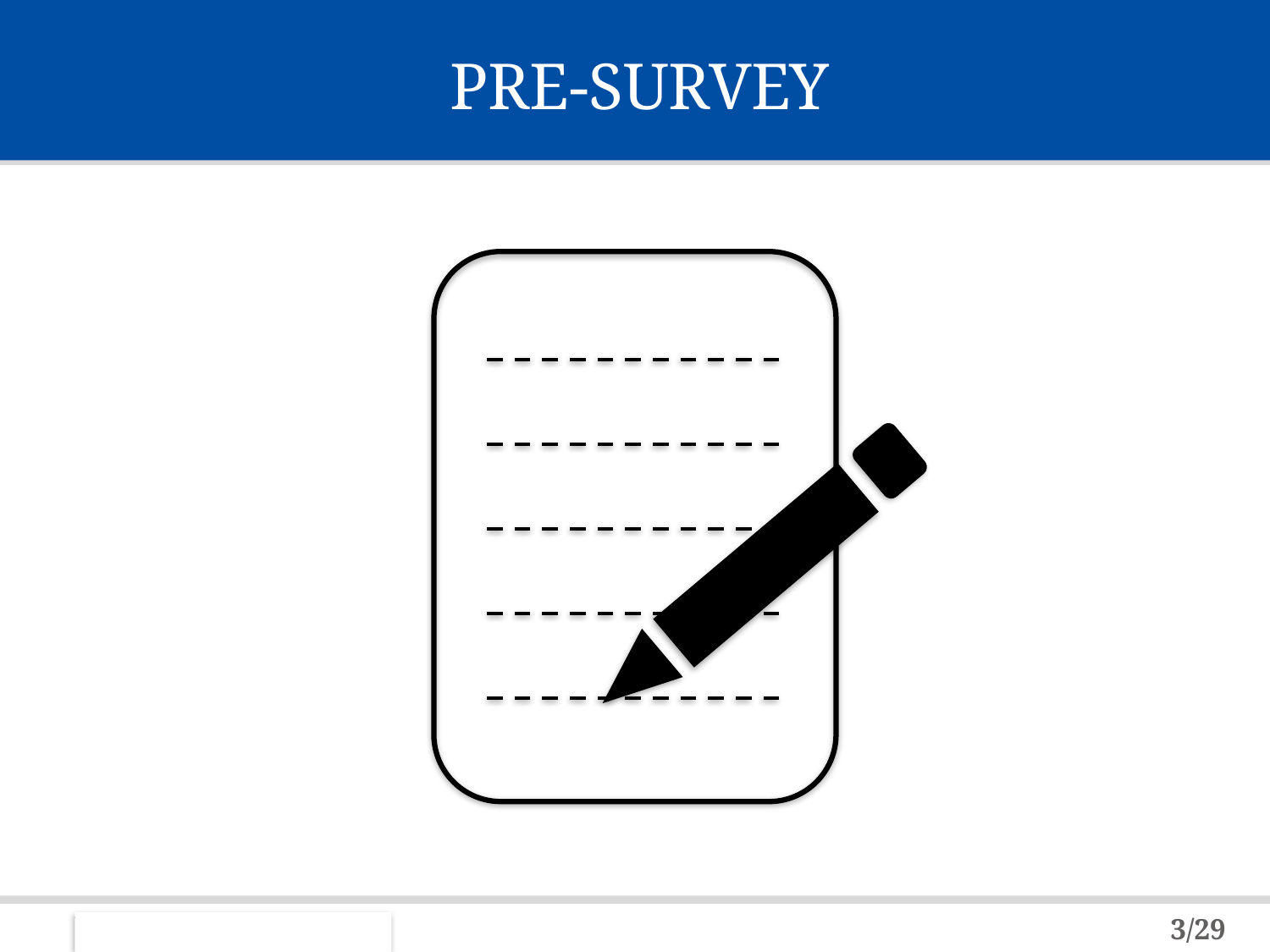

# PRE-SURVEY
3/29

## Slide 5
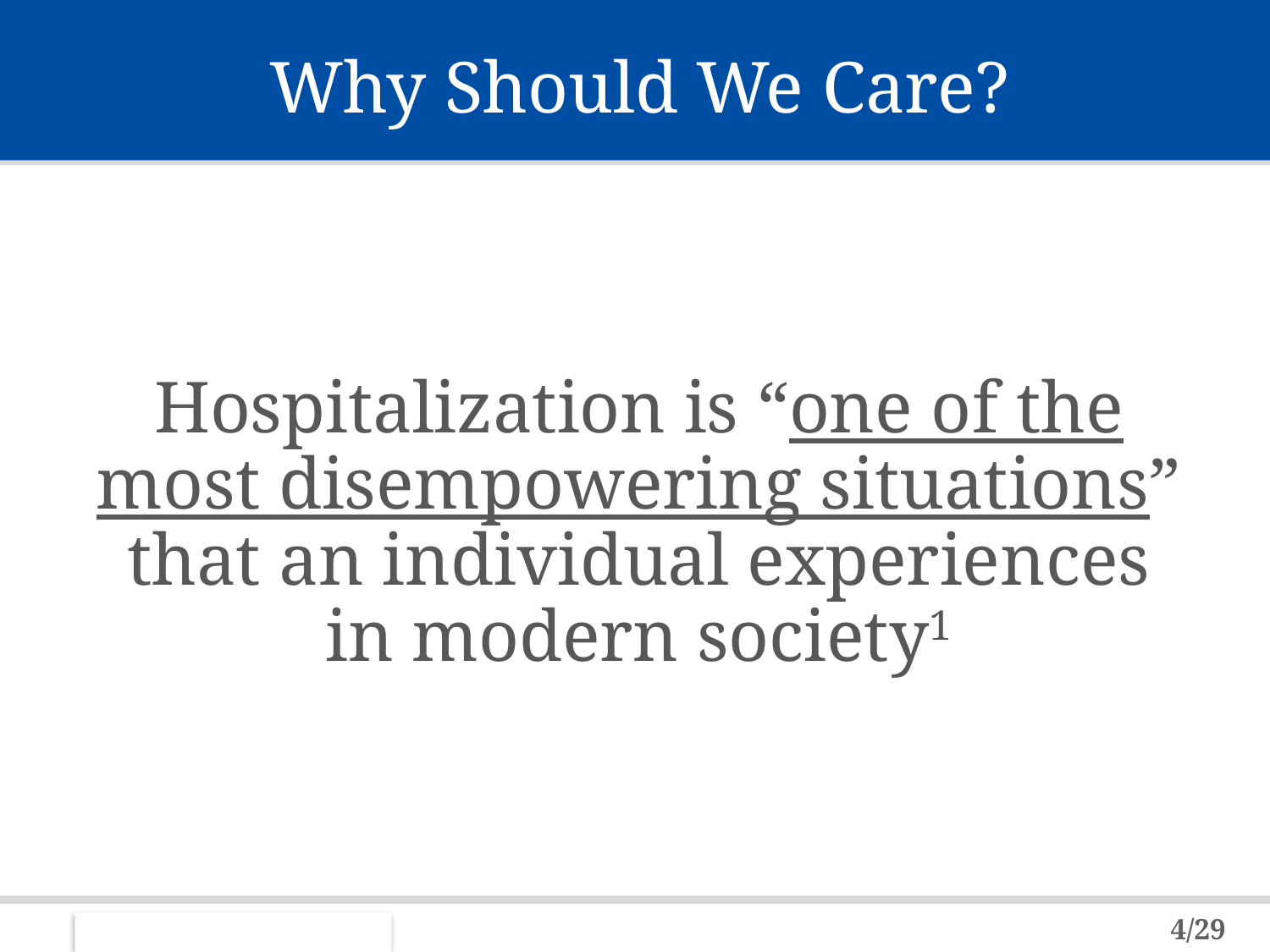

# Why Should We Care?
Hospitalization is “one of the most disempowering situations” that an individual experiences in modern society1
4/29

## Slide 6
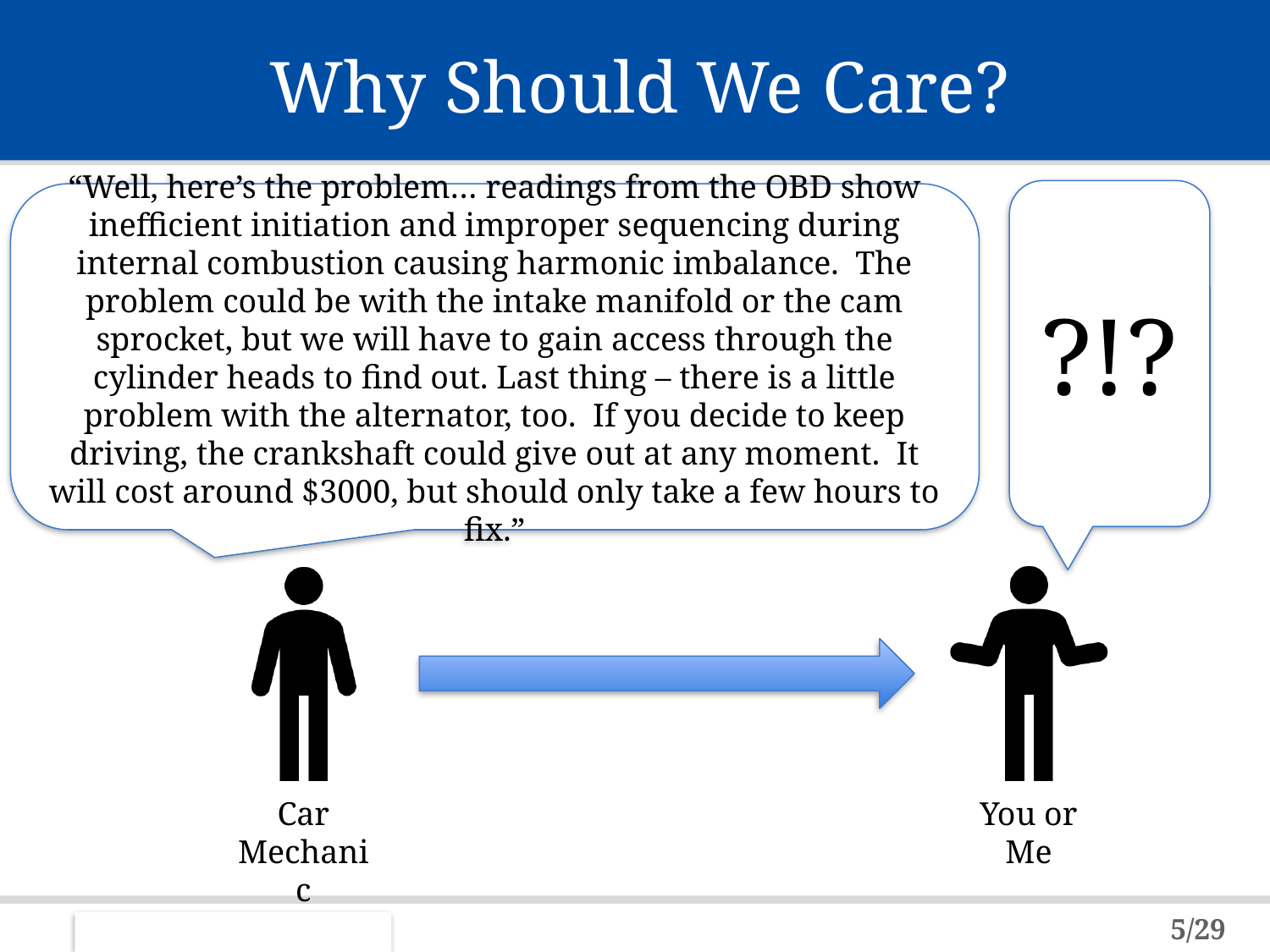

# Why Should We Care?
?!?
“Well, here’s the problem… readings from the OBD show inefficient initiation and improper sequencing during internal combustion causing harmonic imbalance. The problem could be with the intake manifold or the cam sprocket, but we will have to gain access through the cylinder heads to find out. Last thing – there is a little problem with the alternator, too. If you decide to keep driving, the crankshaft could give out at any moment. It will cost around $3000, but should only take a few hours to fix.”
Car
Mechanic
You or Me
5/29

## Slide 7
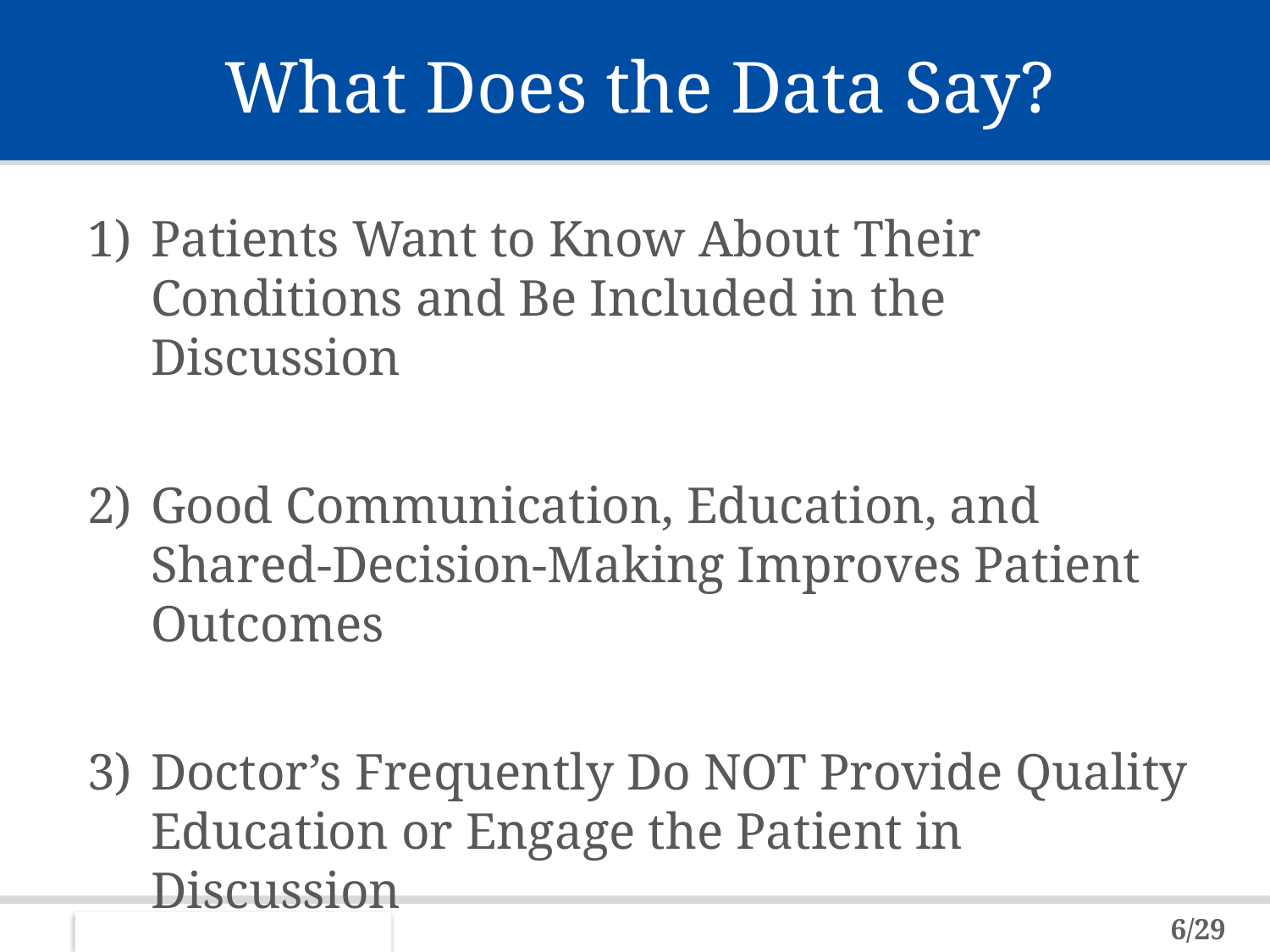

# What Does the Data Say?
Patients Want to Know About Their Conditions and Be Included in the Discussion
Good Communication, Education, and Shared-Decision-Making Improves Patient Outcomes
Doctor’s Frequently Do NOT Provide Quality Education or Engage the Patient in Discussion
6/29

## Slide 8
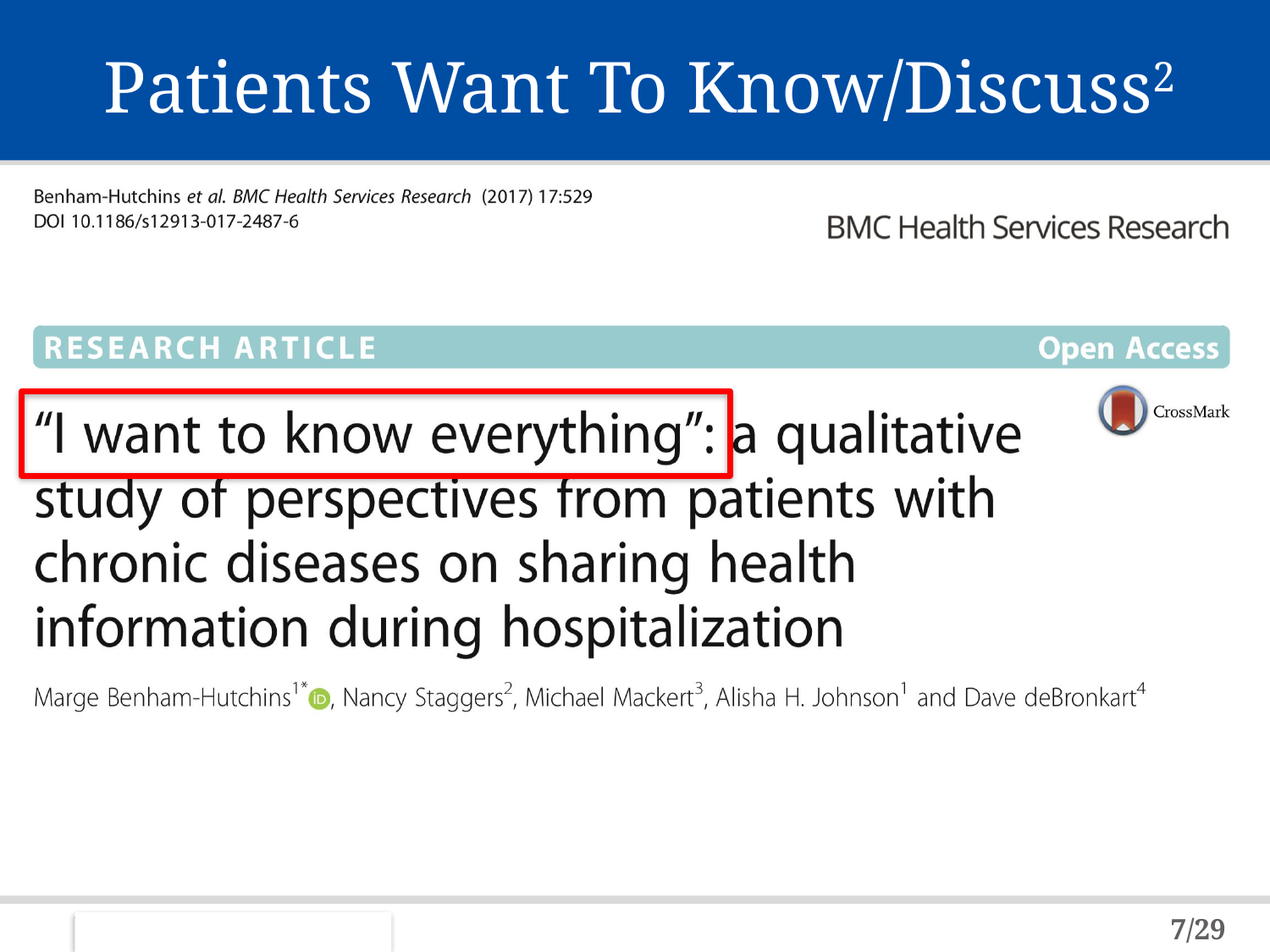

# Patients Want To Know/Discuss2
7/29

## Slide 9
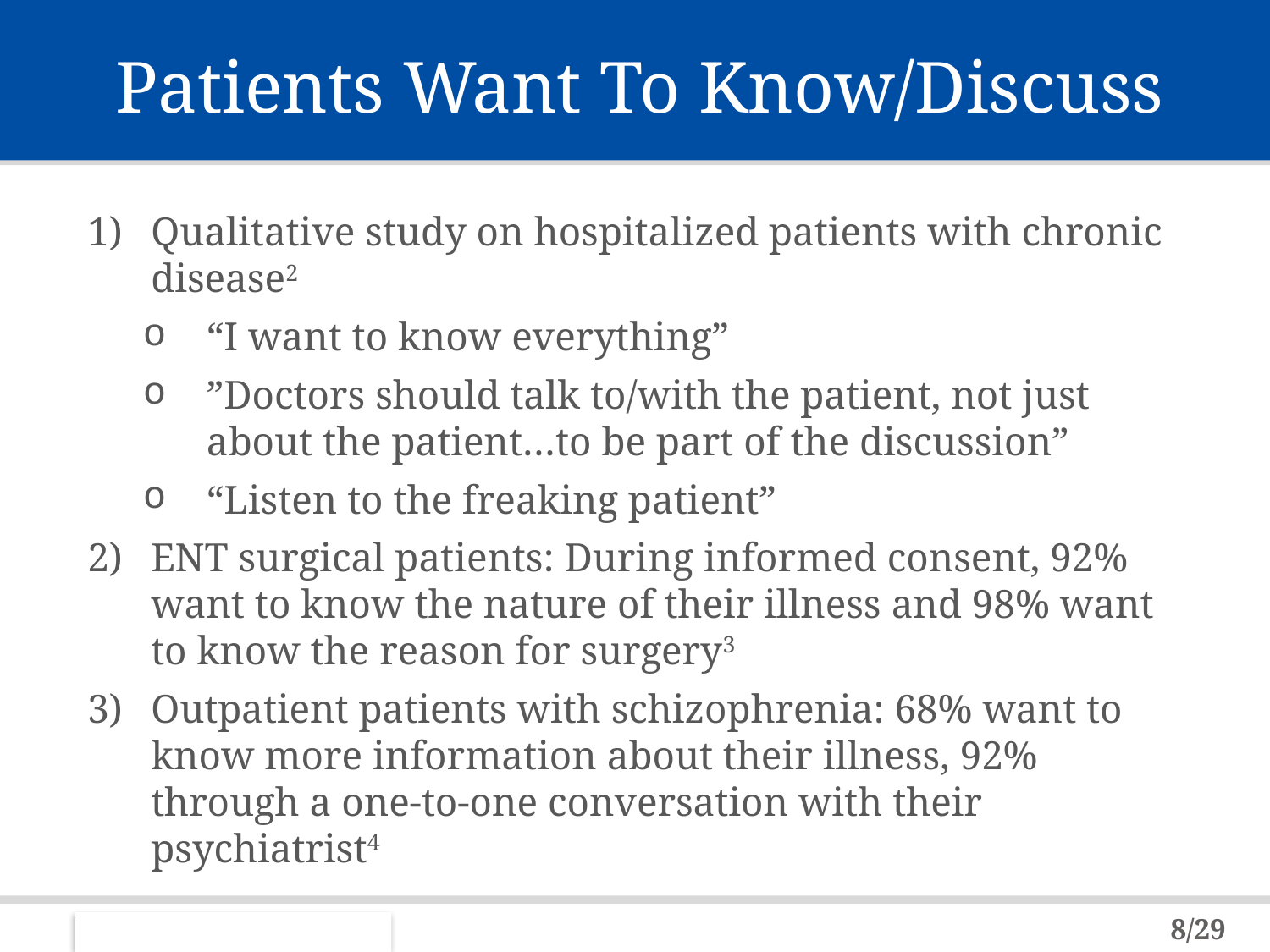

# Patients Want To Know/Discuss
Qualitative study on hospitalized patients with chronic disease2
“I want to know everything”
”Doctors should talk to/with the patient, not just about the patient…to be part of the discussion”
“Listen to the freaking patient”
ENT surgical patients: During informed consent, 92% want to know the nature of their illness and 98% want to know the reason for surgery3
Outpatient patients with schizophrenia: 68% want to know more information about their illness, 92% through a one-to-one conversation with their psychiatrist4
8/29

## Slide 10
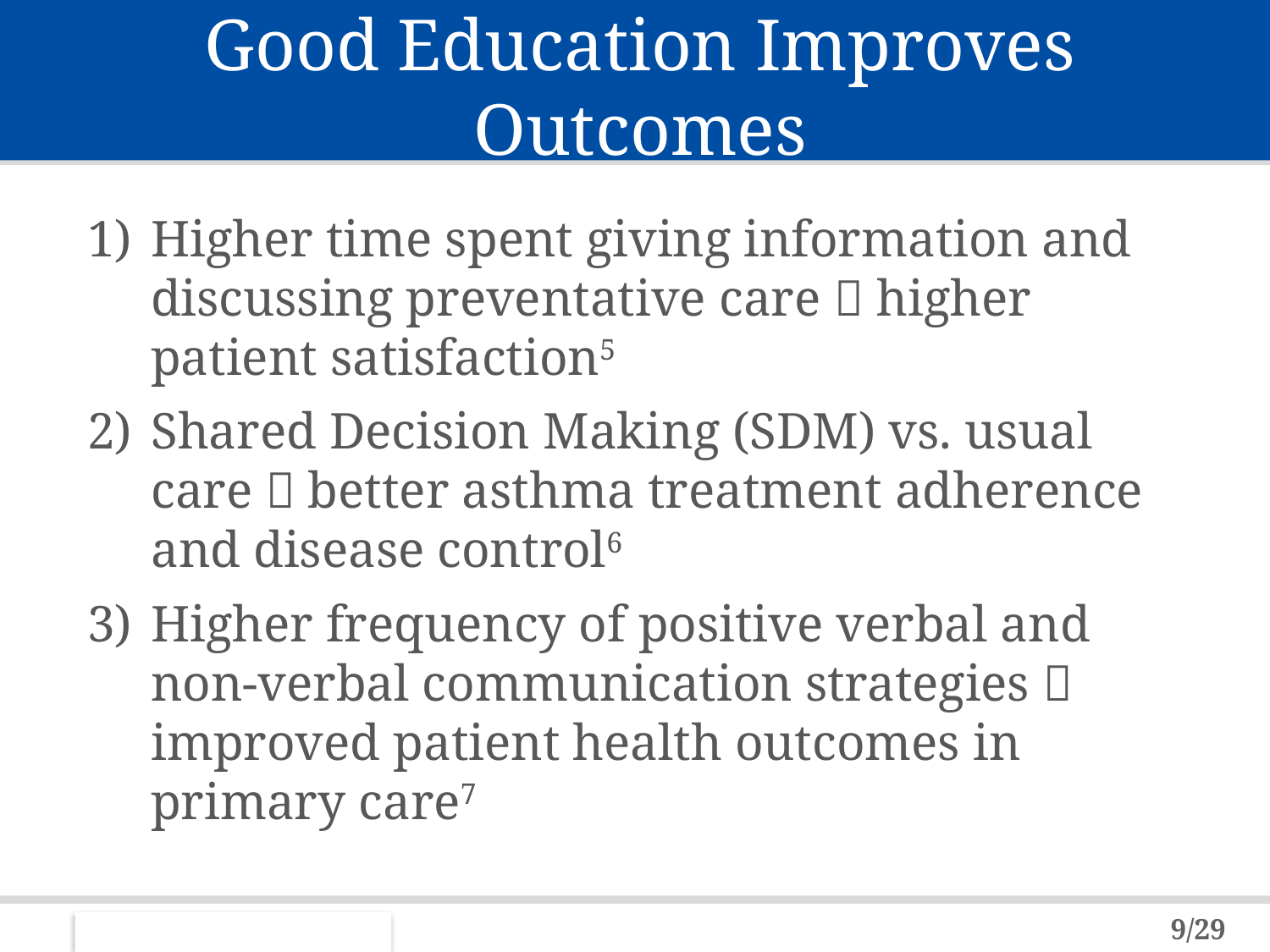

# Good Education Improves Outcomes
Higher time spent giving information and discussing preventative care  higher patient satisfaction5
Shared Decision Making (SDM) vs. usual care  better asthma treatment adherence and disease control6
Higher frequency of positive verbal and non-verbal communication strategies  improved patient health outcomes in primary care7
9/29

## Slide 11
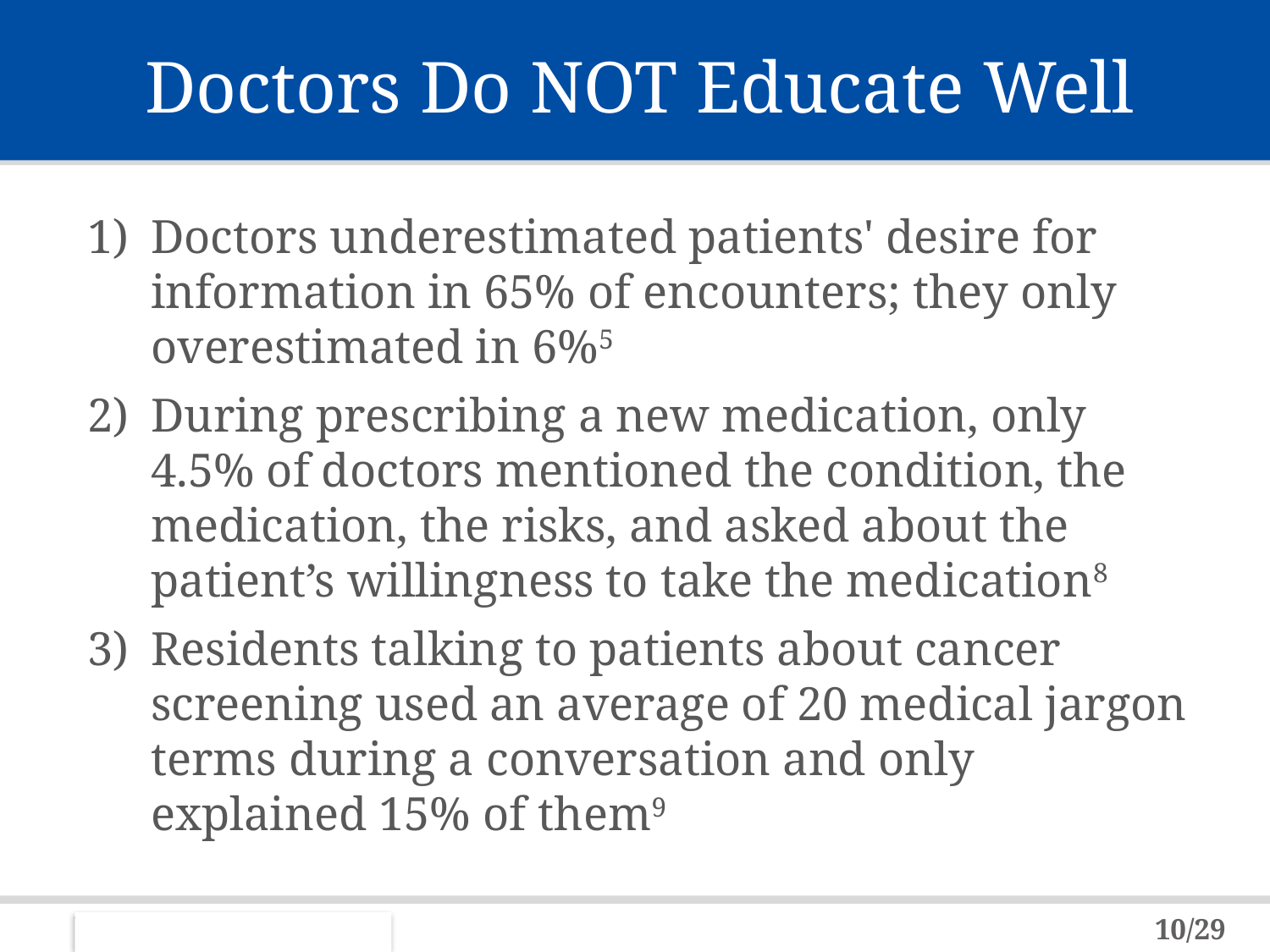

# Doctors Do NOT Educate Well
Doctors underestimated patients' desire for information in 65% of encounters; they only overestimated in 6%5
During prescribing a new medication, only 4.5% of doctors mentioned the condition, the medication, the risks, and asked about the patient’s willingness to take the medication8
Residents talking to patients about cancer screening used an average of 20 medical jargon terms during a conversation and only explained 15% of them9
10/29

## Slide 12
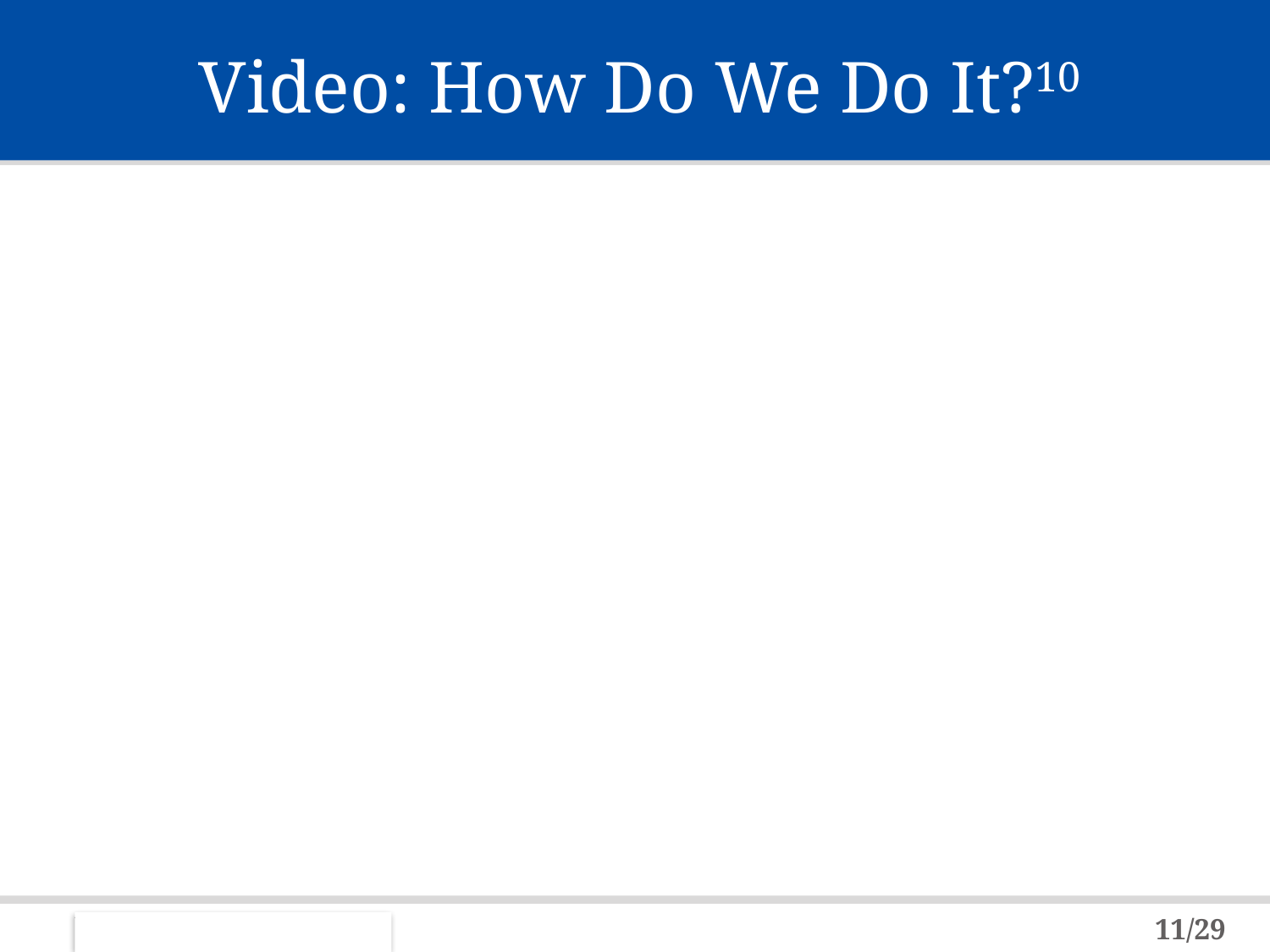

# Video: How Do We Do It?10
11/29

## Slide 13
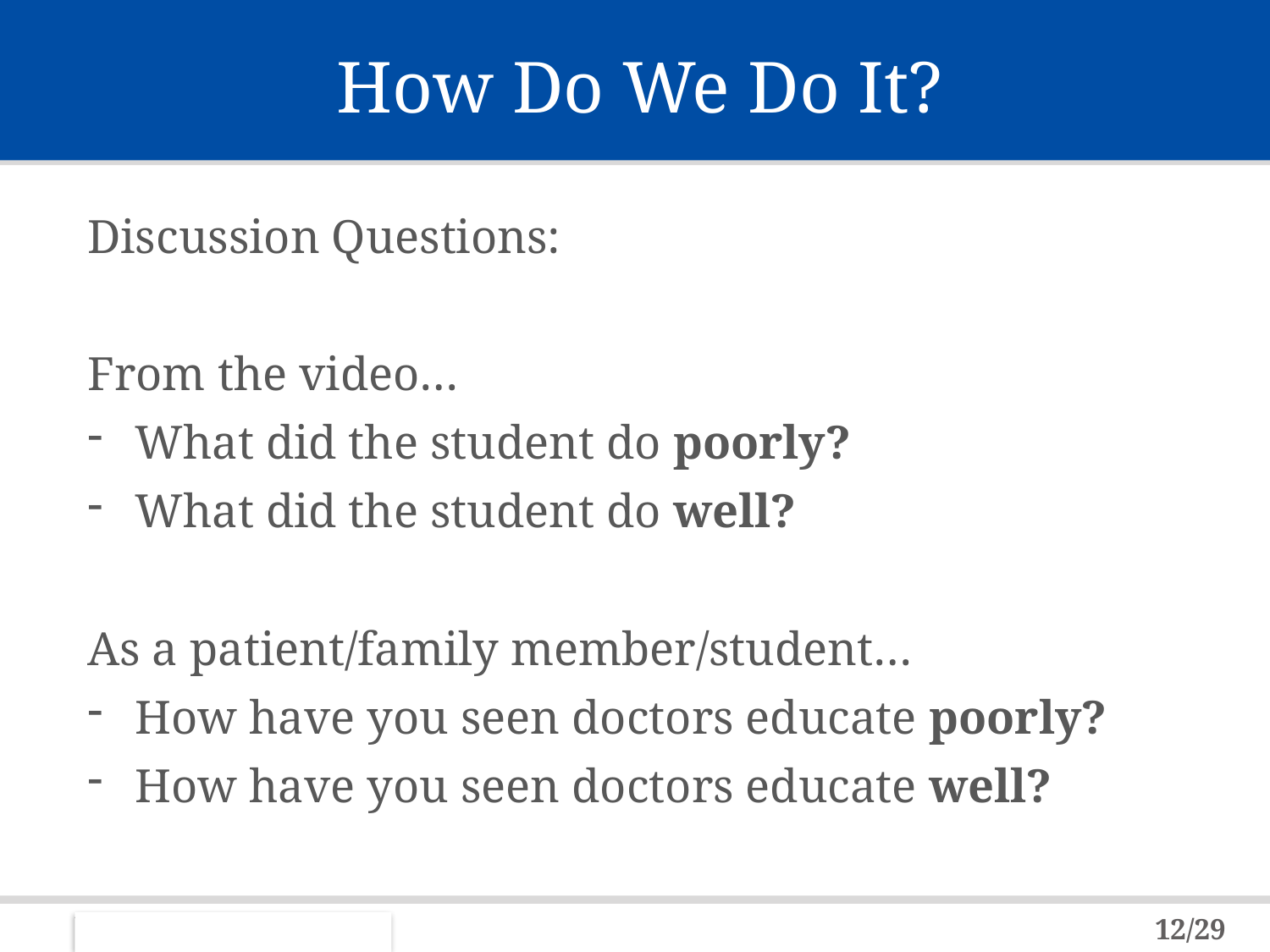

# How Do We Do It?
Discussion Questions:
From the video…
What did the student do poorly?
What did the student do well?
As a patient/family member/student…
How have you seen doctors educate poorly?
How have you seen doctors educate well?
12/29

## Slide 14
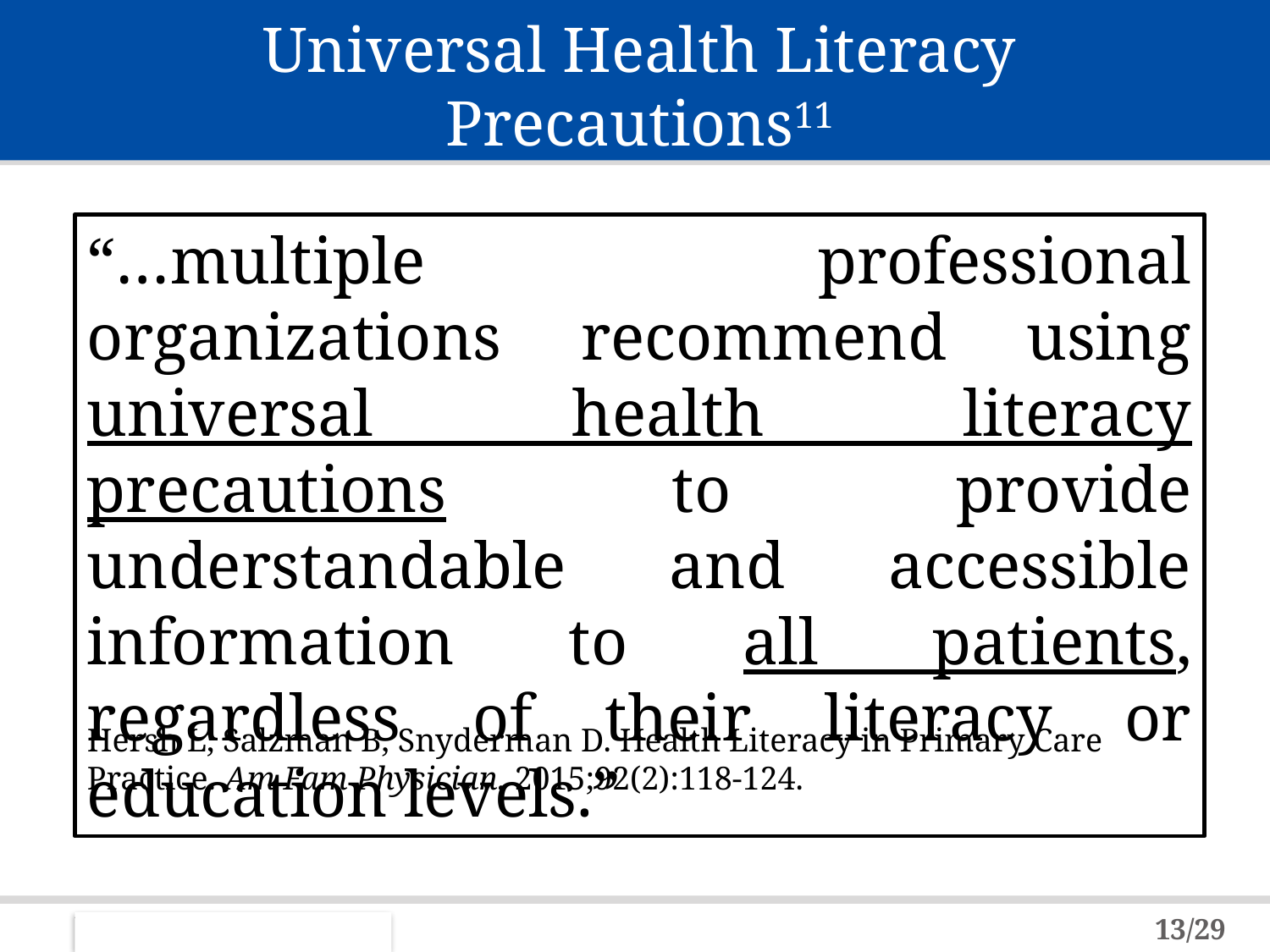

# Universal Health Literacy Precautions11
“…multiple professional organizations recommend using universal health literacy precautions to provide understandable and accessible information to all patients, regardless of their literacy or education levels.”
Hersh L, Salzman B, Snyderman D. Health Literacy in Primary Care Practice. Am Fam Physician. 2015;92(2):118-124.
13/29

## Slide 15
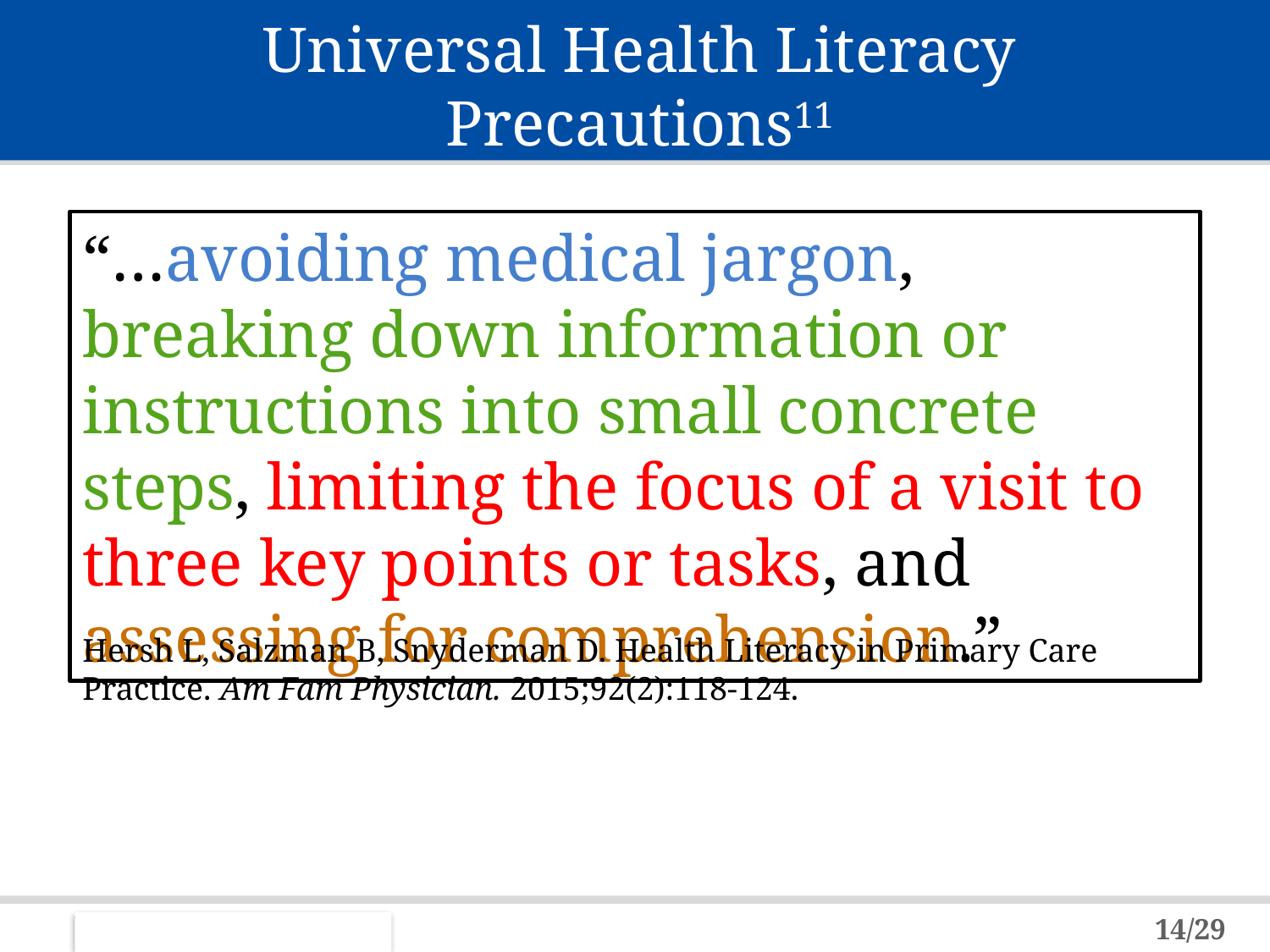

# Universal Health Literacy Precautions11
“…avoiding medical jargon, breaking down information or instructions into small concrete steps, limiting the focus of a visit to three key points or tasks, and assessing for comprehension.”
Hersh L, Salzman B, Snyderman D. Health Literacy in Primary Care Practice. Am Fam Physician. 2015;92(2):118-124.
14/29

## Slide 16
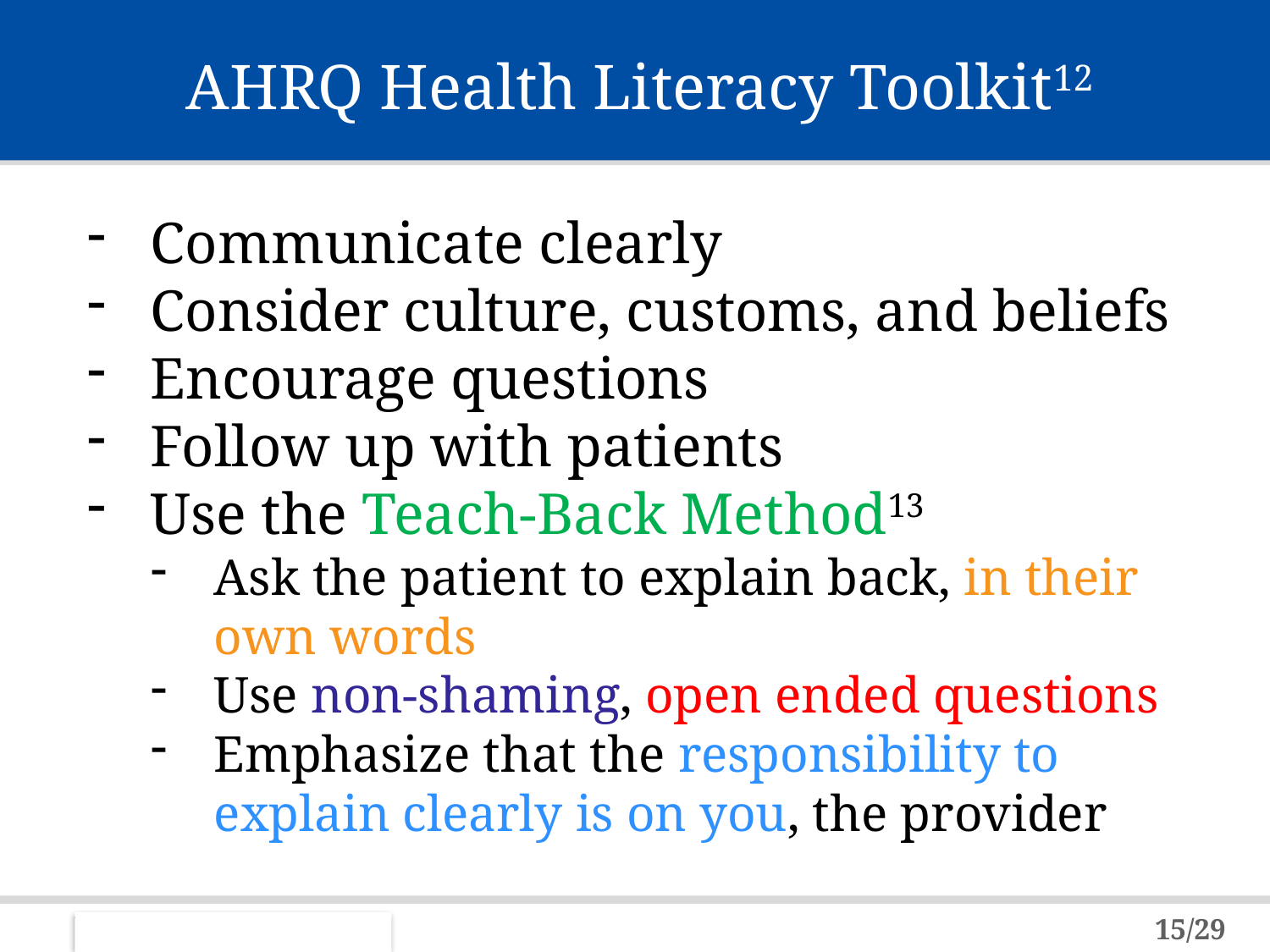

# AHRQ Health Literacy Toolkit12
Communicate clearly
Consider culture, customs, and beliefs
Encourage questions
Follow up with patients
Use the Teach-Back Method13
Ask the patient to explain back, in their own words
Use non-shaming, open ended questions
Emphasize that the responsibility to explain clearly is on you, the provider
15/29

## Slide 17
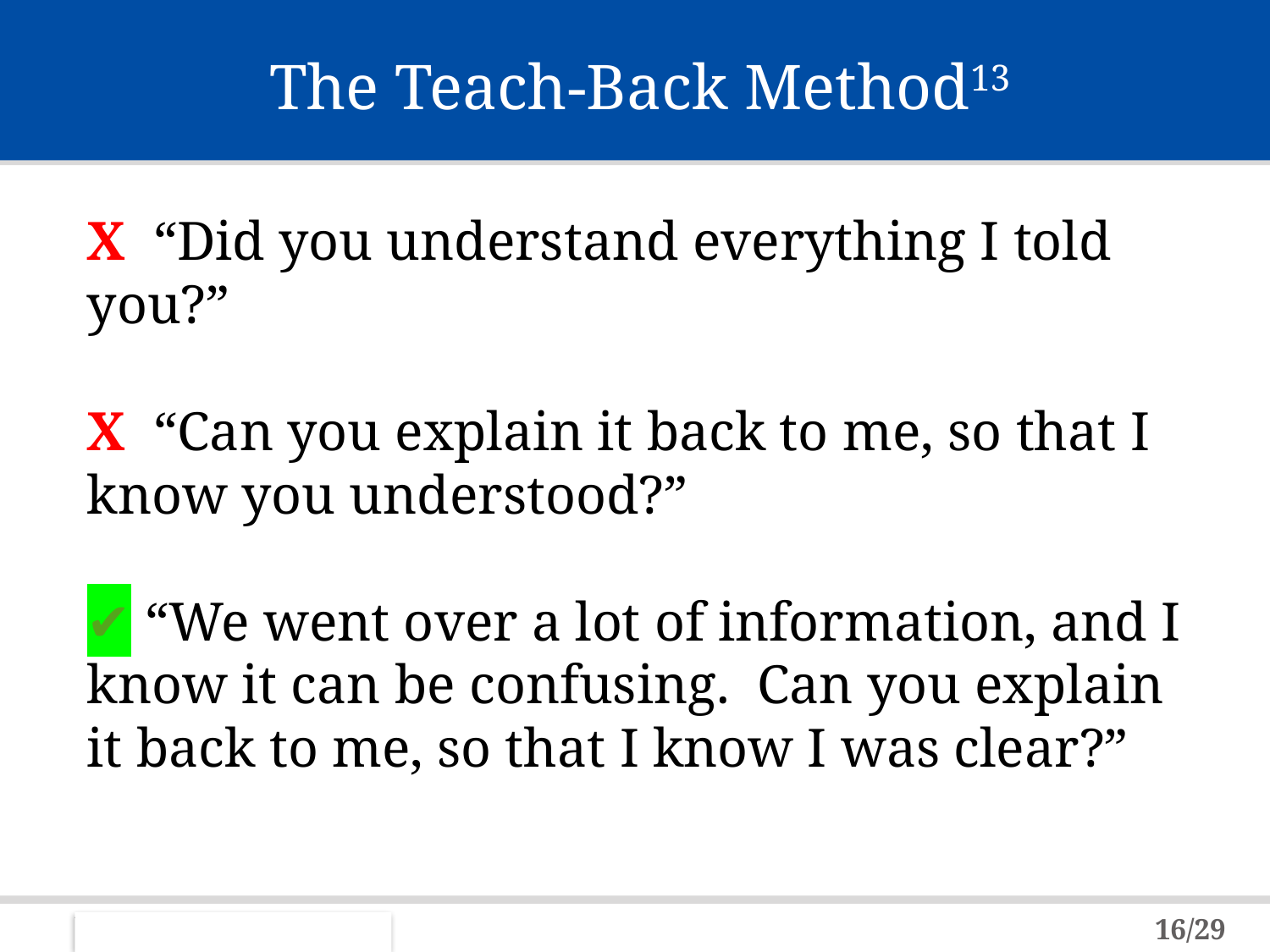

# The Teach-Back Method13
X “Did you understand everything I told you?”
X “Can you explain it back to me, so that I know you understood?”
✔️ “We went over a lot of information, and I know it can be confusing. Can you explain it back to me, so that I know I was clear?”
16/29

## Slide 18
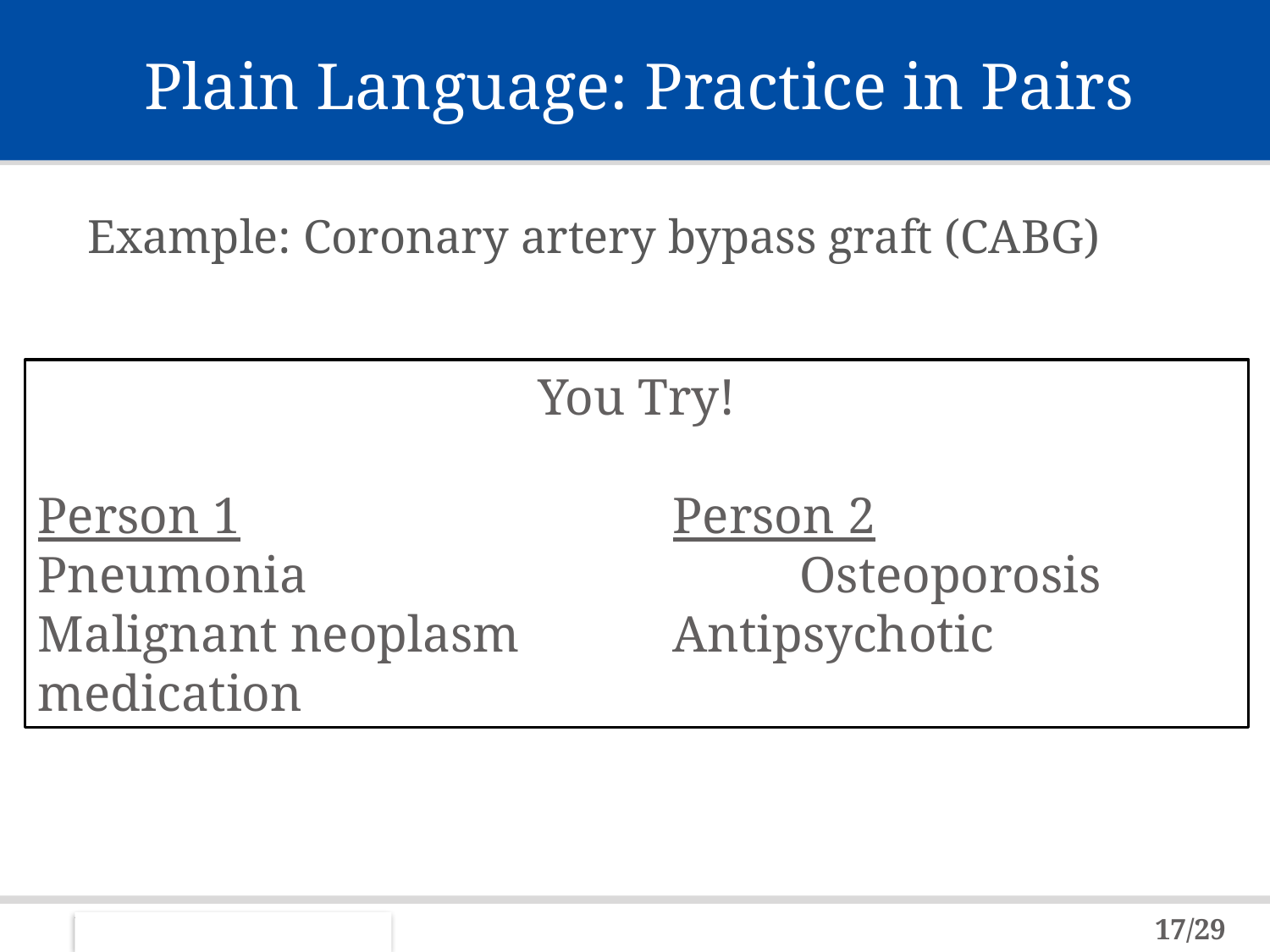

# Plain Language: Practice in Pairs
Example: Coronary artery bypass graft (CABG)
You Try!
Person 1				Person 2
Pneumonia				Osteoporosis
Malignant neoplasm		Antipsychotic medication
17/29

## Slide 19
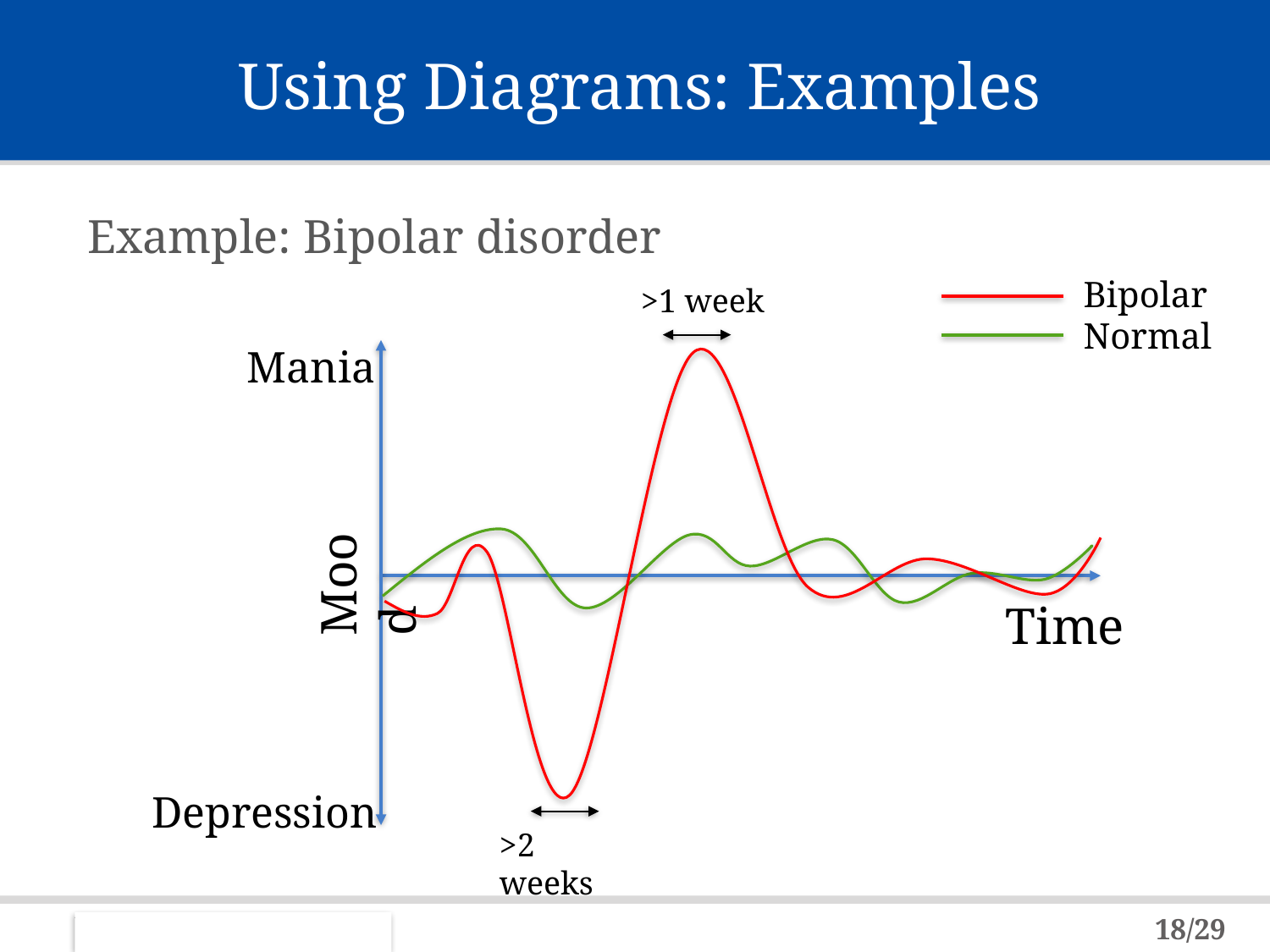

# Using Diagrams: Examples
Example: Bipolar disorder
Bipolar
>1 week
Normal
Mania
Mood
Time
Depression
>2 weeks
18/29

## Slide 20
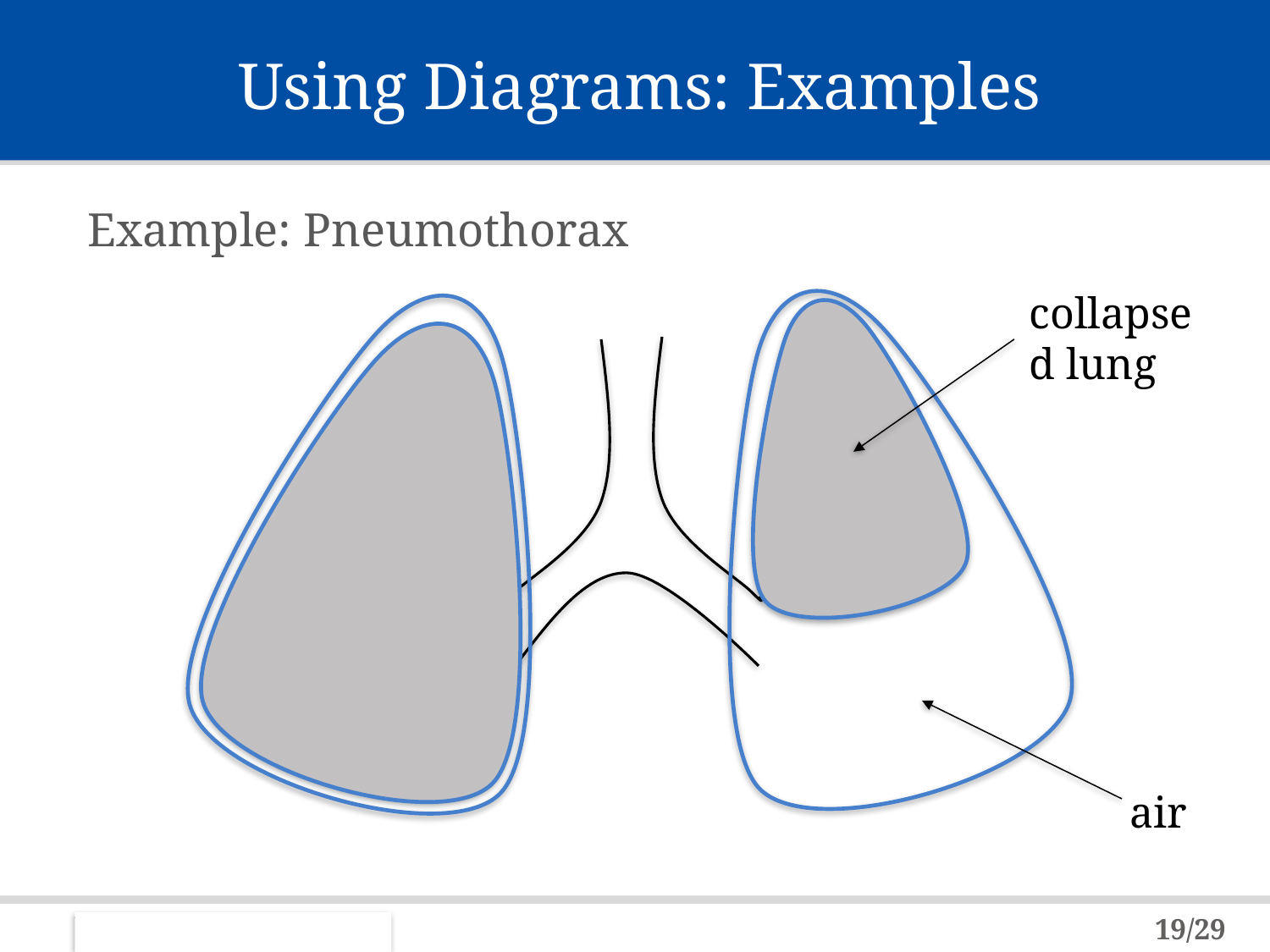

# Using Diagrams: Examples
Example: Pneumothorax
collapsed lung
air
19/29

## Slide 21
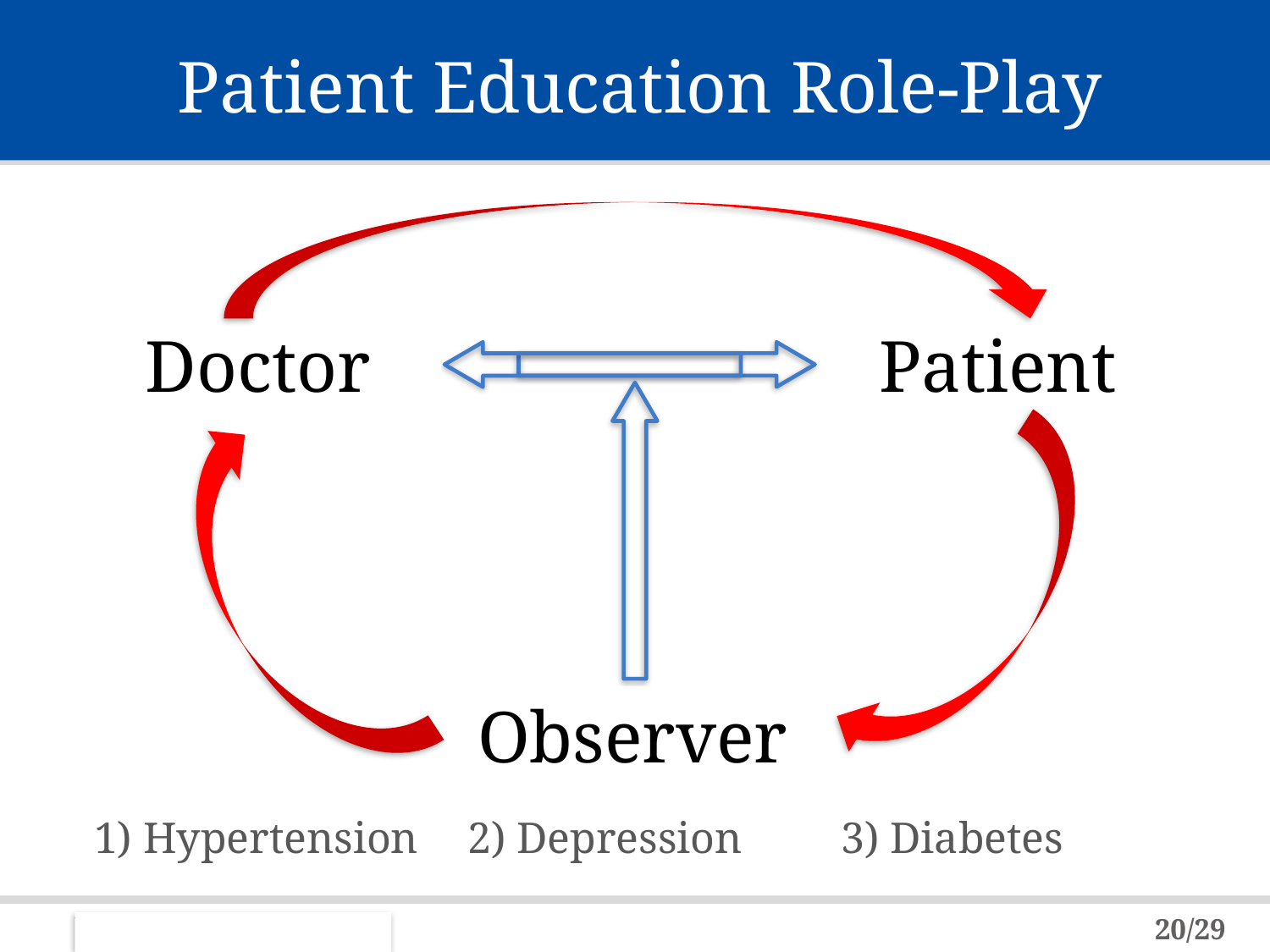

# Patient Education Role-Play
Doctor
Patient
Observer
2) Depression
3) Diabetes
1) Hypertension
20/29

## Slide 22
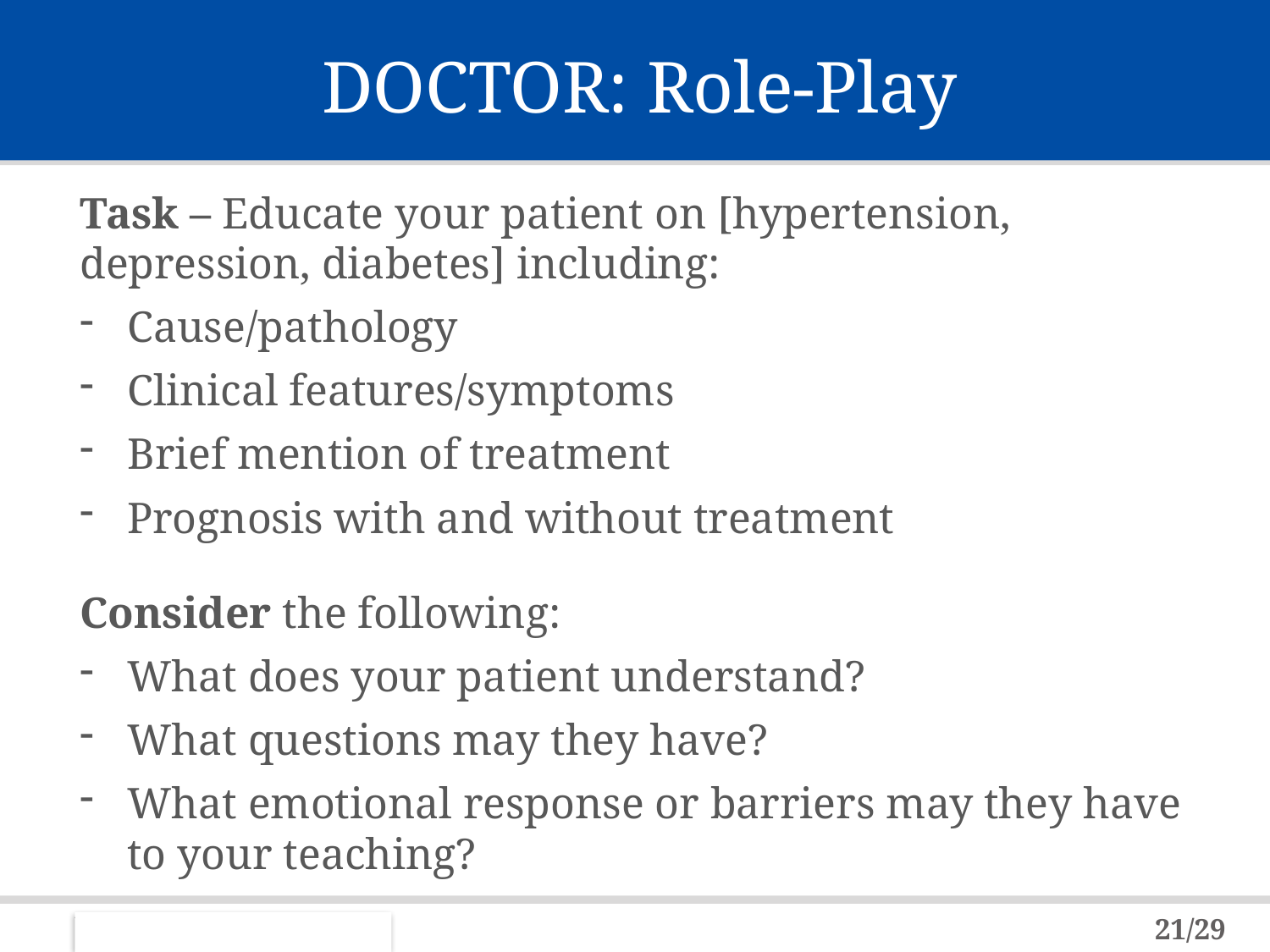

# DOCTOR: Role-Play
Task – Educate your patient on [hypertension, depression, diabetes] including:
Cause/pathology
Clinical features/symptoms
Brief mention of treatment
Prognosis with and without treatment
Consider the following:
What does your patient understand?
What questions may they have?
What emotional response or barriers may they have to your teaching?
21/29

## Slide 23
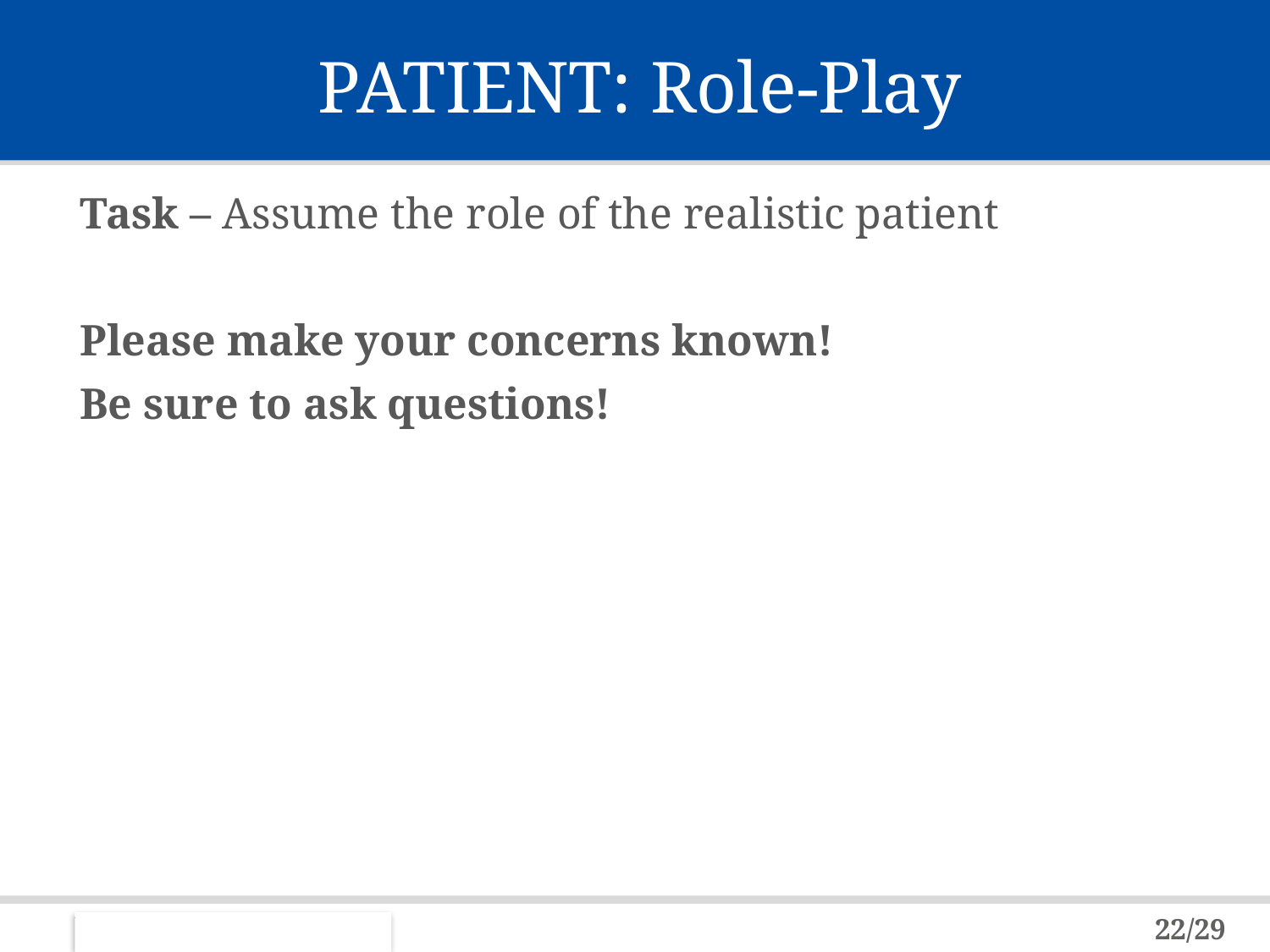

# PATIENT: Role-Play
Task – Assume the role of the realistic patient
Please make your concerns known!
Be sure to ask questions!
22/29

## Slide 24
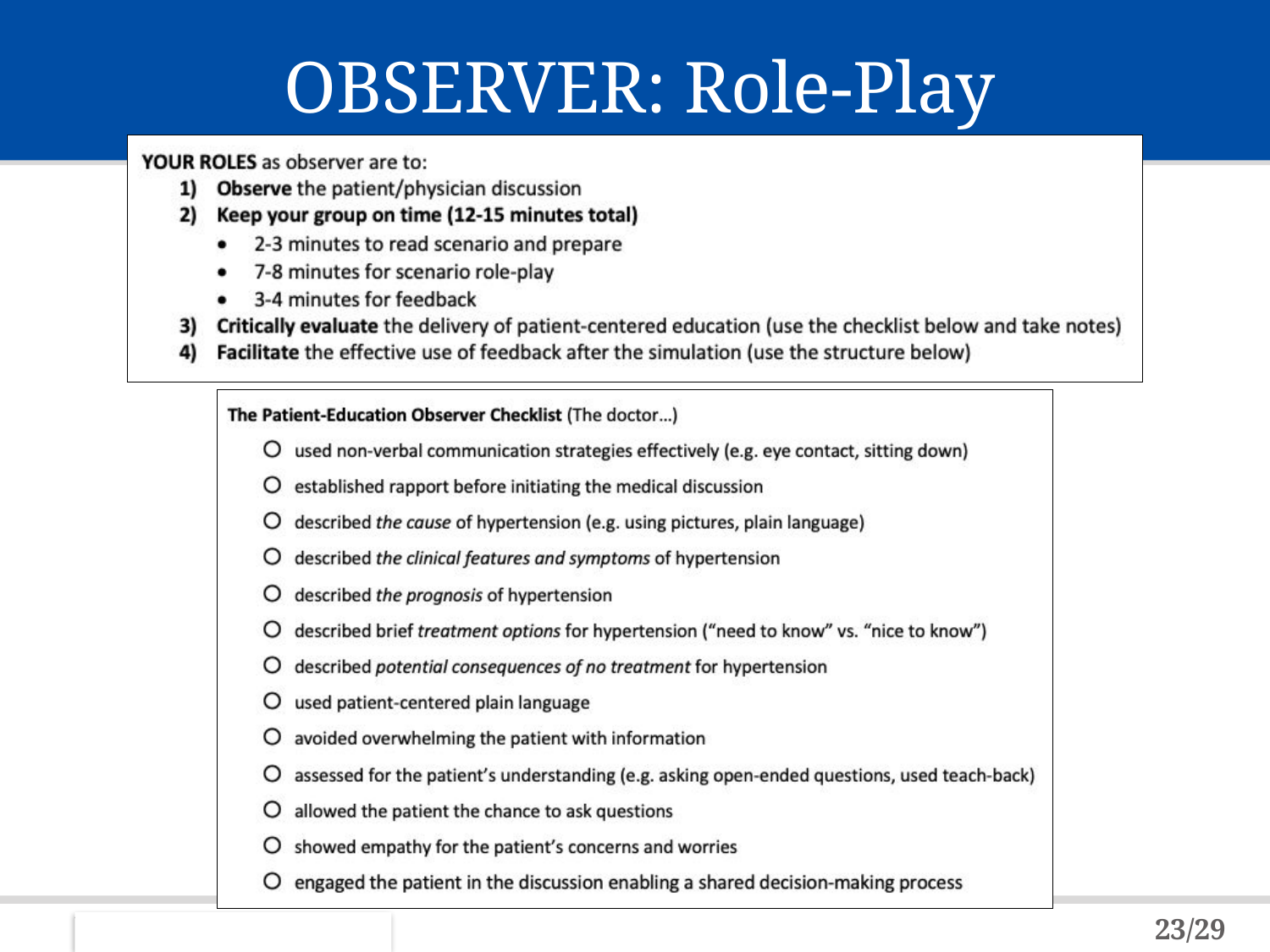

# OBSERVER: Role-Play
23/29

## Slide 25
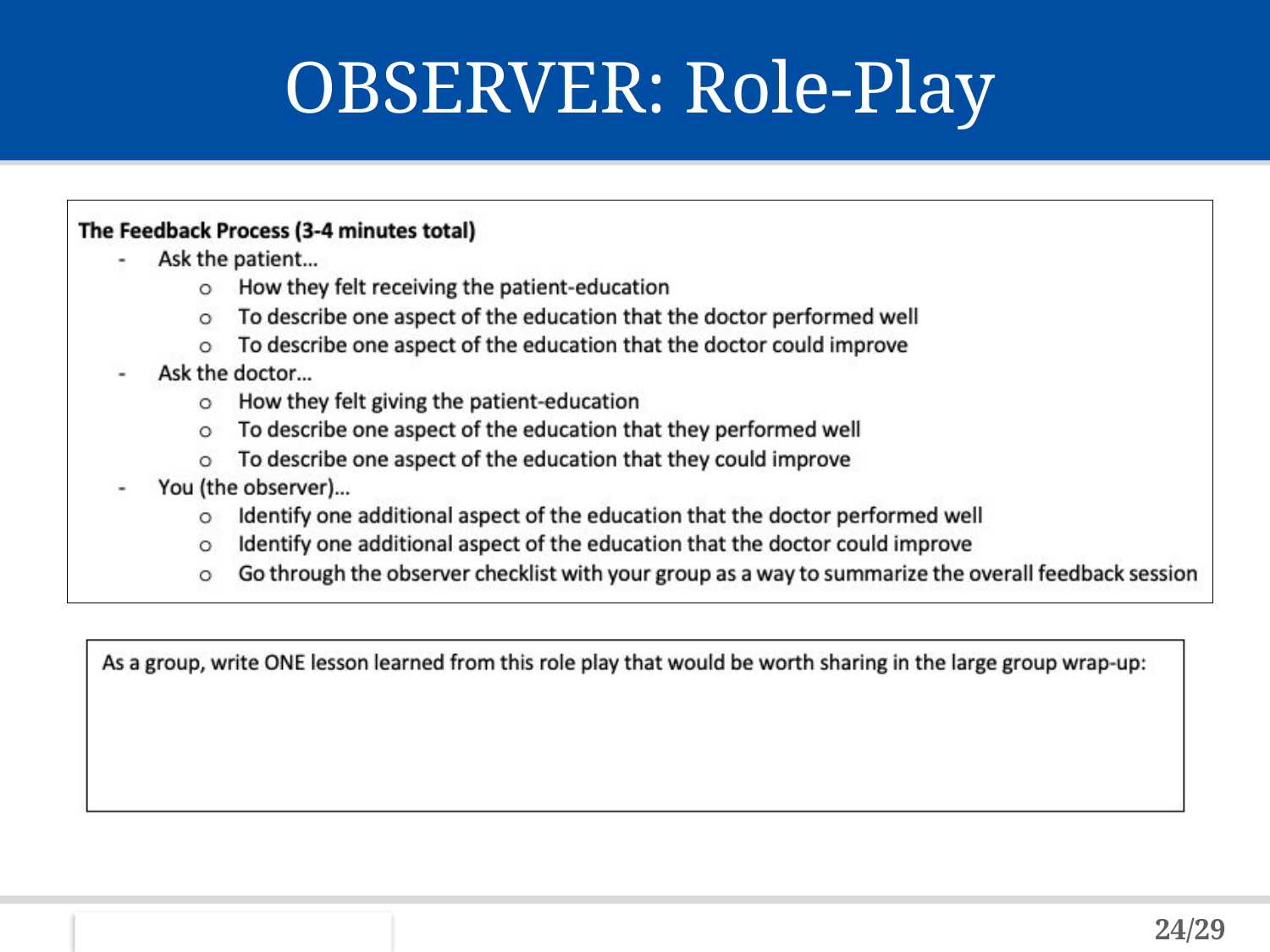

# OBSERVER: Role-Play
24/29

## Slide 26
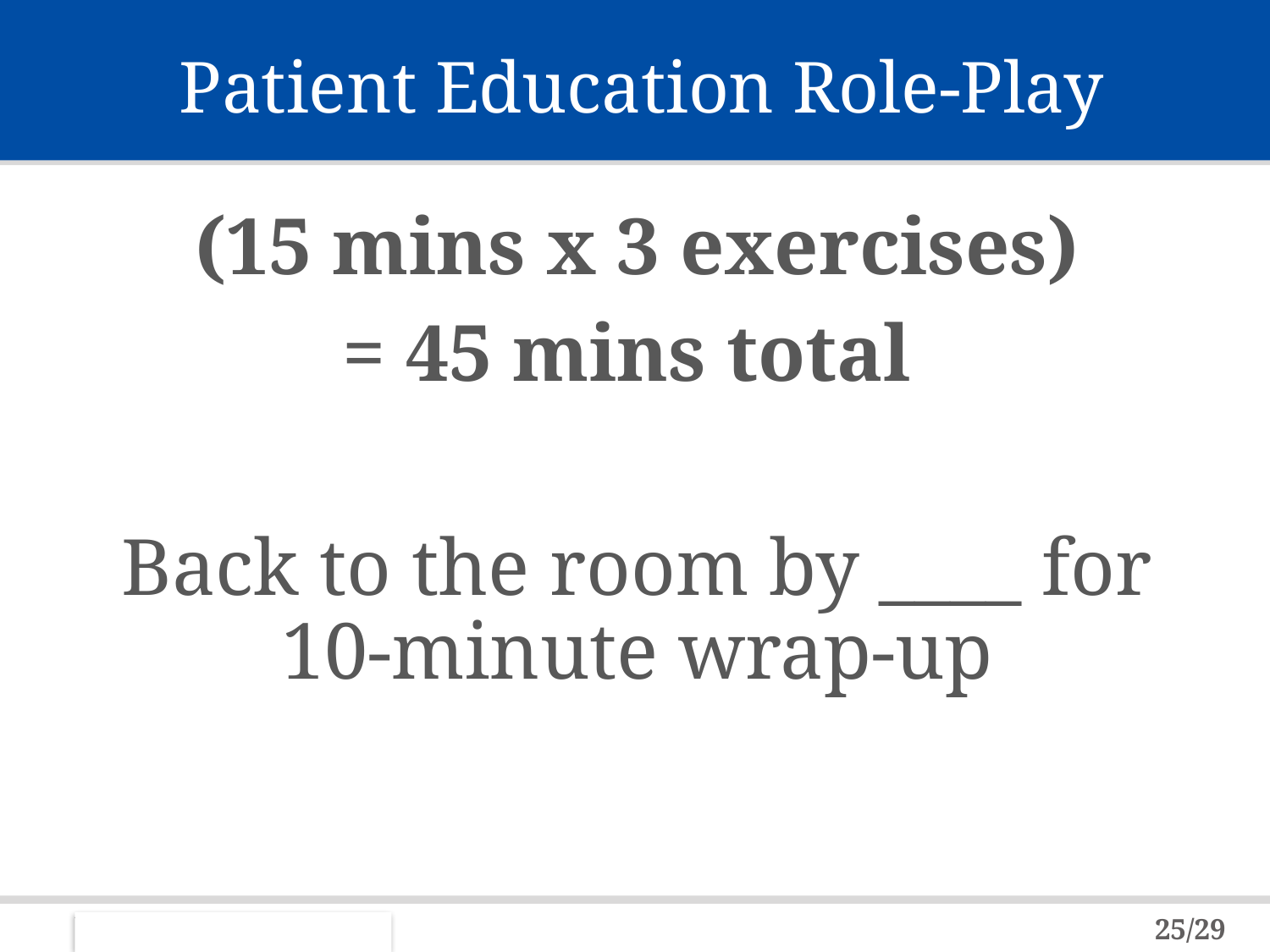

Patient Education Role-Play
(15 mins x 3 exercises)
= 45 mins total
Back to the room by ____ for 10-minute wrap-up
25/29

## Slide 27
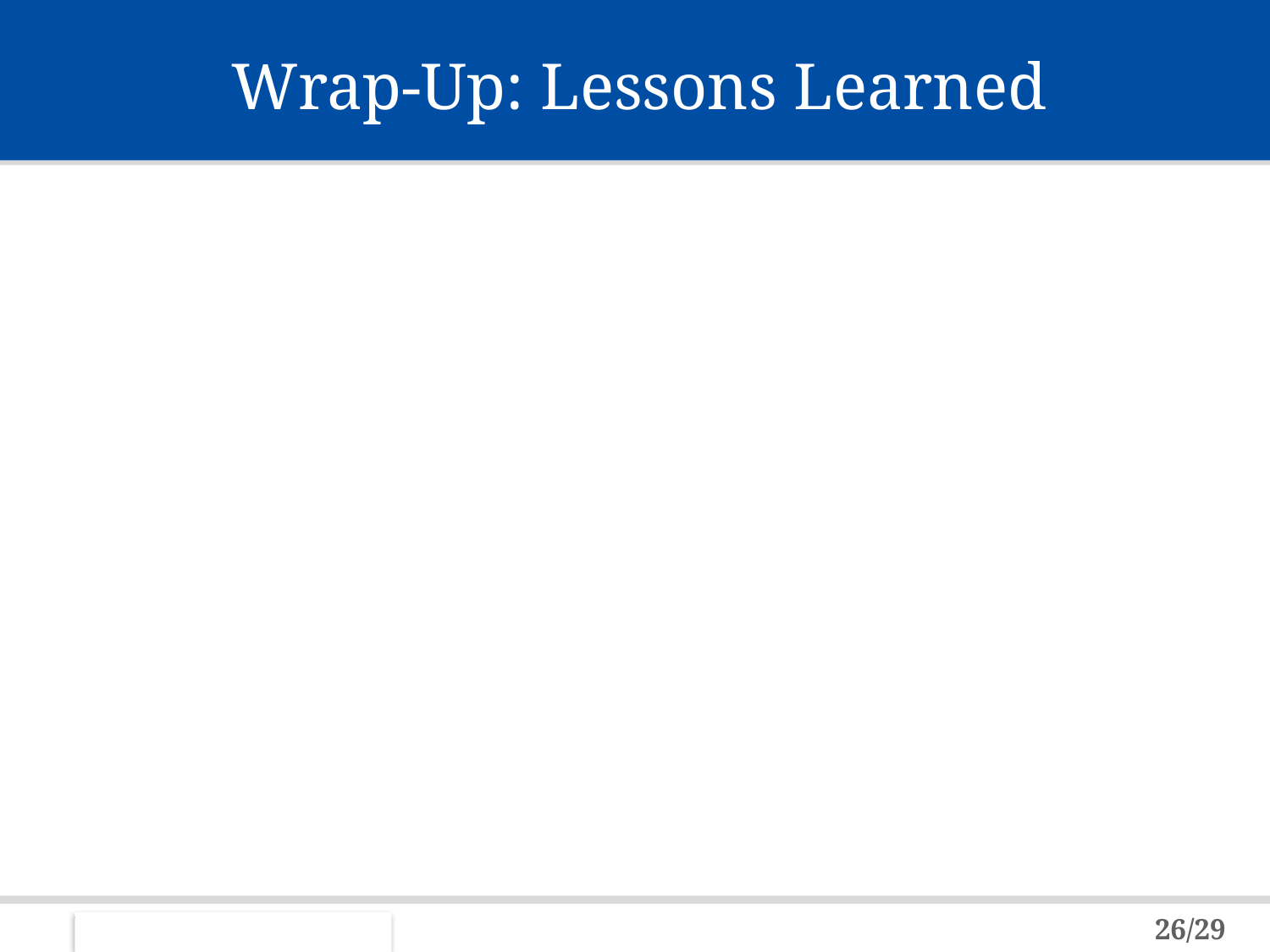

# Wrap-Up: Lessons Learned
26/29

## Slide 28
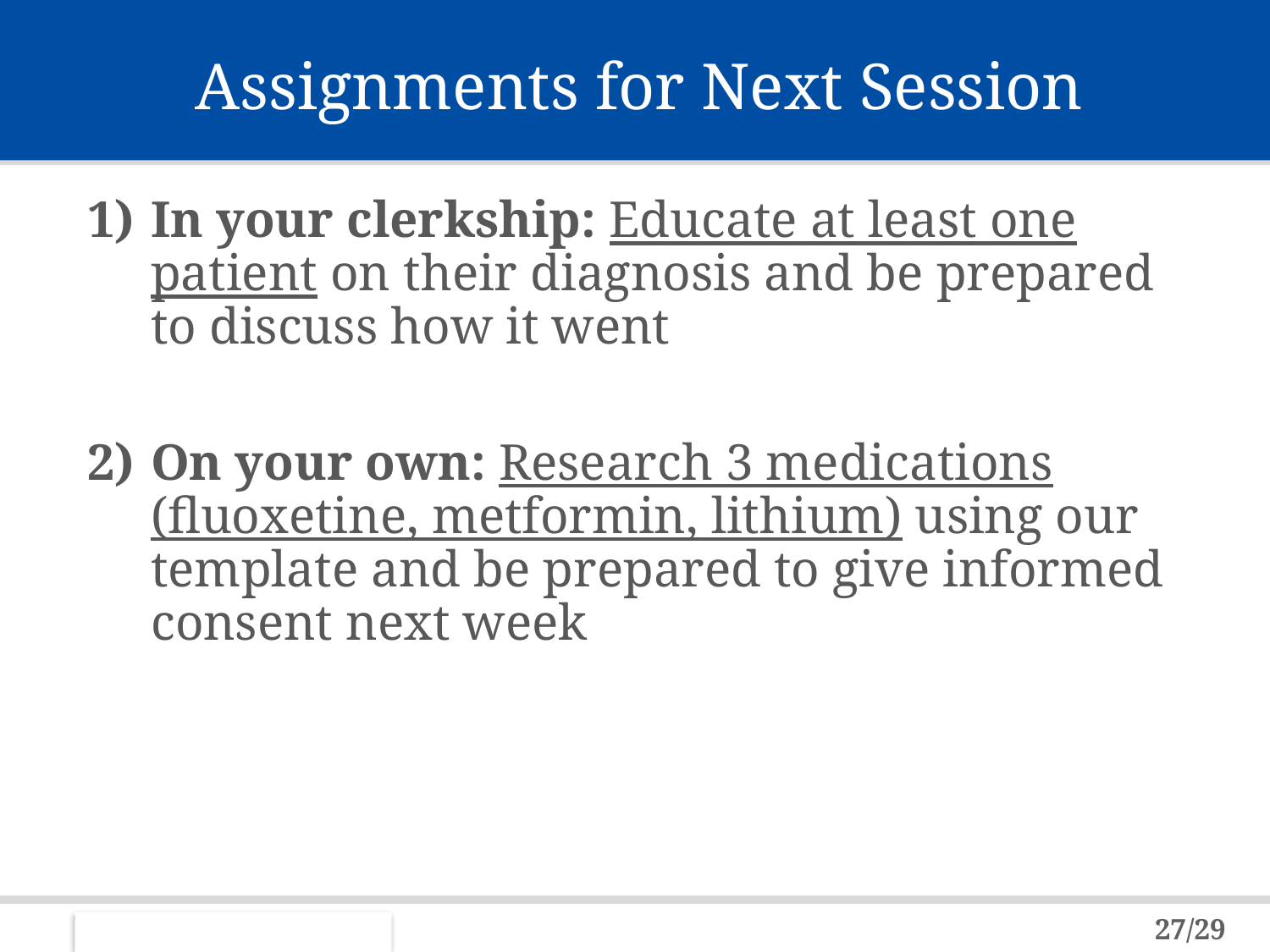

# Assignments for Next Session
In your clerkship: Educate at least one patient on their diagnosis and be prepared to discuss how it went
On your own: Research 3 medications (fluoxetine, metformin, lithium) using our template and be prepared to give informed consent next week
27/29

## Slide 29
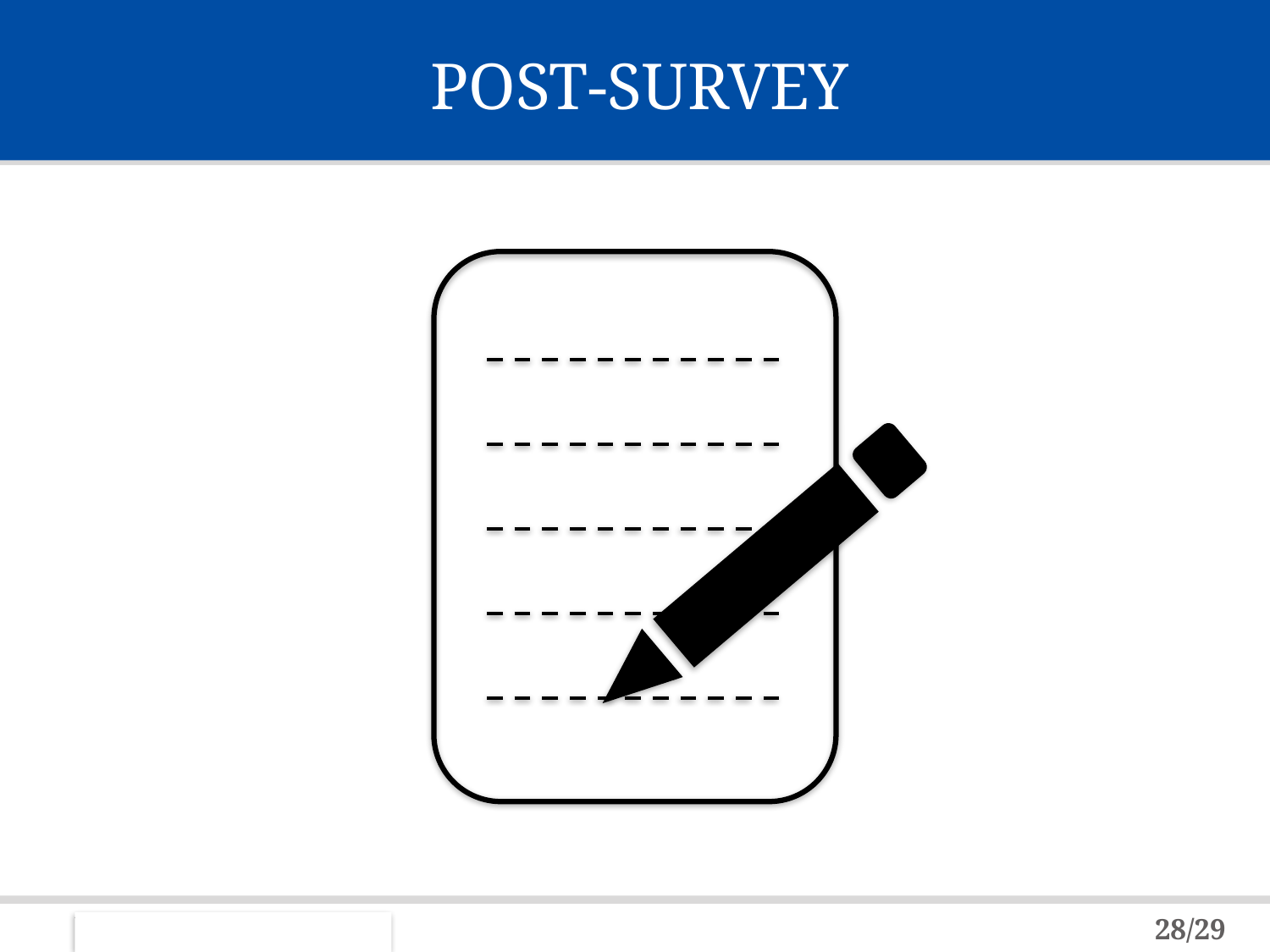

# POST-SURVEY
28/29

## Slide 30
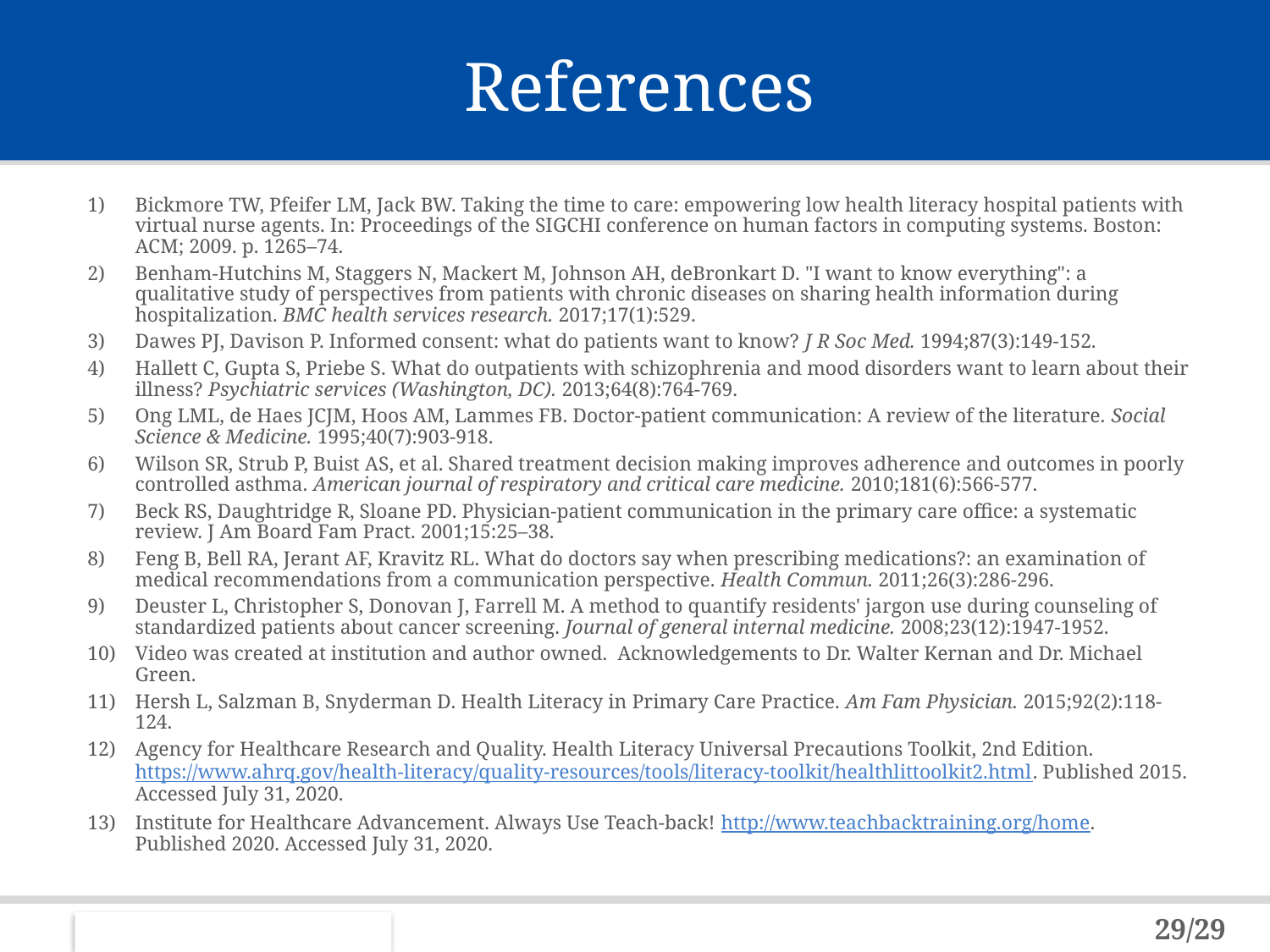

# References
Bickmore TW, Pfeifer LM, Jack BW. Taking the time to care: empowering low health literacy hospital patients with virtual nurse agents. In: Proceedings of the SIGCHI conference on human factors in computing systems. Boston: ACM; 2009. p. 1265–74.
Benham-Hutchins M, Staggers N, Mackert M, Johnson AH, deBronkart D. "I want to know everything": a qualitative study of perspectives from patients with chronic diseases on sharing health information during hospitalization. BMC health services research. 2017;17(1):529.
Dawes PJ, Davison P. Informed consent: what do patients want to know? J R Soc Med. 1994;87(3):149-152.
Hallett C, Gupta S, Priebe S. What do outpatients with schizophrenia and mood disorders want to learn about their illness? Psychiatric services (Washington, DC). 2013;64(8):764-769.
Ong LML, de Haes JCJM, Hoos AM, Lammes FB. Doctor-patient communication: A review of the literature. Social Science & Medicine. 1995;40(7):903-918.
Wilson SR, Strub P, Buist AS, et al. Shared treatment decision making improves adherence and outcomes in poorly controlled asthma. American journal of respiratory and critical care medicine. 2010;181(6):566-577.
Beck RS, Daughtridge R, Sloane PD. Physician-patient communication in the primary care office: a systematic review. J Am Board Fam Pract. 2001;15:25–38.
Feng B, Bell RA, Jerant AF, Kravitz RL. What do doctors say when prescribing medications?: an examination of medical recommendations from a communication perspective. Health Commun. 2011;26(3):286-296.
Deuster L, Christopher S, Donovan J, Farrell M. A method to quantify residents' jargon use during counseling of standardized patients about cancer screening. Journal of general internal medicine. 2008;23(12):1947-1952.
Video was created at institution and author owned. Acknowledgements to Dr. Walter Kernan and Dr. Michael Green.
Hersh L, Salzman B, Snyderman D. Health Literacy in Primary Care Practice. Am Fam Physician. 2015;92(2):118-124.
Agency for Healthcare Research and Quality. Health Literacy Universal Precautions Toolkit, 2nd Edition. https://www.ahrq.gov/health-literacy/quality-resources/tools/literacy-toolkit/healthlittoolkit2.html. Published 2015. Accessed July 31, 2020.
Institute for Healthcare Advancement. Always Use Teach-back! http://www.teachbacktraining.org/home. Published 2020. Accessed July 31, 2020.
29/29
