# Supplementary material for: When Doctor Means Teacher: An Interactive Workshop on Patient-Centered Education
Source: MedEdPORTAL. 2020 Dec 10;16:11053. doi: 10.15766/mep_2374-8265.11053 (PMC7732137; doi:10.15766/mep_2374-8265.11053)
Supplement: Supplementary file 1 — Facilitator Guide.docxPresurvey.docxSession 1 Patient Education Diagnoses.pptxVideo.mp4Session 1 Role-Play Scenarios.docxSession 1 Postsurvey.docxMedication Research Worksheet.docxSession 2 Patient Education Medications.pptxSession 2 Role-Play Scenarios.docxSession 2 Postsurvey.docx [file mep_2374-8265.11053-s001.zip › E. Session 1 Role-Play Scenarios.docx]

**SESSION 1 – PATIENT EDUCATION**

**DIAGNOSIS/PROGNOSIS ROLE PLAY MATERIALS**

**EXERCISE #1 – HYPERTENSION**

**Diagnosis/Prognosis Role Play #1 – Hypertension – DOCTOR**

**YOU** are a family practice doctor at a busy community clinic.

**YOUR PATIENT** recently initiated care with you one month ago and is coming back for a follow-up due to elevated blood pressure at the initial intake appointment. Today’s blood pressure readings are 153/98 and 157/99, which suggests the reading at the last appointment wasn’t an erroneous or unusual value.

- - Demographics: 25yo, married, 2 young children, completed 10^th^ grade, employed as a cashier at a grocery store
  - Medical History: Sciatica
  - Family History: HTN (almost everyone), and CAD (mother, aunt); mother died of a heart attack at age 52
  - Substance Use: Tobacco – 2 packs per day; Social EtOH; no illicit drugs
  - Medications: None
  - Additional Information: You received an e-communication from the patient’s optometrist between visits suggesting that the patient has early signs of hypertensive retinopathy.

**YOUR TASK** is to educate your patient on **hypertension** including the cause, clinical features/symptoms, brief mention of treatment, and prognosis with and without treatment. Please allow between 7-8 minutes, including time for your patient to be involved in the discussion and ask questions.

**CONSIDER** the following:

- - **What does your patient understand** of information you are sharing with them? It may be useful to use simple words/phrases, avoid giving too much information at once, and assess for their understanding.
  - **What questions might you want answered** if you were in your patient’s shoes? It may be useful to anticipate these questions.
  - **What emotional response or barriers** may they have to receiving this news? It may be useful to explore your patient’s worries and concerns regarding the information you share.

**Diagnosis/Prognosis Role Play #1 – Hypertension – PATIENT**

**YOU** are a patient going to a follow-up appointment with your PCP. Last month was your first visit with this doctor to establish care and it was noted that your blood pressure was elevated. You were asked to come back today to re-check your blood pressure and discuss the results with the doctor.

- - Demographics: 25yo, married, 2 young children, completed 10^th^ grade, employed as a cashier at a grocery store
  - Medical History: Sciatica
  - Family History: HTN (almost everyone), and CAD (mother, aunt); mother died of a heart attack at age 52
  - Substance Use: Tobacco – 2 packs per day; Social EtOH; no illicit drugs
  - Medications: None

**ADDITIONAL INFORMATION**

- Life is stressful at the moment – your spouse is out of work, your children are always outgrowing their clothes, and you can barely make ends meet with your salary from the grocery store. In the past few months you’ve been smoking more cigarettes in order to cope with the stress. So far in life you have been relatively healthy and think that the high blood pressure at the first appointment was probably just because of all the stress.
- Your mother took lots of medications for her blood pressure and heart, but she still died of a heart attack last year at the young age of 52; you think the medications didn’t seem to work for her and that they might have made her feel worse towards the end of her life.
- Visiting the doctor is nerve-wracking for you – science class was always difficult because you weren’t able to understand the concepts unless they were explained really simply and slowly. In the past doctors have used big words and made no sense, so you are hoping this doctor is different.

**YOUR TASK** is to assume the role of this patient as you meet your doctor who will go over your repeat blood pressure readings and talk with you about his/her recommendations with regards to your health. Please make some of your concerns (above) known to your doctor. Also, be sure to ask questions if you think the patient you are role-playing may have some difficulty understanding.

**Diagnosis/Prognosis Role Play #1 – Hypertension – OBSERVER**

**YOUR ROLES** as observer are to:

1. **Observe** the patient/physician discussion
2. **Keep your group on time (12-15 minutes total)**
   - 2-3 minutes to read scenario and prepare
   - 7-8 minutes for scenario role-play
   - 3-4 minutes for feedback
3. **Critically evaluate** the delivery of patient-centered education (use the checklist below and take notes)
4. **Facilitate** the effective use of feedback after the simulation (use the structure below)

**The Patient-Education Observer Checklist** (The doctor…)

- used non-verbal communication strategies effectively (e.g. eye contact, sitting down)
- established rapport before initiating the medical discussion
- described *the cause* of hypertension (e.g. using pictures, plain language)
- described *the clinical features and symptoms* of hypertension
- described *the prognosis* of hypertension
- described brief *treatment options* for hypertension (“need to know” vs. “nice to know”)
- described *potential consequences of no treatment* for hypertension
- used patient-centered plain language
- avoided overwhelming the patient with information
- assessed for the patient’s understanding (e.g. asking open-ended questions, using teach-back)
- allowed the patient the chance to ask questions
- showed empathy for the patient’s concerns and worries
- engaged the patient in the discussion enabling a shared decision-making process

**The Feedback Process (3-4 minutes total)**

- Ask the patient…
  - How they felt receiving the patient-education
  - To describe one aspect of the education that the doctor performed well
  - To describe one aspect of the education that the doctor could improve
- Ask the doctor…
  - How they felt giving the patient-education
  - To describe one aspect of the education that they performed well
  - To describe one aspect of the education that they could improve
- You (the observer)…
  - Identify one additional aspect of the education that the doctor performed well
  - Identify one additional aspect of the education that the doctor could improve
  - Go through the observer checklist with your group as a way to summarize the overall feedback session

As a group, write ONE lesson learned from this role play that would be worth sharing in the large group wrap-up:

**SESSION 1 – PATIENT EDUCATION**

**DIAGNOSIS/PROGNOSIS ROLE PLAY MATERIALS**

**EXERCISE #2 – DEPRESSION**

**Diagnosis/Prognosis Role Play #2 – Depression – DOCTOR**

**YOU** are a psychiatrist on the inpatient adolescent psychiatric unit who is meeting with your patient’s concerned **parent.**

**YOUR PATIENT** is a 15yo boy who was recently admitted to the inpatient psychiatric unit after admitting to a teacher that he was having suicidal thoughts with a plan to cut his wrists with a knife.

- Initial evaluation suggests he is profoundly depressed – he met 7/9 criteria for major depression and had blunted affect on exam
- Medical History: childhood asthma
- Social History: parents recently divorced, prior A/B student, but now failing multiple classes in 10^th^ grade
- Family History: grandfather who died by suicide
- Substance Use: smokes marijuana alone weekly
- Medications: albuterol PRN
- Psychiatric History: no past hospitalizations, suicide attempts, medications, or outpatient treatment
- Additional Information: at your first meeting, he tells you “my parents don’t believe in mental conditions.”

**YOUR TASK** is to educate the **parent of your patient** on **depression** including the cause, clinical features/symptoms, brief mention of treatment, and prognosis with and without treatment. Please allow between 7-8 minutes, including time for your patient’s parent to be involved in the discussion and ask questions.

**CONSIDER** the following:

- - **What does your patient understand** of information you are sharing with them? It may be useful to use simple words/phrases, avoid giving too much information at once, and assess for their understanding.
  - **What questions might you want answered** if you were in your patient’s shoes? It may be useful to anticipate these questions.
  - **What emotional response or barriers** may they have to receiving this news? It may be useful to explore your patient’s worries and concerns regarding the information you share.

**Diagnosis/Prognosis Role Play #2 – Depression – PATIENT**

**YOU** are the concerned parent of a patient who was admitted to the inpatient psychiatric unit after disclosing suicidal thoughts to his teacher. Your son told the teacher he wanted to cut his wrists. The psychiatric team on the unit have called a meeting with you to talk through your son’s case.

- Your son is 15yo and is in 10^th^ grade; he does well in school and has been getting A/Bs (although it has been a few months since his last report card)
- Medical History: childhood asthma
- Substance Use: none (as far as you know)
- Family History: patient’s grandfather (your father) died by suicide
- Medications: albuterol PRN
- Psychiatric History: no past hospitalizations, suicide attempts, medications, or outpatient treatment

**ADDITIONAL INFORMATION**

- Life is stressful at the moment – you recently got divorced and both kids have been living with you. You have to work two jobs to make ends meet and it is possible that the job you are currently missing for this meeting is going to fire you! It is upsetting to you that the doctor couldn’t meet up after your work hours.
- So far in life your son has been relatively healthy and well-adjusted; you think the stress of the divorce was too much for him and you feel guilty.
- Your father (the patient’s grandfather) died by suicide when you were young. The family doesn’t talk about it much because it is a shameful topic, but you feel that it was selfish of him to kill himself and leave your mother to raise you alone.
- The thought of meeting with “a shrink” is scary to you – if they are anything like what you’ve seen on TV, it will be a really awkward conversation and that makes you nervous.

**YOUR TASK** is to assume the role of this patient’s parent as you meet the doctor who will discuss your son’s case and talk with you about his/her recommendations with regards to his health. Please make some of your concerns (above) known to the doctor. Also, be sure to ask questions if you think the parent you are role-playing may have some difficulty understanding.

**Diagnosis/Prognosis Role Play #2 – Depression – OBSERVER**

**YOUR ROLES** as observer are to:

1. **Observe** the patient/physician discussion
2. **Keep your group on time (12-15 minutes total)**
   - 2-3 minutes to read scenario and prepare
   - 7-8 minutes for scenario role-play
   - 3-4 minutes for feedback
3. **Critically evaluate** the delivery of patient-centered education (use the checklist below and take notes)
4. **Facilitate** the effective use of feedback after the simulation (use the structure below)

**The Patient-Education Observer Checklist** (The doctor…)

- used non-verbal communication strategies effectively (e.g. eye contact, sitting down)
- established rapport before initiating the medical discussion
- described *the cause* of depression (e.g. using pictures, plain language)
- described *the clinical features and symptoms* of depression
- described *the prognosis* of depression
- described brief *treatment options* for depression (“need to know” vs. “nice to know”)
- described *potential consequences of no treatment* for depression
- used patient-centered plain language
- avoided overwhelming the patient with information
- assessed for the patient’s understanding (e.g. asking open-ended questions, using teach-back)
- allowed the patient the chance to ask questions
- showed empathy for the patient’s concerns and worries
- engaged the patient in the discussion enabling a shared decision-making process

**The Feedback Process (3-4 minutes total)**

- Ask the patient…
  - How they felt receiving the patient-education
  - To describe one aspect of the education that the doctor performed well
  - To describe one aspect of the education that the doctor could improve
- Ask the doctor…
  - How they felt giving the patient-education
  - To describe one aspect of the education that they performed well
  - To describe one aspect of the education that they could improve
- You (the observer)…
  - Identify one additional aspect of the education that the doctor performed well
  - Identify one additional aspect of the education that the doctor could improve
  - Go through the observer checklist with your group as a way to summarize the overall feedback session

As a group, write ONE lesson learned from this role play that would be worth sharing in the large group wrap-up:

**SESSION 1 – PATIENT EDUCATION**

**DIAGNOSIS/PROGNOSIS ROLE PLAY MATERIALS**

**EXERCISE #3 – DIABETES**

**Diagnosis/Prognosis Role Play #3 – Diabetes – DOCTOR**

**YOU** are an endocrinologist at a private multi-specialty group office.

**YOUR PATIENT** was referred to you from a local primary physician after multiple elevated fasting glucose values (>200mg/dL). Prior to meeting with you, you had the patient’s hemoglobin A1c level checked and it came back at 8.2%.

- - Demographics: 38yo, single, working as a dog groomer, living with parents
  - Medical History: Down Syndrome, borderline intellectual disability, depression, ventricular septal defect (surgically repaired), atlantoaxial instability, asthma, hypothyroidism
  - Family History: None significant
  - Substance Use: Occasional minor EtOH use
  - Medications: Levothyroxine, Fluoxetine, Symbicort inhaler, Multivitamin
  - Additional Information: On the initial patient questionnaire, they checked “frequent urination” and “tingling in feet” on the review of systems section

**YOUR TASK** is to educate your patient on **diabetes** including the cause, clinical features/symptoms, brief mention of treatment, and prognosis with and without treatment. Please allow between 7-8 minutes, including time for your patient to be involved in the discussion and ask questions.

**CONSIDER** the following:

- - **What does your patient understand** of information you are sharing with them? It may be useful to use simple words/phrases, avoid giving too much information at once, and assess for their understanding.
  - **What questions might you want answered** if you were in your patient’s shoes? It may be useful to anticipate these questions.
  - **What emotional response or barriers** may they have to receiving this news? It may be useful to explore your patient’s worries and concerns regarding the information you share.

**Diagnosis/Prognosis Role Play #3 – Diabetes – PATIENT**

**YOU** are a patient going to meet with an endocrinologist at a specialty clinic after a referral from your primary doctor. Apparently, some of your sugar readings were high in the last few months and they also did a blood test for diabetes – you have not been told of the results yet.

- - Demographics: 38yo, single, working as a dog groomer, living with parents
  - Medical History: Down Syndrome, borderline intellectual disability, depression, ventricular septal defect (surgically repaired), atlantoaxial instability, asthma, hypothyroidism
  - Family History: None significant
  - Substance Use: Occasional minor EtOH use
  - Medications: Levothyroxine, Fluoxetine, Symbicort inhaler, Multivitamin

**ADDITIONAL INFORMATION**

- Your family are all very healthy, but for some reason you ended up with lots of medical problems and are always being referred to different specialty clinics. You wonder when this will stop and each time you get a new diagnosis it feels very disheartening and seems to send your depression in to a downward spiral.
- On the initial patient questionnaire, you checked “frequent urination” and “tingling in feet” on the review of systems section; these symptoms have been bothering you for the last few months and you didn’t get a chance to tell your primary doctor about them.
- When you were in school people called you “stupid” because you had to take special education classes. Since then, you’ve always been intimated by talking to “smart” people and meeting with this doctor is making you nervous. You hope that the doctor talks slowly and simply so that you can follow and understand.

**YOUR TASK** is to assume the role of this patient as you meet your doctor who will go over your elevated blood sugar readings and recent blood test and talk with you about his/her recommendations with regards to your health. Please make some of your concerns (above) known to your doctor. Also, be sure to ask questions if you think the patient you are role-playing may have some difficulty understanding.

**Diagnosis/Prognosis Role Play #3 – Diabetes – OBSERVER**

**YOUR ROLES** as observer are to:

1. **Observe** the patient/physician discussion
2. **Keep your group on time (12-15 minutes total)**
   - 2-3 minutes to read scenario and prepare
   - 7-8 minutes for scenario role-play
   - 3-4 minutes for feedback
3. **Critically evaluate** the delivery of patient-centered education (use the checklist below and take notes)
4. **Facilitate** the effective use of feedback after the simulation (use the structure below)

**The Patient-Education Observer Checklist** (The doctor…)

- established rapport before initiating the medical discussion
- used non-verbal communication strategies effectively (e.g. eye contact, sitting down)
- established rapport before initiating the medical discussion
- described *the cause* of diabetes (e.g. using pictures, plain language)
- described *the clinical features and symptoms* of diabetes
- described *the prognosis* of diabetes
- described brief *treatment options* for diabetes (“need to know” vs. “nice to know”)
- described *potential consequences of no treatment* for diabetes
- used patient-centered plain language
- avoided overwhelming the patient with information
- assessed for the patient’s understanding (e.g. asking open-ended questions, using teach-back)
- allowed the patient the chance to ask questions
- showed empathy for the patient’s concerns and worries
- engaged the patient in the discussion enabling a shared decision-making process

**The Feedback Process (3-4 minutes total)**

- Ask the patient…
  - How they felt receiving the patient-education
  - To describe one aspect of the education that the doctor performed well
  - To describe one aspect of the education that the doctor could improve
- Ask the doctor…
  - How they felt giving the patient-education
  - To describe one aspect of the education that they performed well
  - To describe one aspect of the education that they could improve
- You (the observer)…
  - Identify one additional aspect of the education that the doctor performed well
  - Identify one additional aspect of the education that the doctor could improve
  - Go through the observer checklist with your group as a way to summarize the overall feedback session

As a group, write ONE lesson learned from this role play that would be worth sharing in the large group wrap-up:
