# Supplementary material for: When Doctor Means Teacher: An Interactive Workshop on Patient-Centered Education
Source: MedEdPORTAL. 2020 Dec 10;16:11053. doi: 10.15766/mep_2374-8265.11053 (PMC7732137; doi:10.15766/mep_2374-8265.11053)
Supplement: Supplementary file 1 — Facilitator Guide.docxPresurvey.docxSession 1 Patient Education Diagnoses.pptxVideo.mp4Session 1 Role-Play Scenarios.docxSession 1 Postsurvey.docxMedication Research Worksheet.docxSession 2 Patient Education Medications.pptxSession 2 Role-Play Scenarios.docxSession 2 Postsurvey.docx [file mep_2374-8265.11053-s001.zip › G. Medication Research Worksheet.docx]

**Patient Education Workshop – Medication Research**

**TASK:** Next session during the patient education workshop, you will be **expected to provide patient-centered education** on a common medication – either fluoxetine, metformin, or lithium. You will receive the exact patient case and task description on the day of the session. **Use the following worksheets to guide your preparatory research** on the medications. You will be expected to convey the following about the medication:

- The name
- The diagnosis/problem and reason you are recommending the medication
- The potential benefit and the likelihood of benefit
- A very basic overview of the mechanism by which the medication works
- Up to 3 common but minor side effects
- Up to 3 rare but major side effects
- Published black box warnings
- Anticipatory guidance for what your patient might do if they encounter any adverse effects
- Alternative options
- Likely course of the illness without using medication
- A brief mention of the duration of use, dosing, and scheduling of usage

When researching this medication and thinking about **how you might convey this to a patient, consider** the following:

- How can you **engage your patient in shared decision-making**? It may be useful to ask for their input and thoughts on what you are sharing with them.
- **What does your patient understand** of the information you are sharing with them? It may be useful to use simple words/phrases, avoid giving too much information at once, and assess for their understanding.
- **What questions might you want answered** if you were in your patient’s shoes? It may be useful to anticipate these questions.
- **What barriers might your patient face** with remaining adherent to this medication? It may be useful to anticipate and explore these barriers.

| **Medication Name** | | Fluoxetine |
| --- | --- | --- |
| **Problem and indication** (what is the problem and why are you recommending this medication?) | |  |
| **Potential likelihood for benefit** (how do you expect it to help?) | |  |
| **Basic mechanism** (in layman-terms, how does it work?) | |  |
| **Risks/side effects with anticipatory guidance** (what should they do if they encounter these side effects?) | **Common minor (up to 3)** | 1)  2)  3) |
|  | **Rare major (up to 3)** | 1)  2)  3) |
|  | **Black box warnings** |  |
| **Alternate treatments** (what are the other options?) | |  |
| **Likely course of illness without treatment** (what happens if they choose not to take it?) | |  |
| **Duration of use** (will they be on this forever?) | |  |
| **Dose and scheduling** (how much and how often?) | |  |

| **Medication Name** | | Metformin |
| --- | --- | --- |
| **Problem and indication** (what is the problem and why are you recommending this medication?) | |  |
| **Potential likelihood for benefit** (how do you expect it to help?) | |  |
| **Basic mechanism** (in layman-terms, how does it work?) | |  |
| **Risks/side effects with anticipatory guidance** (what should they do if they encounter these side effects?) | **Common minor (up to 3)** | 1)  2)  3) |
|  | **Rare major (up to 3)** | 1)  2)  3) |
|  | **Black box warnings** |  |
| **Alternate treatments** (what are the other options?) | |  |
| **Likely course of illness without treatment** (what happens if they choose not to take it?) | |  |
| **Duration of use** (will they be on this forever?) | |  |
| **Dose and scheduling** (how much and how often?) | |  |

| **Medication Name** | | Lithium |
| --- | --- | --- |
| **Problem and indication** (what is the problem and why are you recommending this medication?) | |  |
| **Potential likelihood for benefit** (how do you expect it to help?) | |  |
| **Basic mechanism** (in layman-terms, how does it work?) | |  |
| **Risks/side effects with anticipatory guidance** (what should they do if they encounter these side effects?) | **Common minor (up to 3)** | 1)  2)  3) |
|  | **Rare major (up to 3)** | 1)  2)  3) |
|  | **Black box warnings** |  |
| **Alternate treatments** (what are the other options?) | |  |
| **Likely course of illness without treatment** (what happens if they choose not to take it?) | |  |
| **Duration of use** (will they be on this forever?) | |  |
| **Dose and scheduling** (how much and how often?) | |  |
