# Supplementary material for: When Doctor Means Teacher: An Interactive Workshop on Patient-Centered Education
Source: MedEdPORTAL. 2020 Dec 10;16:11053. doi: 10.15766/mep_2374-8265.11053 (PMC7732137; doi:10.15766/mep_2374-8265.11053)
Supplement: Supplementary file 1 — Facilitator Guide.docxPresurvey.docxSession 1 Patient Education Diagnoses.pptxVideo.mp4Session 1 Role-Play Scenarios.docxSession 1 Postsurvey.docxMedication Research Worksheet.docxSession 2 Patient Education Medications.pptxSession 2 Role-Play Scenarios.docxSession 2 Postsurvey.docx [file mep_2374-8265.11053-s001.zip › H. Session 2 Patient Education Medications.pptx]

## Slide 1
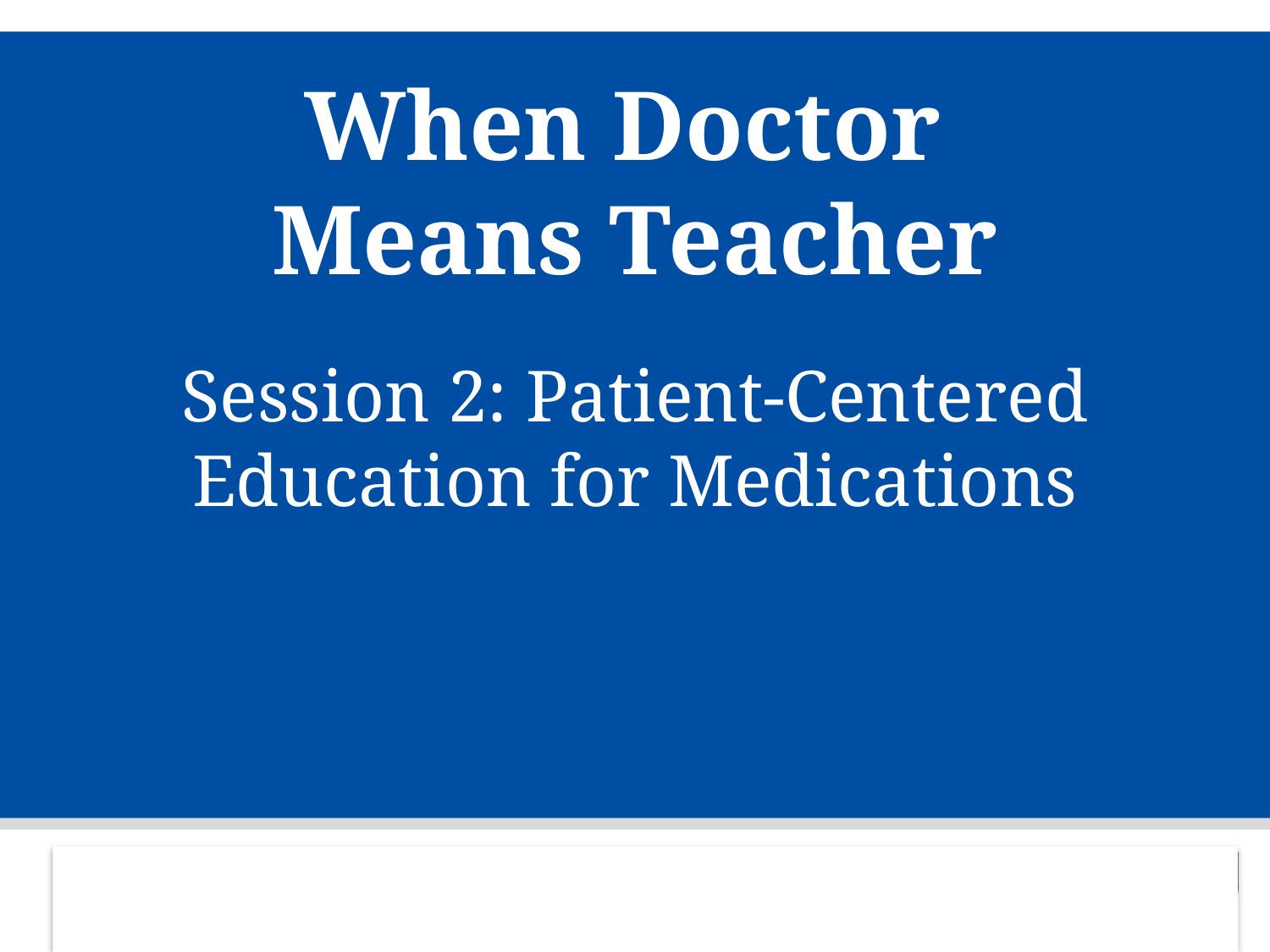

# When Doctor Means Teacher
Session 2: Patient-Centered Education for Medications

## Slide 2
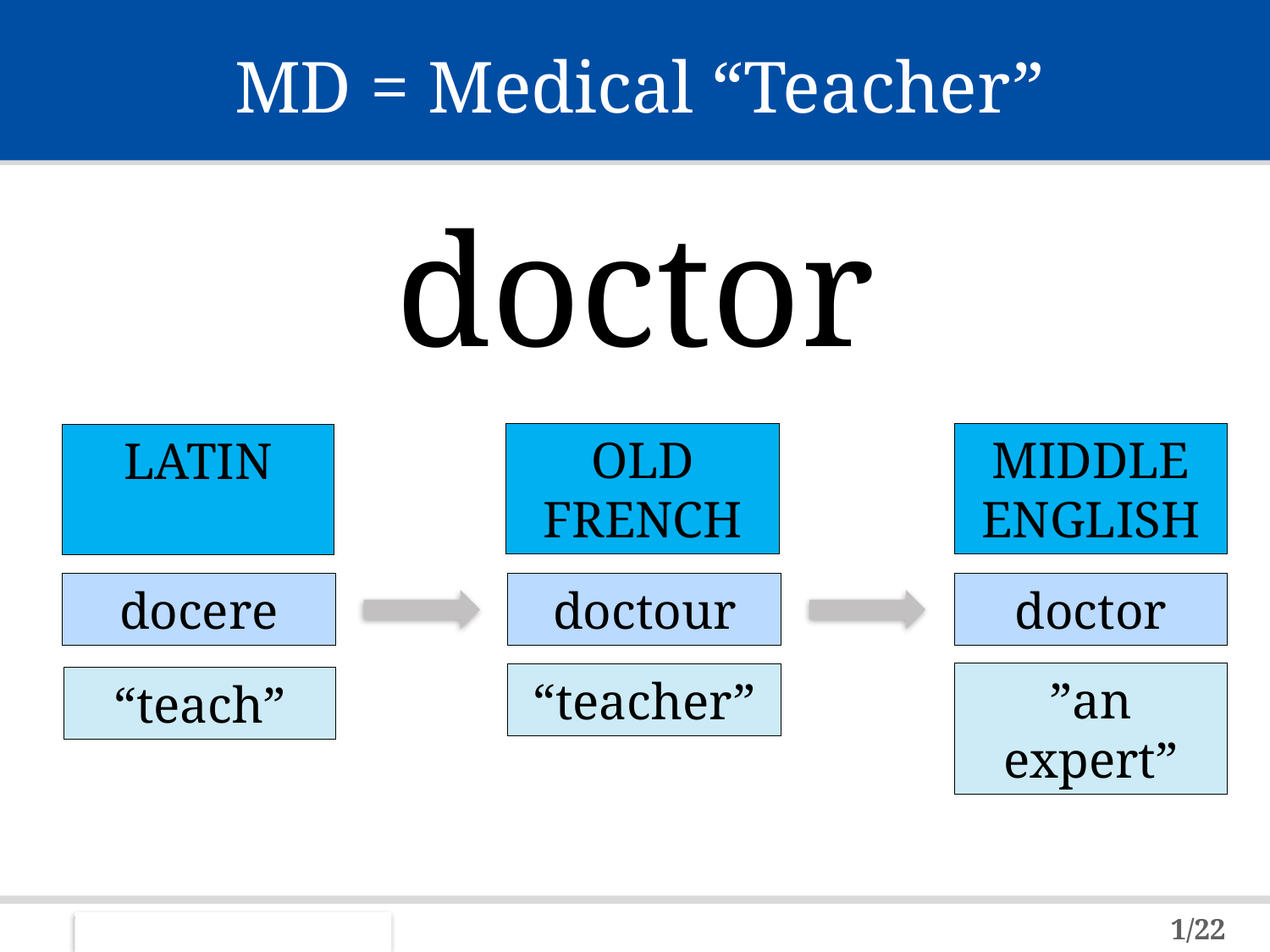

# MD = Medical “Teacher”
MIDDLE ENGLISH
LATIN
OLD FRENCH
docere
doctor
doctour
”an expert”
“teacher”
“teach”
1/22

## Slide 3
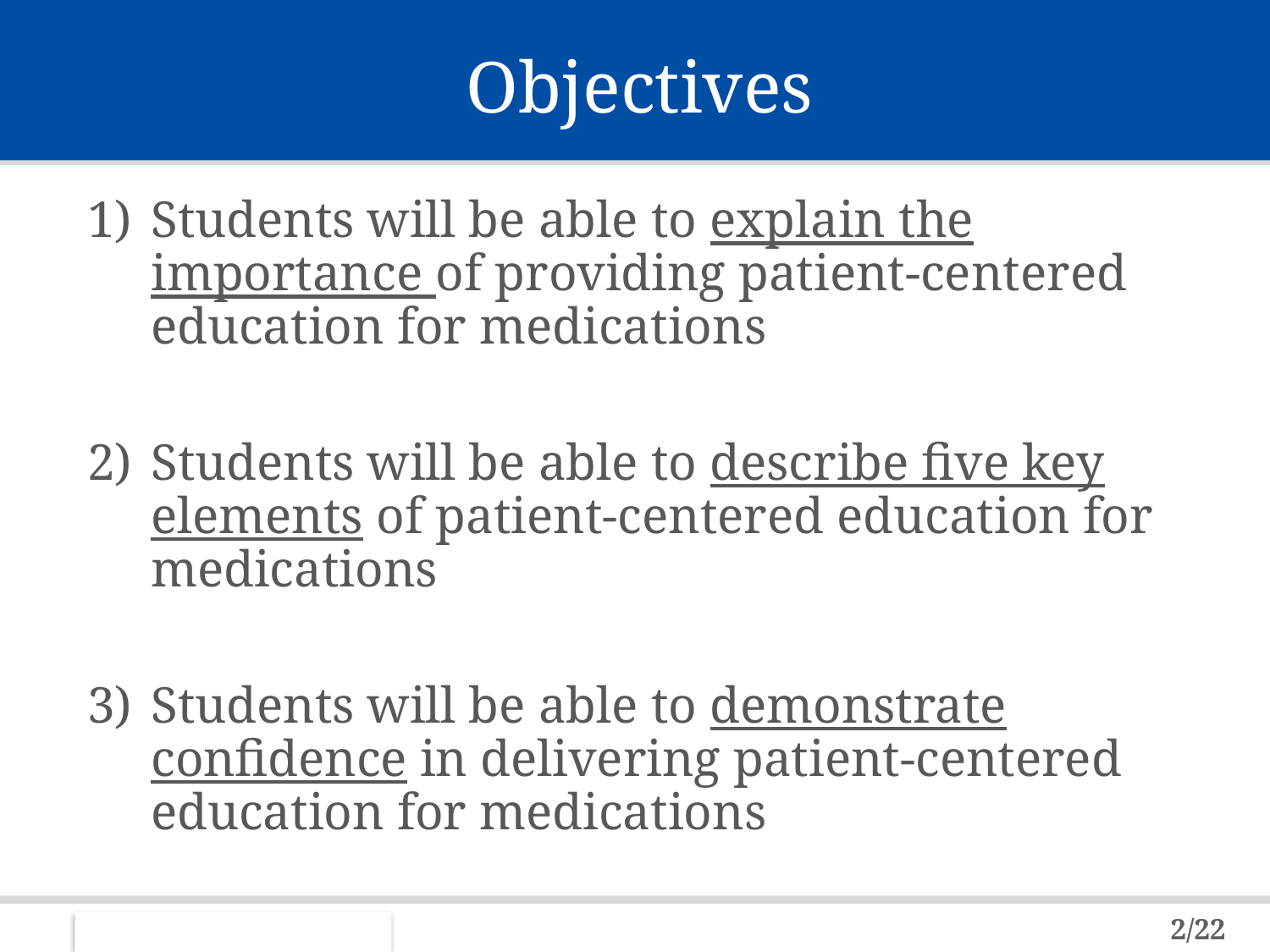

# Objectives
Students will be able to explain the importance of providing patient-centered education for medications
Students will be able to describe five key elements of patient-centered education for medications
Students will be able to demonstrate confidence in delivering patient-centered education for medications
2/22

## Slide 4
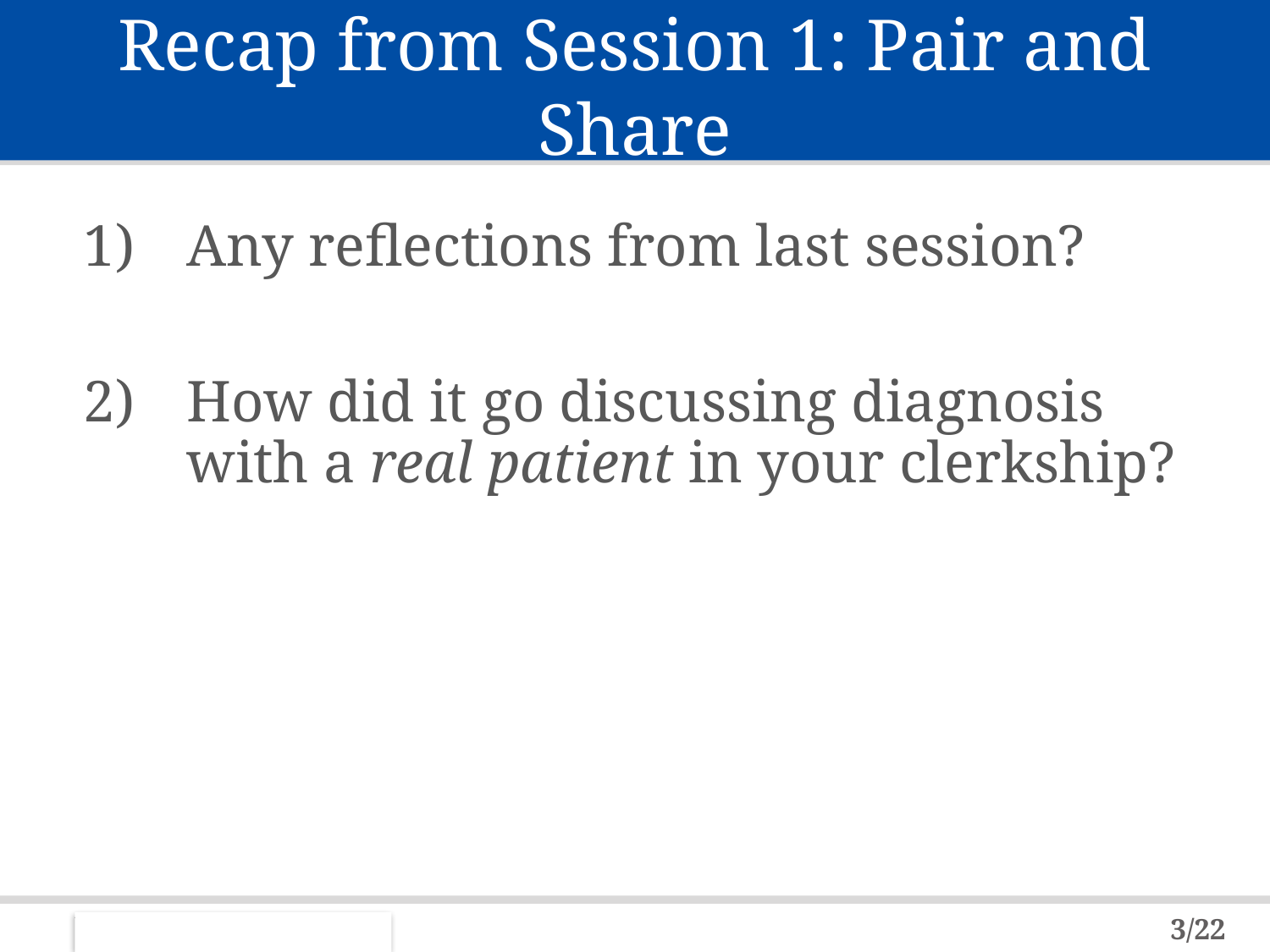

# Recap from Session 1: Pair and Share
Any reflections from last session?
How did it go discussing diagnosis with a real patient in your clerkship?
3/22

## Slide 5
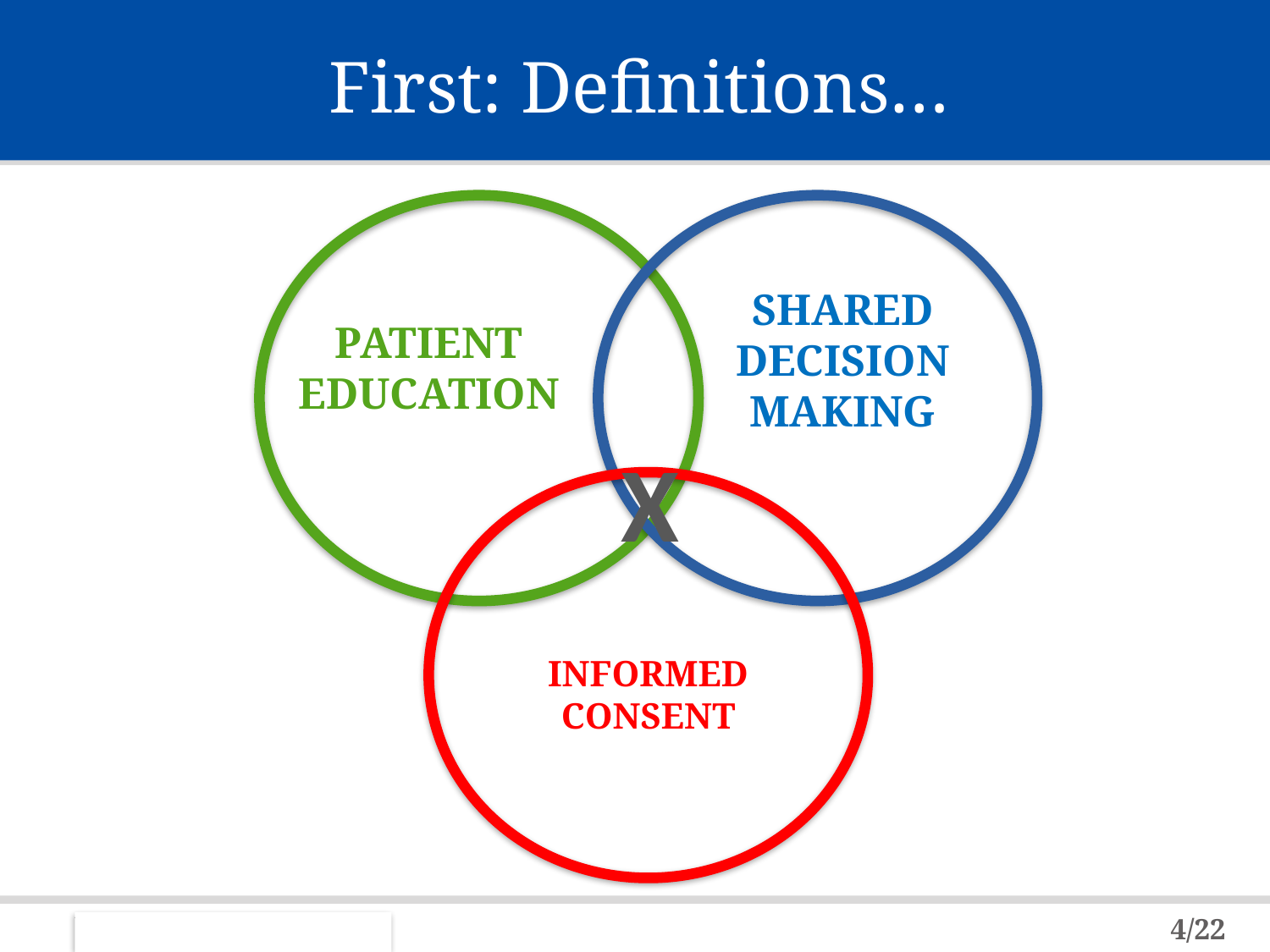

# First: Definitions…
SHARED
DECISION
MAKING
PATIENT
EDUCATION
X
INFORMED
CONSENT
4/22

## Slide 6
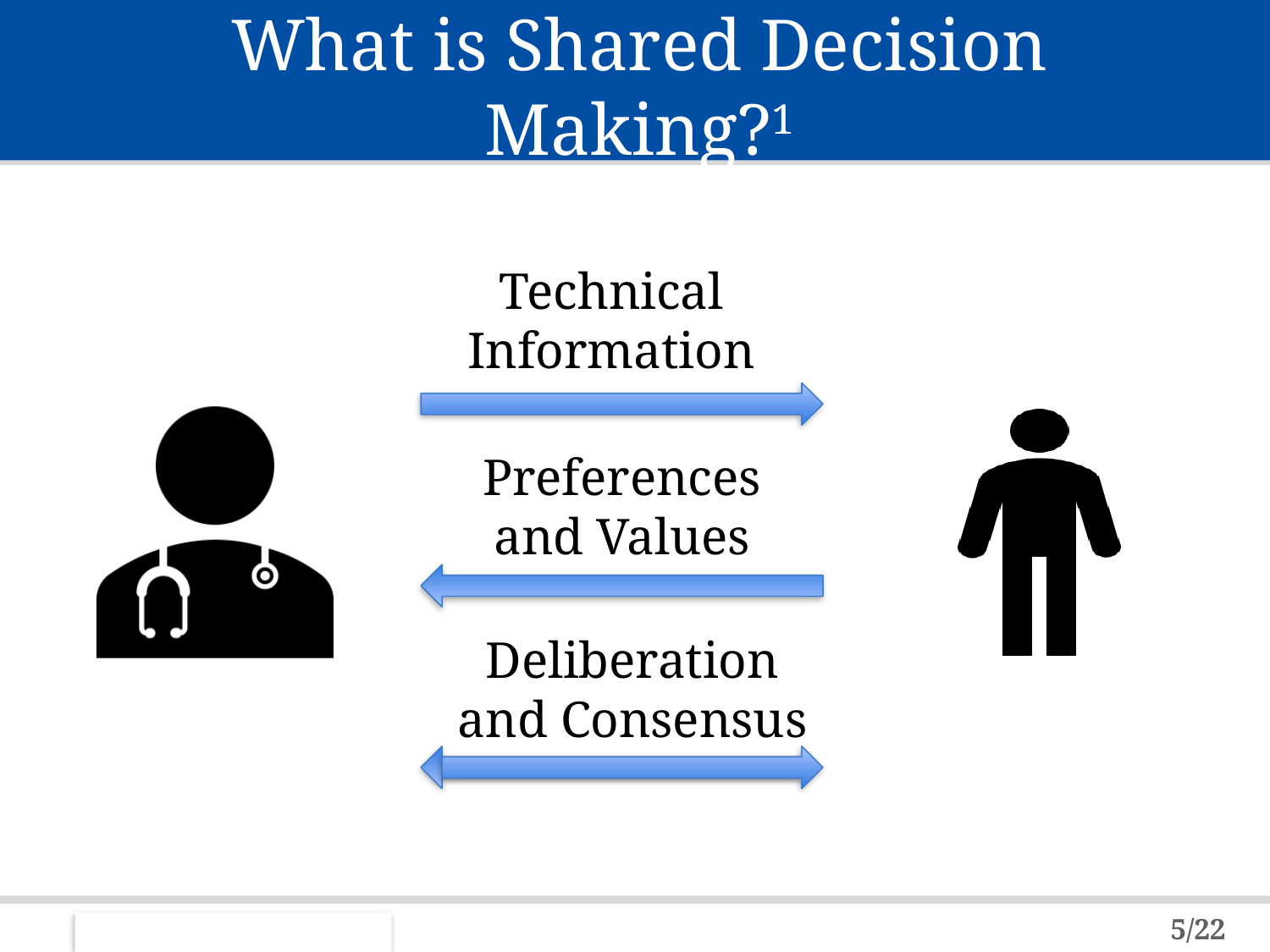

# What is Shared Decision Making?1
Technical Information
Preferences and Values
Deliberation and Consensus
5/22

## Slide 7
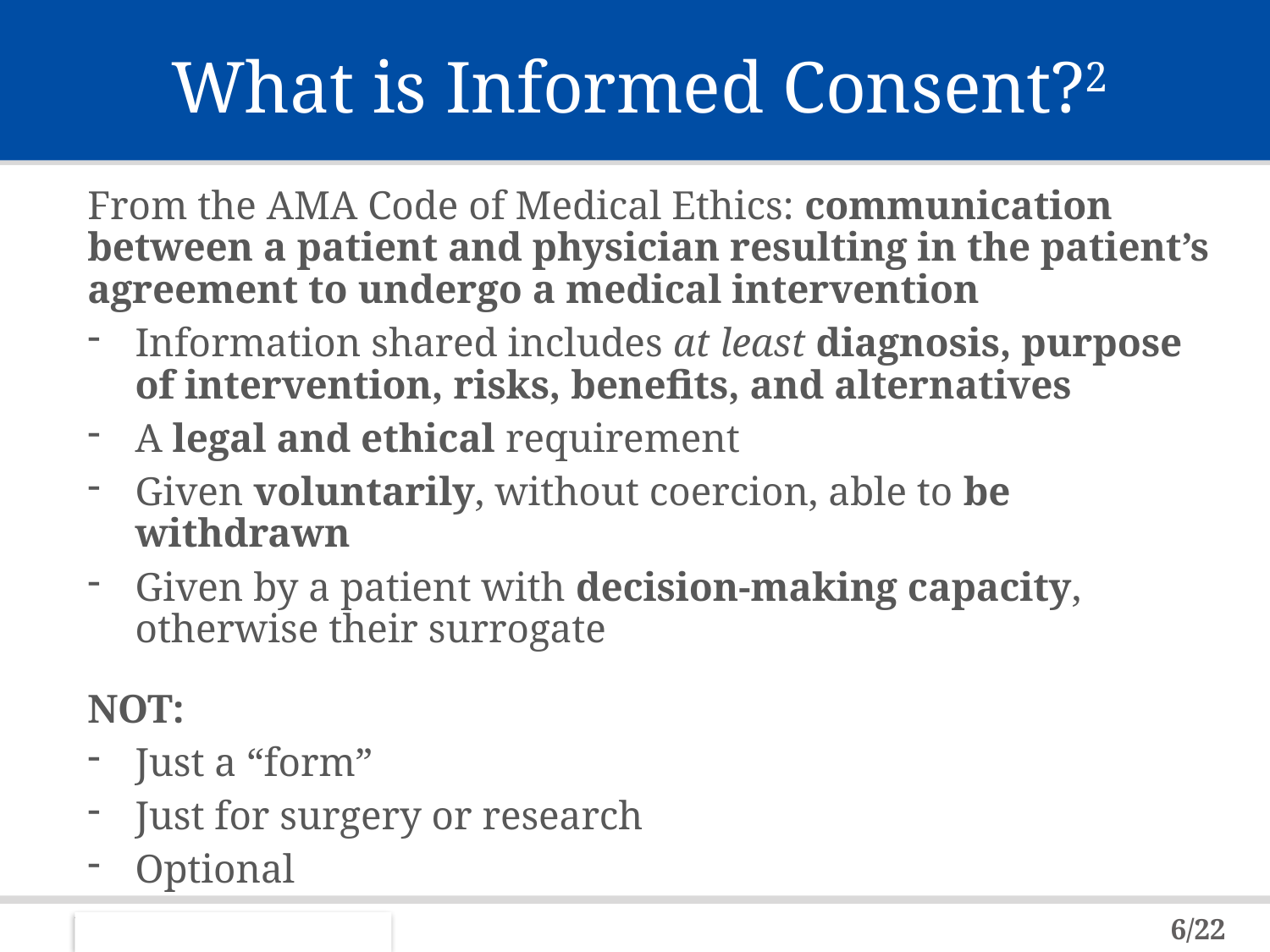

# What is Informed Consent?2
From the AMA Code of Medical Ethics: communication between a patient and physician resulting in the patient’s agreement to undergo a medical intervention
Information shared includes at least diagnosis, purpose of intervention, risks, benefits, and alternatives
A legal and ethical requirement
Given voluntarily, without coercion, able to be withdrawn
Given by a patient with decision-making capacity, otherwise their surrogate
NOT:
Just a “form”
Just for surgery or research
Optional
6/22

## Slide 8
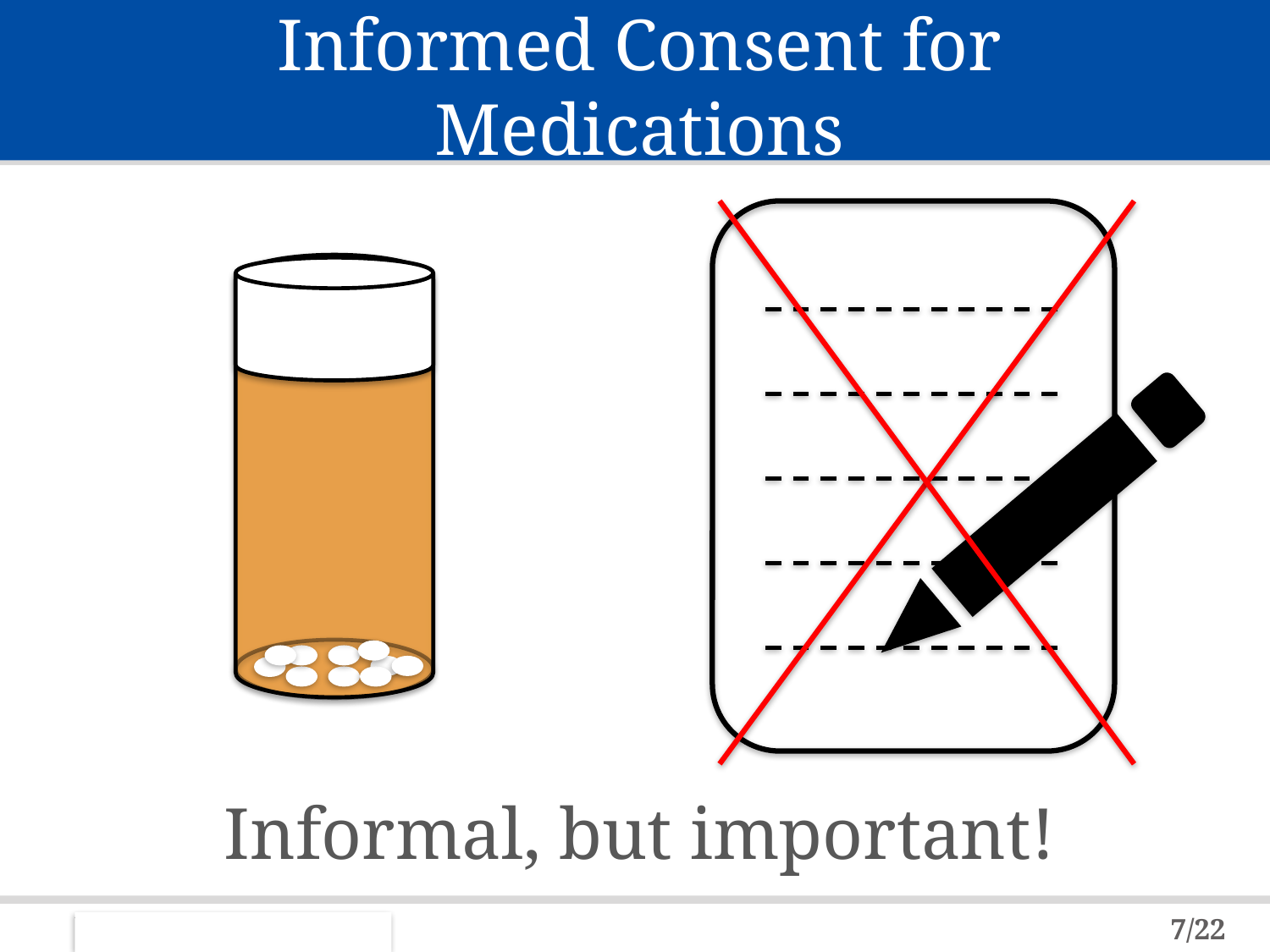

# Informed Consent for Medications
Informal, but important!
7/22

## Slide 9
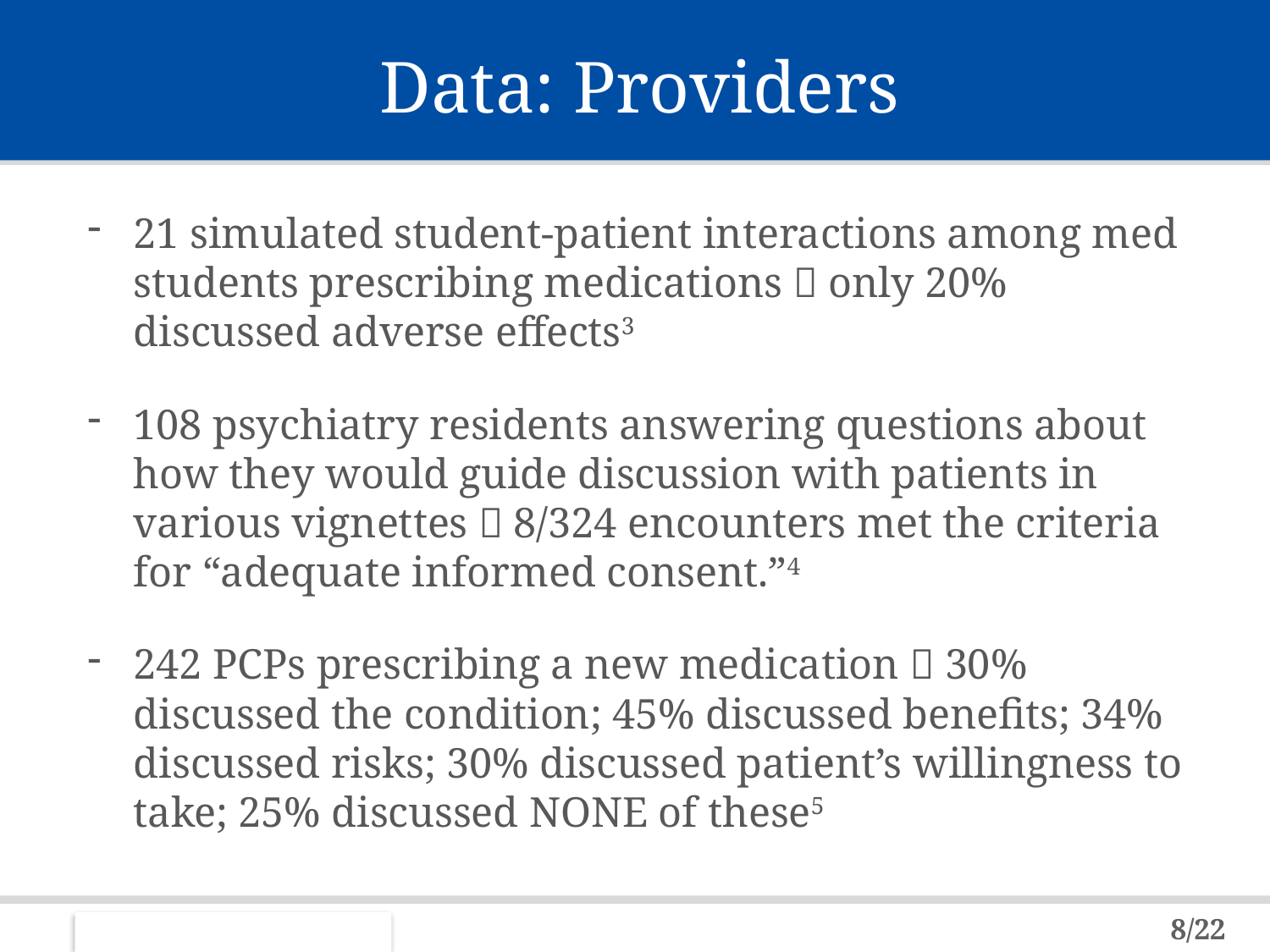

# Data: Providers
21 simulated student-patient interactions among med students prescribing medications  only 20% discussed adverse effects3
108 psychiatry residents answering questions about how they would guide discussion with patients in various vignettes  8/324 encounters met the criteria for “adequate informed consent.”4
242 PCPs prescribing a new medication  30% discussed the condition; 45% discussed benefits; 34% discussed risks; 30% discussed patient’s willingness to take; 25% discussed NONE of these5
8/22

## Slide 10
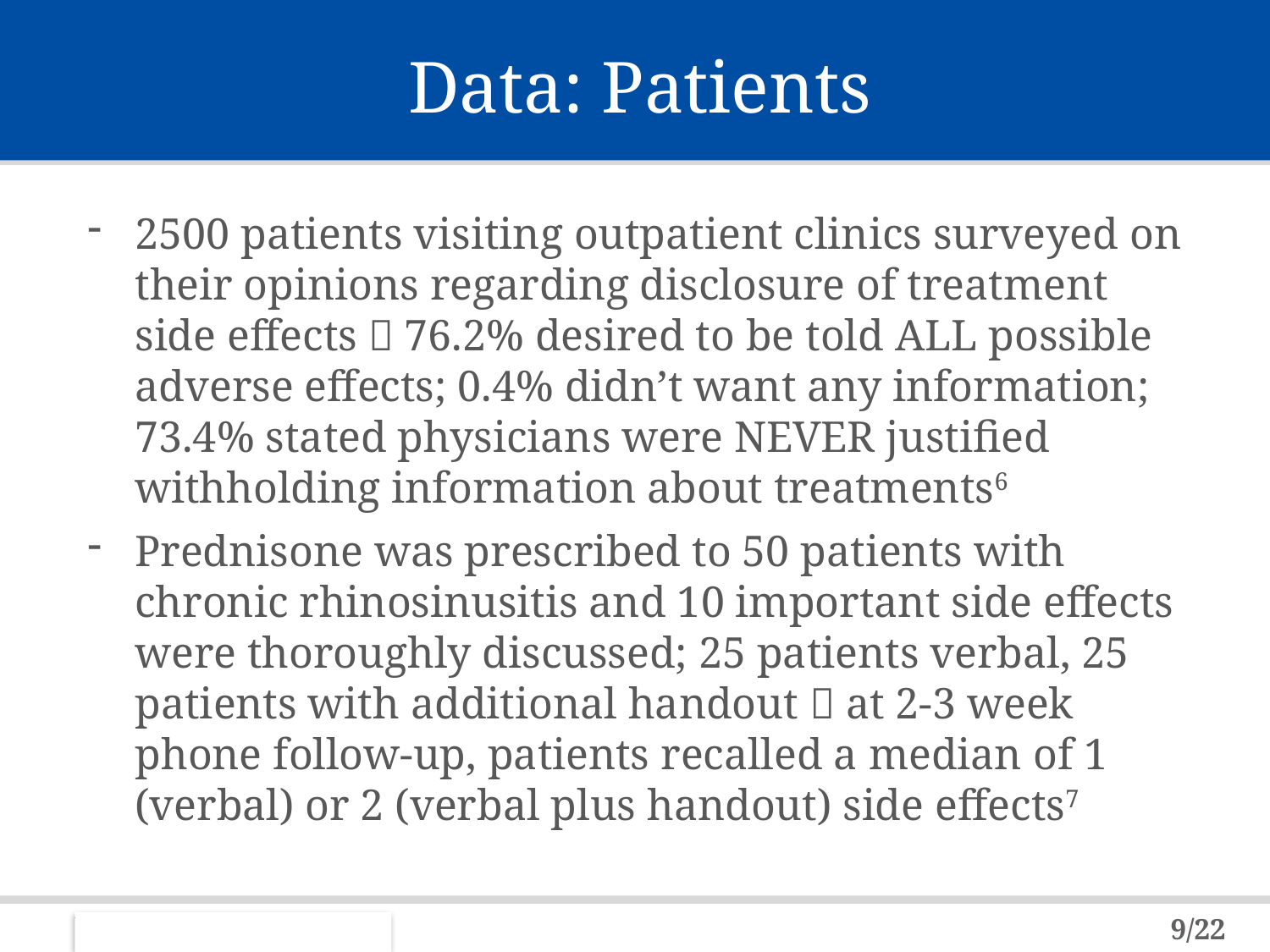

# Data: Patients
2500 patients visiting outpatient clinics surveyed on their opinions regarding disclosure of treatment side effects  76.2% desired to be told ALL possible adverse effects; 0.4% didn’t want any information; 73.4% stated physicians were NEVER justified withholding information about treatments6
Prednisone was prescribed to 50 patients with chronic rhinosinusitis and 10 important side effects were thoroughly discussed; 25 patients verbal, 25 patients with additional handout  at 2-3 week phone follow-up, patients recalled a median of 1 (verbal) or 2 (verbal plus handout) side effects7
9/22

## Slide 11
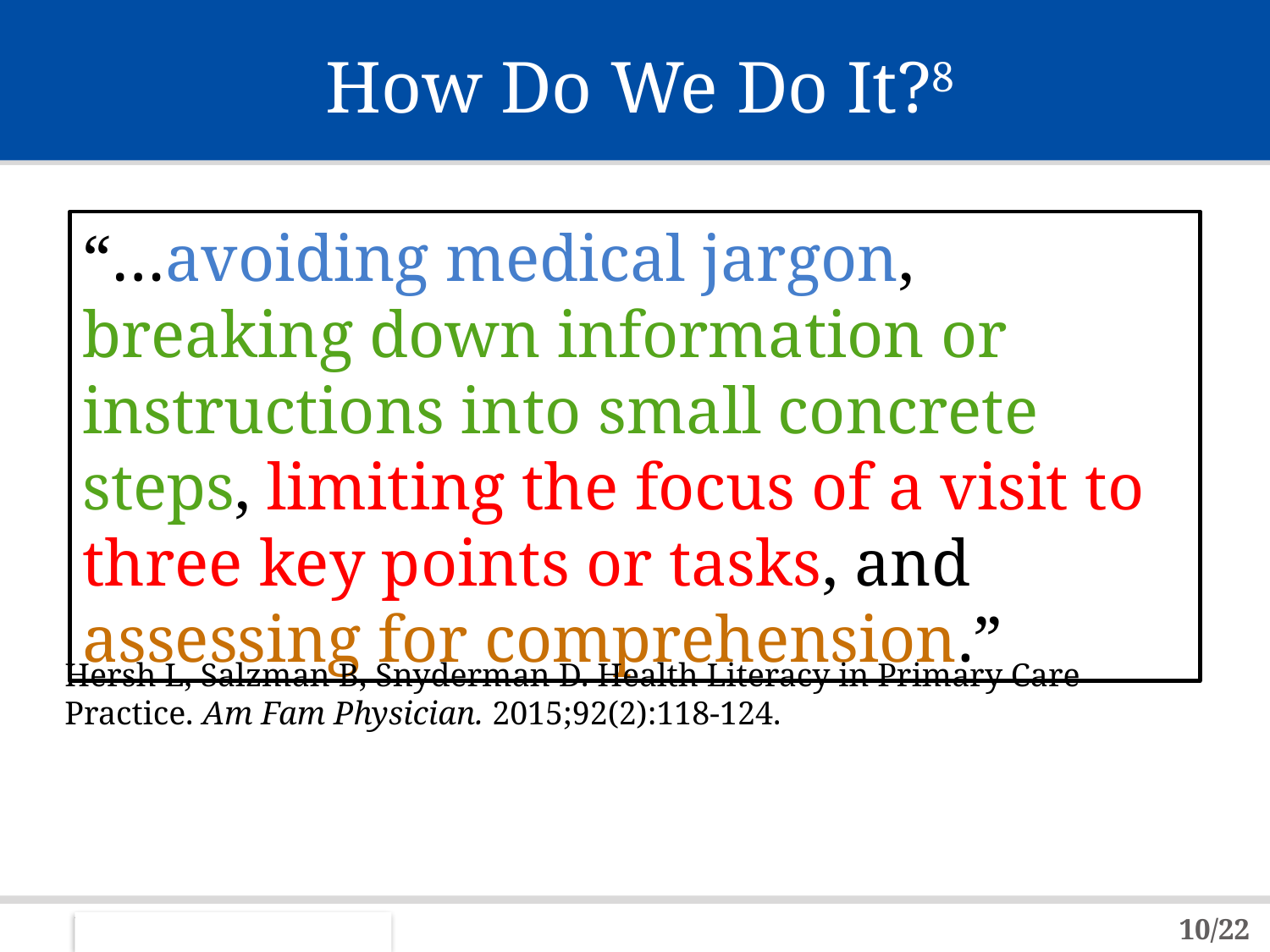

# How Do We Do It?8
“…avoiding medical jargon, breaking down information or instructions into small concrete steps, limiting the focus of a visit to three key points or tasks, and assessing for comprehension.”
Hersh L, Salzman B, Snyderman D. Health Literacy in Primary Care Practice. Am Fam Physician. 2015;92(2):118-124.
10/22

## Slide 12
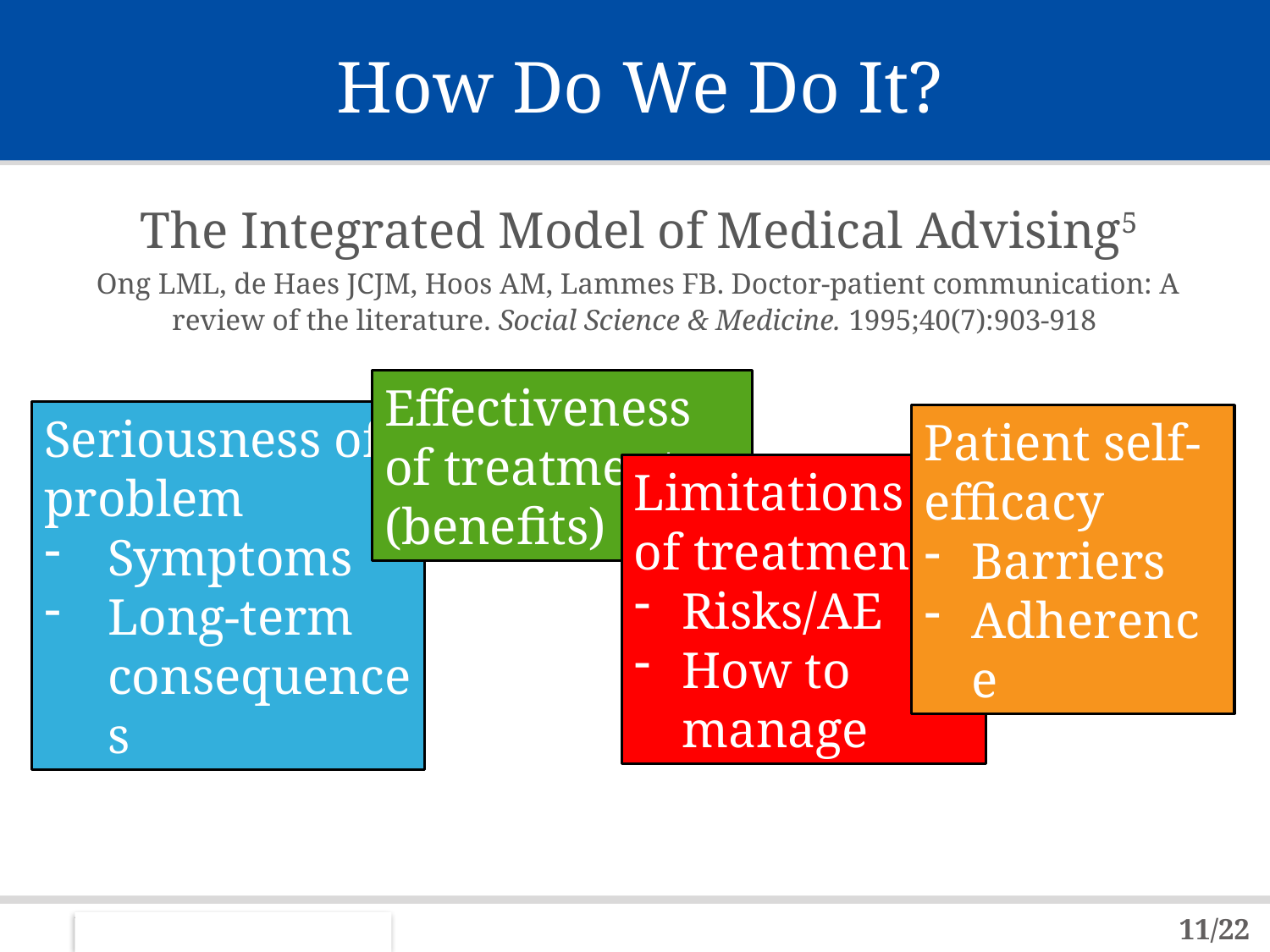

# How Do We Do It?
The Integrated Model of Medical Advising5
Ong LML, de Haes JCJM, Hoos AM, Lammes FB. Doctor-patient communication: A review of the literature. Social Science & Medicine. 1995;40(7):903-918
Effectiveness of treatment (benefits)
Seriousness of problem
Symptoms
Long-term consequences
Patient self-efficacy
Barriers
Adherence
Limitations
of treatment
Risks/AE
How to manage
11/22

## Slide 13
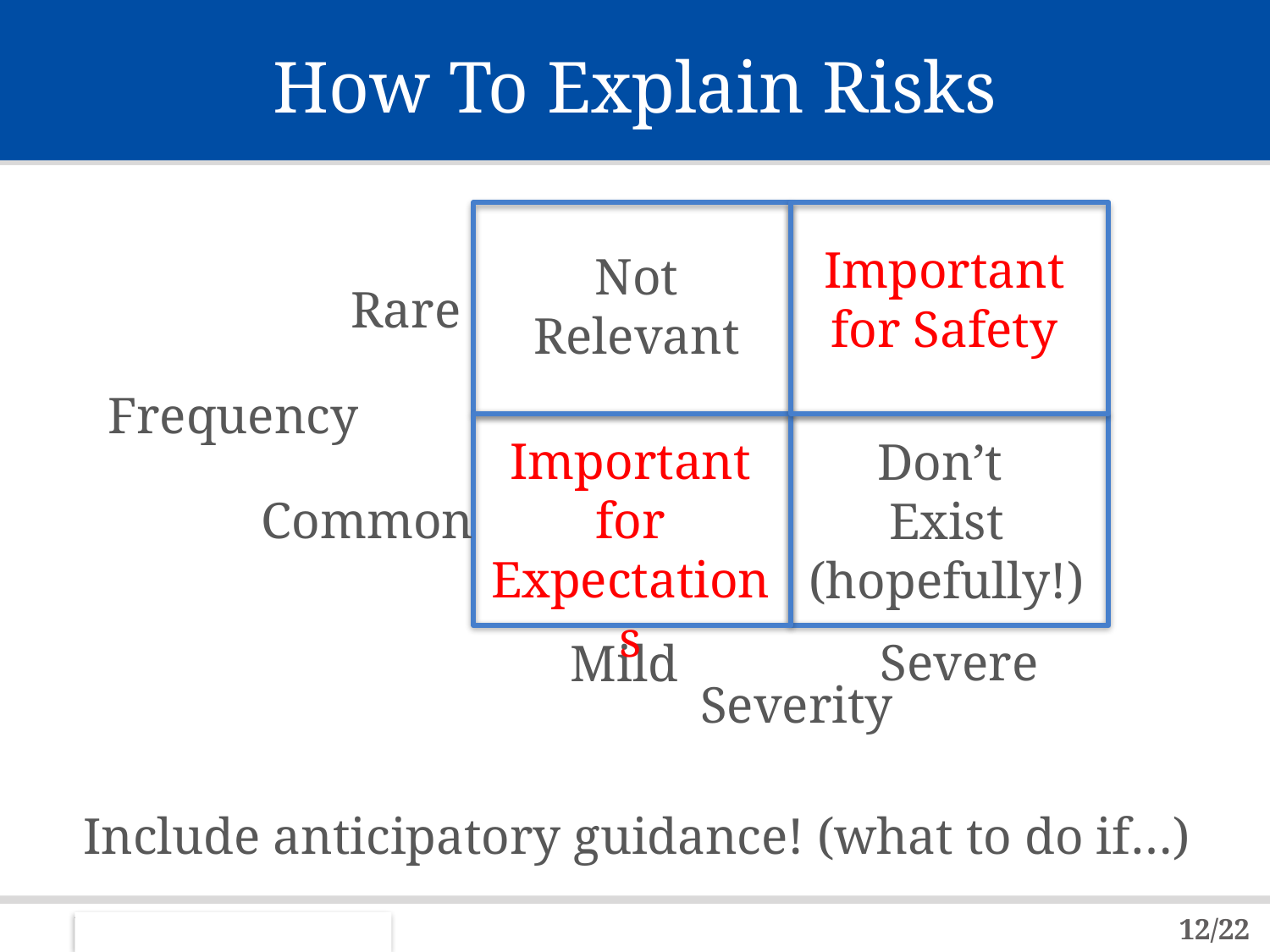

# How To Explain Risks
Important for Safety
Not
Relevant
Rare
Frequency
Important for Expectations
Don’t
Exist (hopefully!)
Common
Severe
Mild
Severity
Include anticipatory guidance! (what to do if…)
12/22

## Slide 14
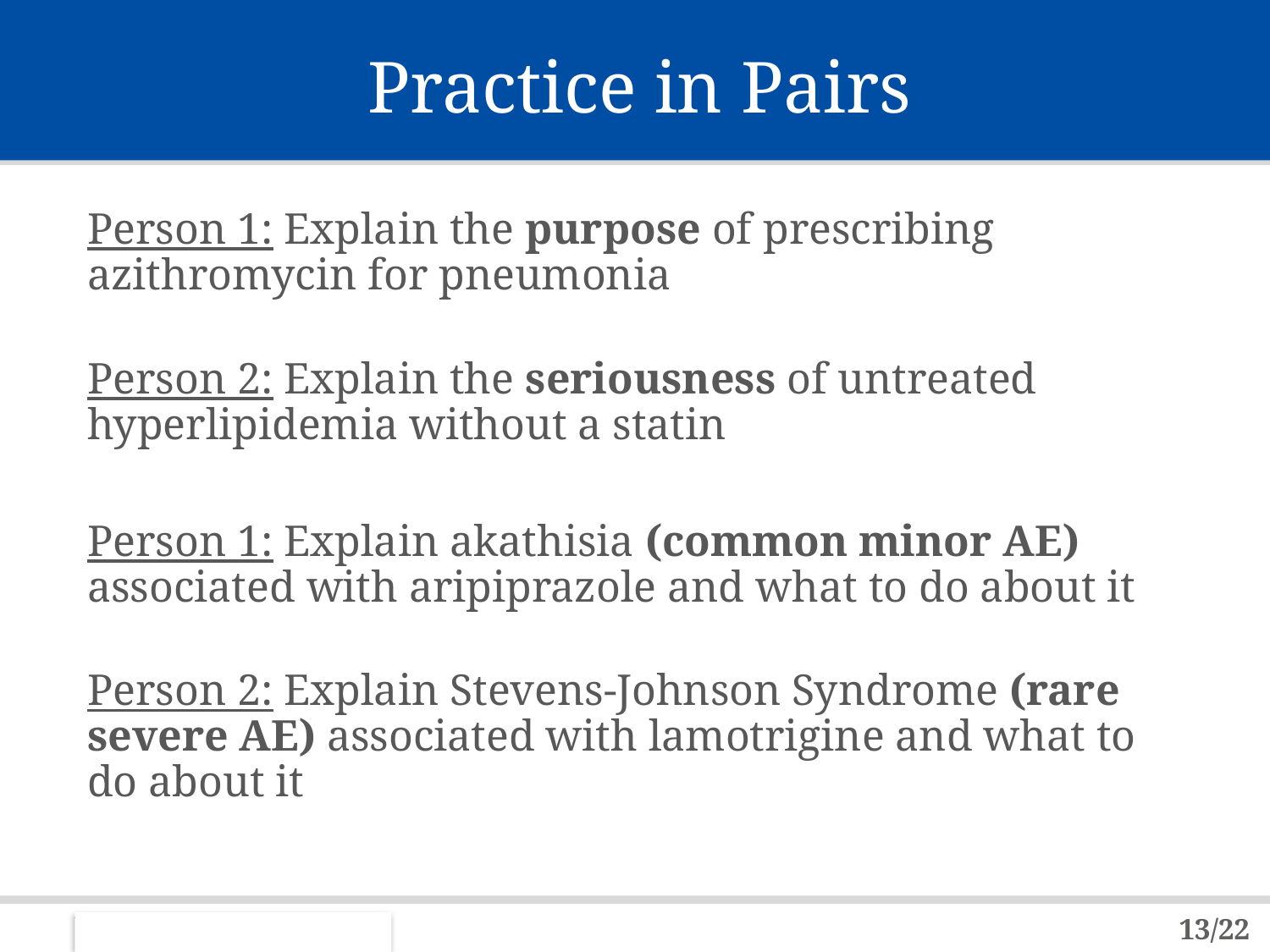

# Practice in Pairs
Person 1: Explain the purpose of prescribing azithromycin for pneumonia
Person 2: Explain the seriousness of untreated hyperlipidemia without a statin
Person 1: Explain akathisia (common minor AE) associated with aripiprazole and what to do about it
Person 2: Explain Stevens-Johnson Syndrome (rare severe AE) associated with lamotrigine and what to do about it
13/22

## Slide 15
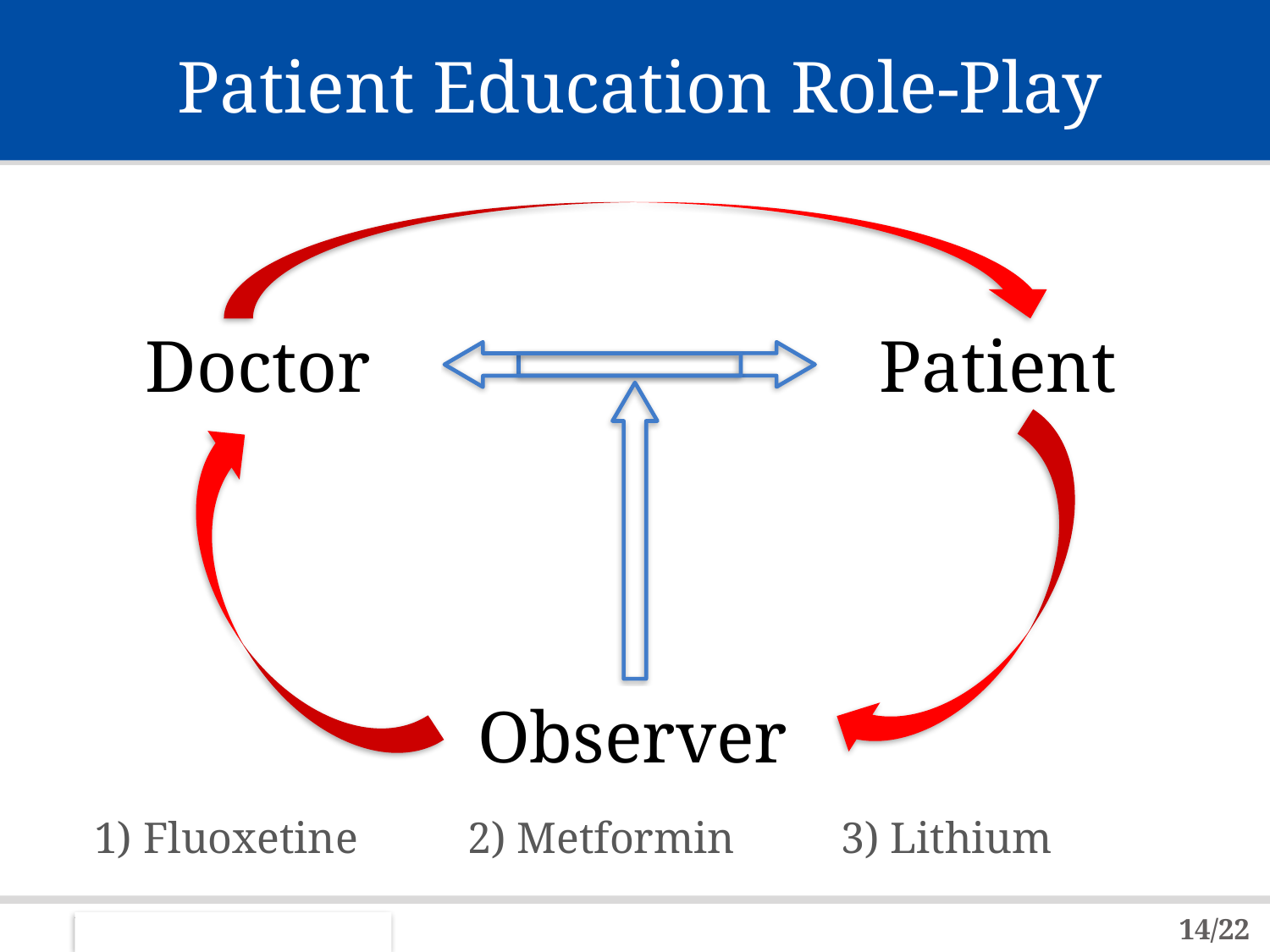

# Patient Education Role-Play
Doctor
Patient
Observer
2) Metformin
3) Lithium
1) Fluoxetine
14/22

## Slide 16
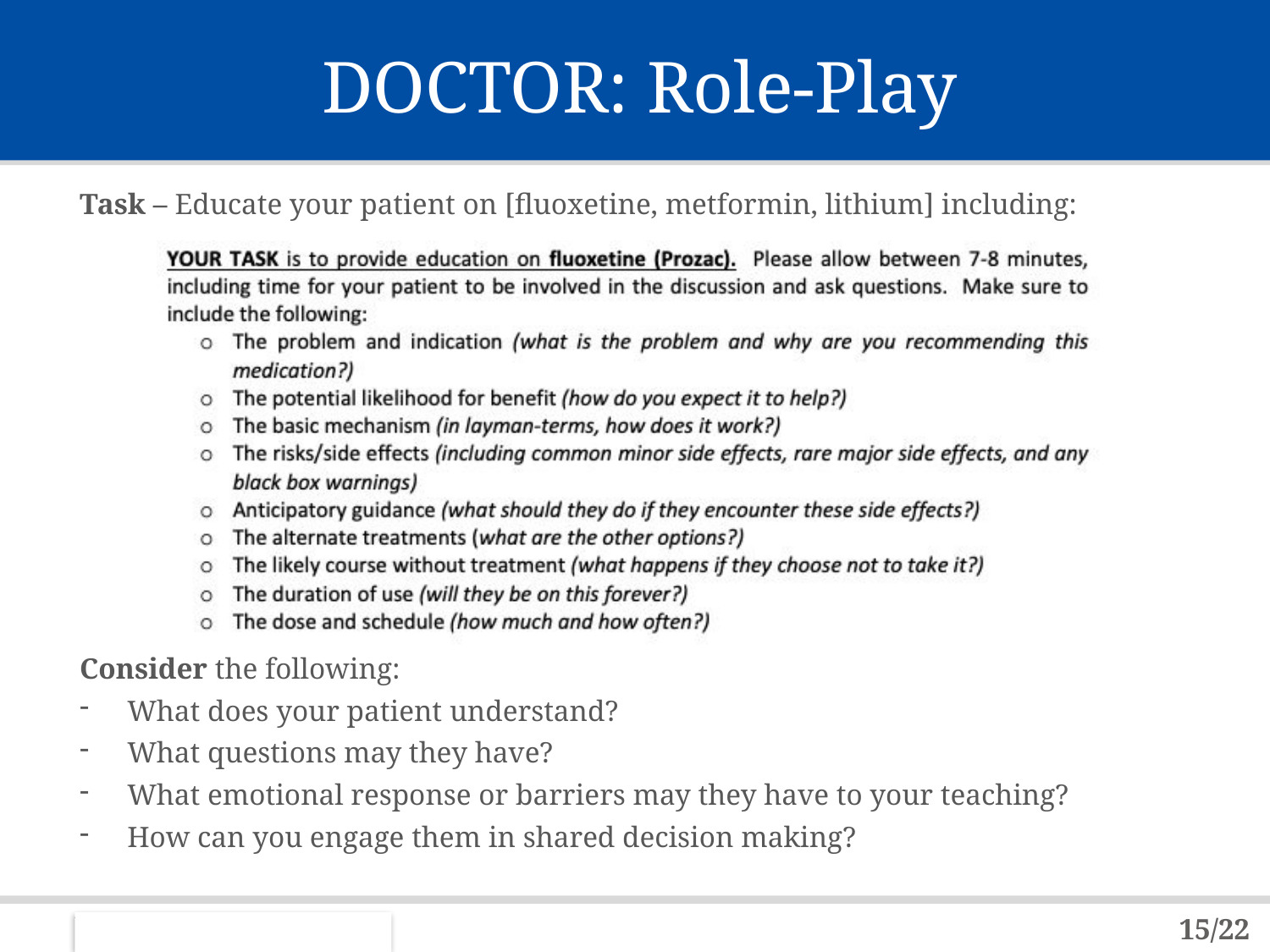

# DOCTOR: Role-Play
Task – Educate your patient on [fluoxetine, metformin, lithium] including:
Consider the following:
What does your patient understand?
What questions may they have?
What emotional response or barriers may they have to your teaching?
How can you engage them in shared decision making?
15/22

## Slide 17
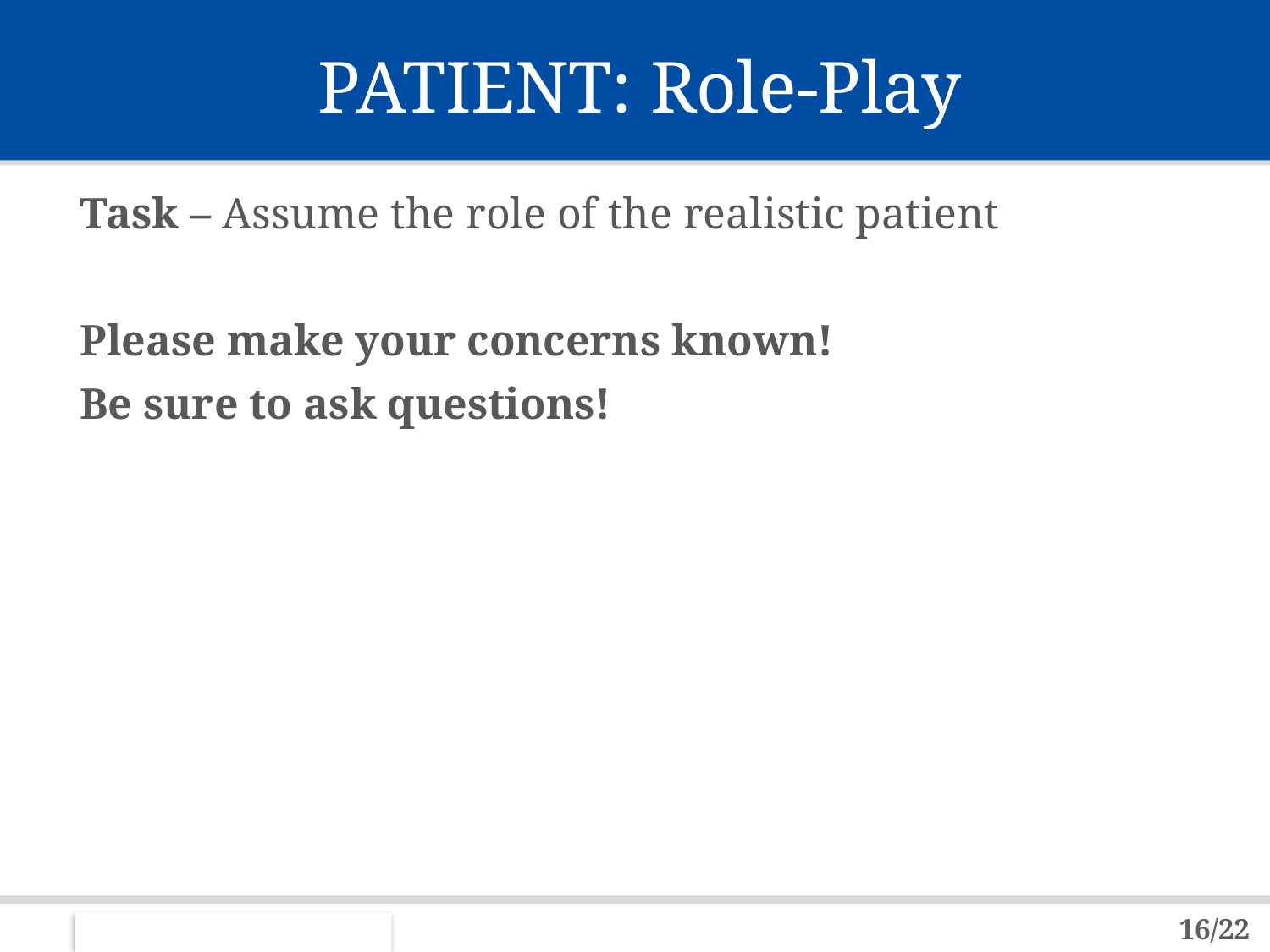

# PATIENT: Role-Play
Task – Assume the role of the realistic patient
Please make your concerns known!
Be sure to ask questions!
16/22

## Slide 18
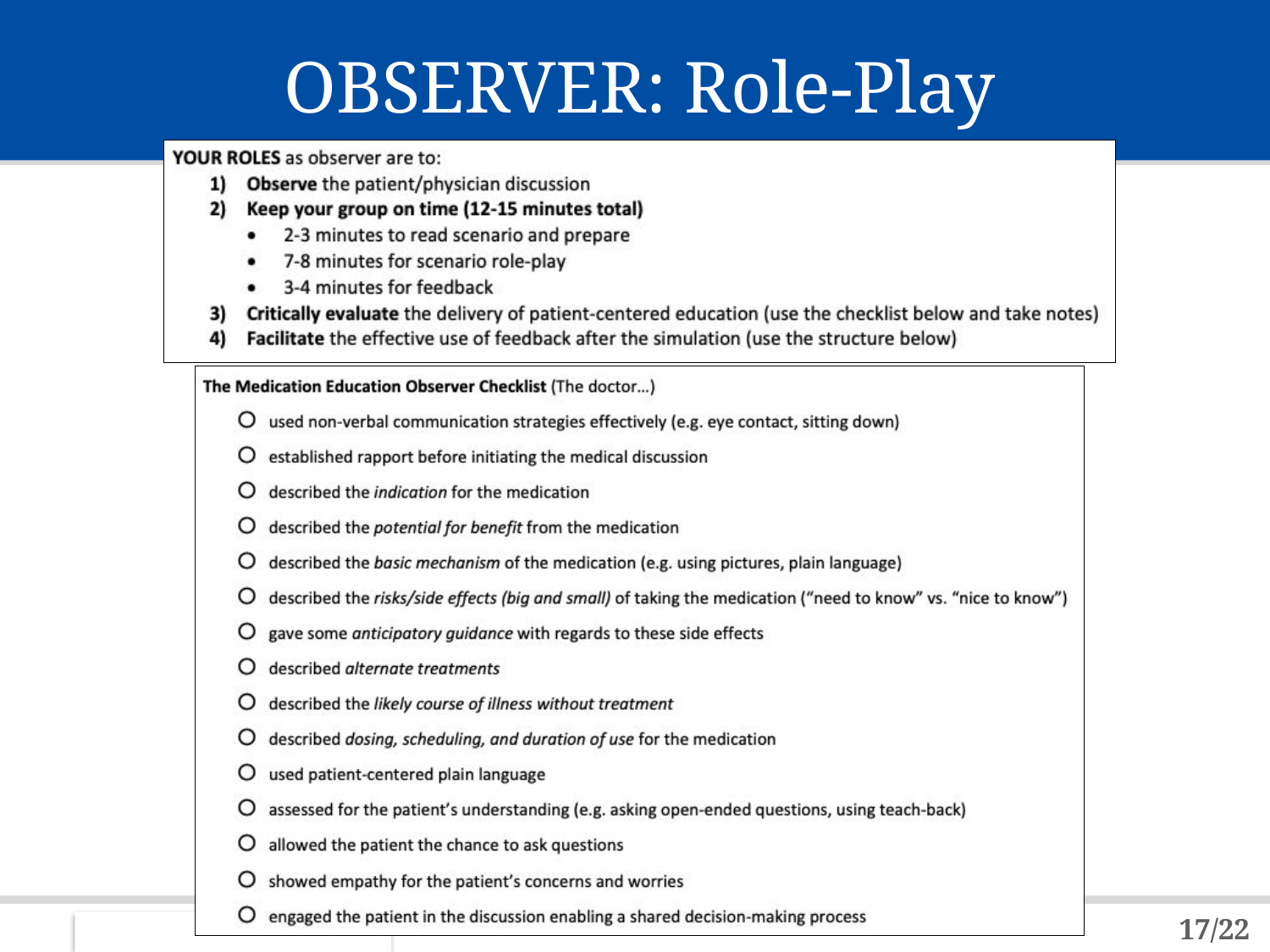

# OBSERVER: Role-Play
17/22

## Slide 19
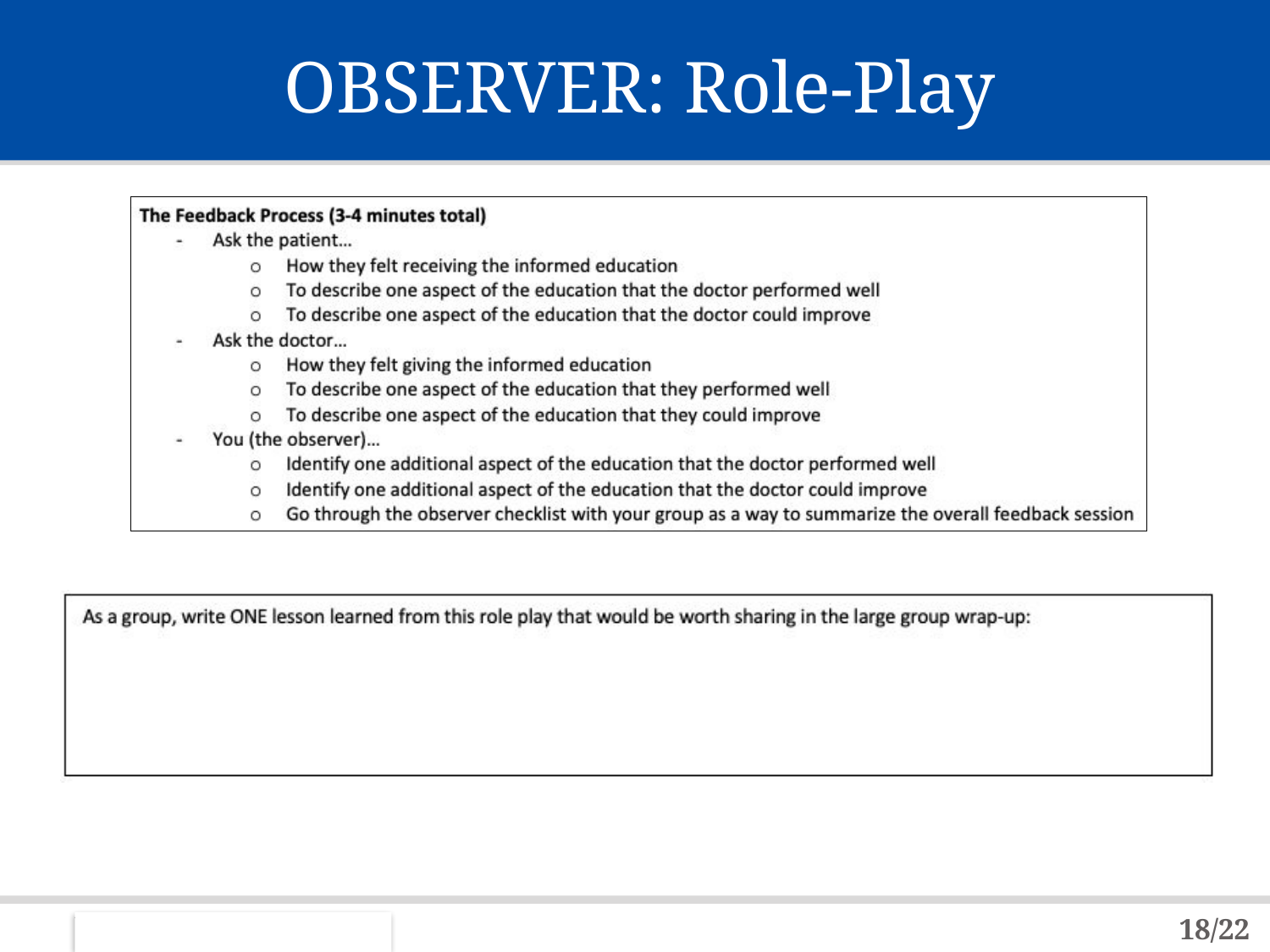

# OBSERVER: Role-Play
18/22

## Slide 20
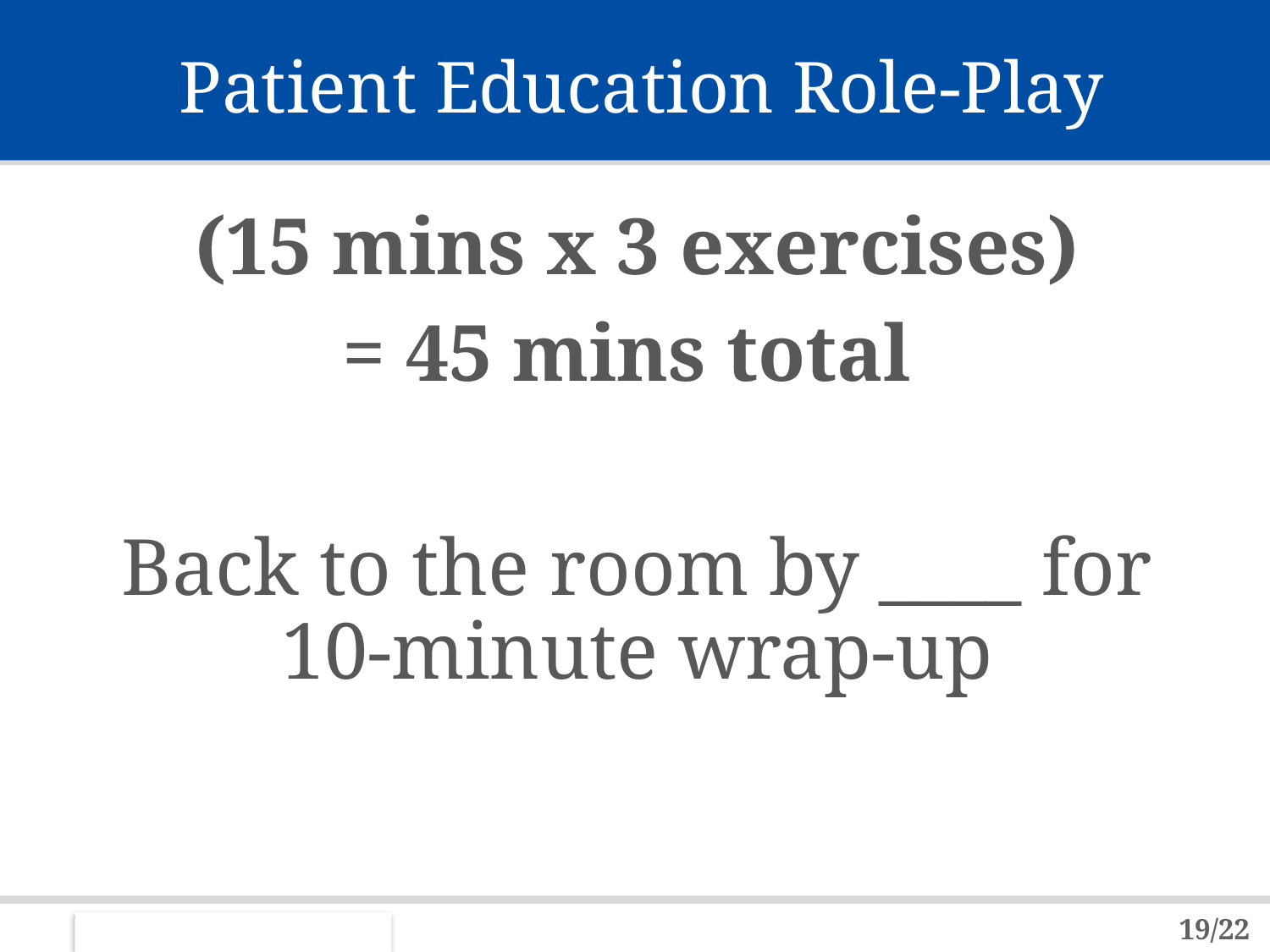

Patient Education Role-Play
(15 mins x 3 exercises)
= 45 mins total
Back to the room by ____ for 10-minute wrap-up
19/22

## Slide 21
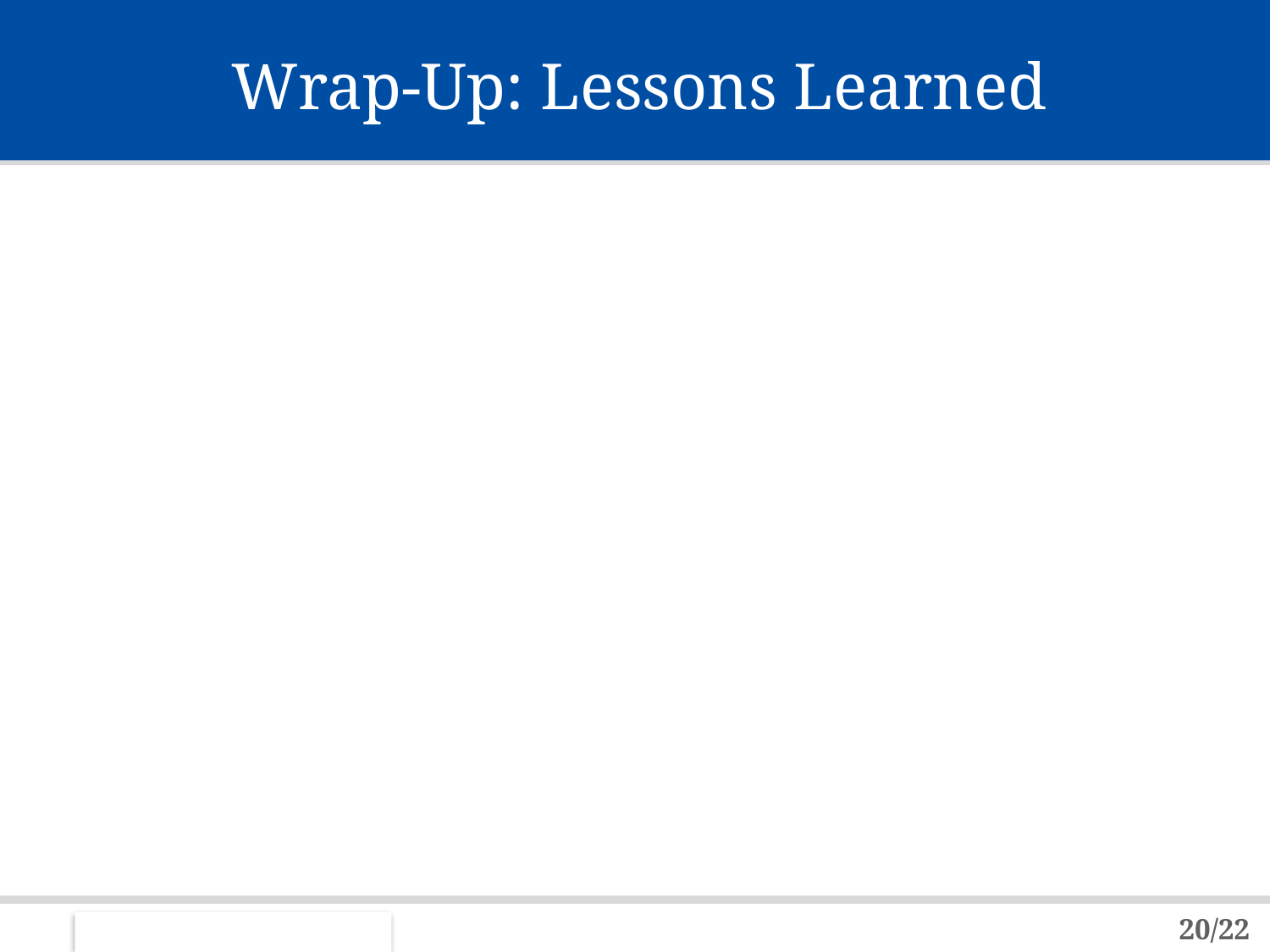

# Wrap-Up: Lessons Learned
20/22

## Slide 22
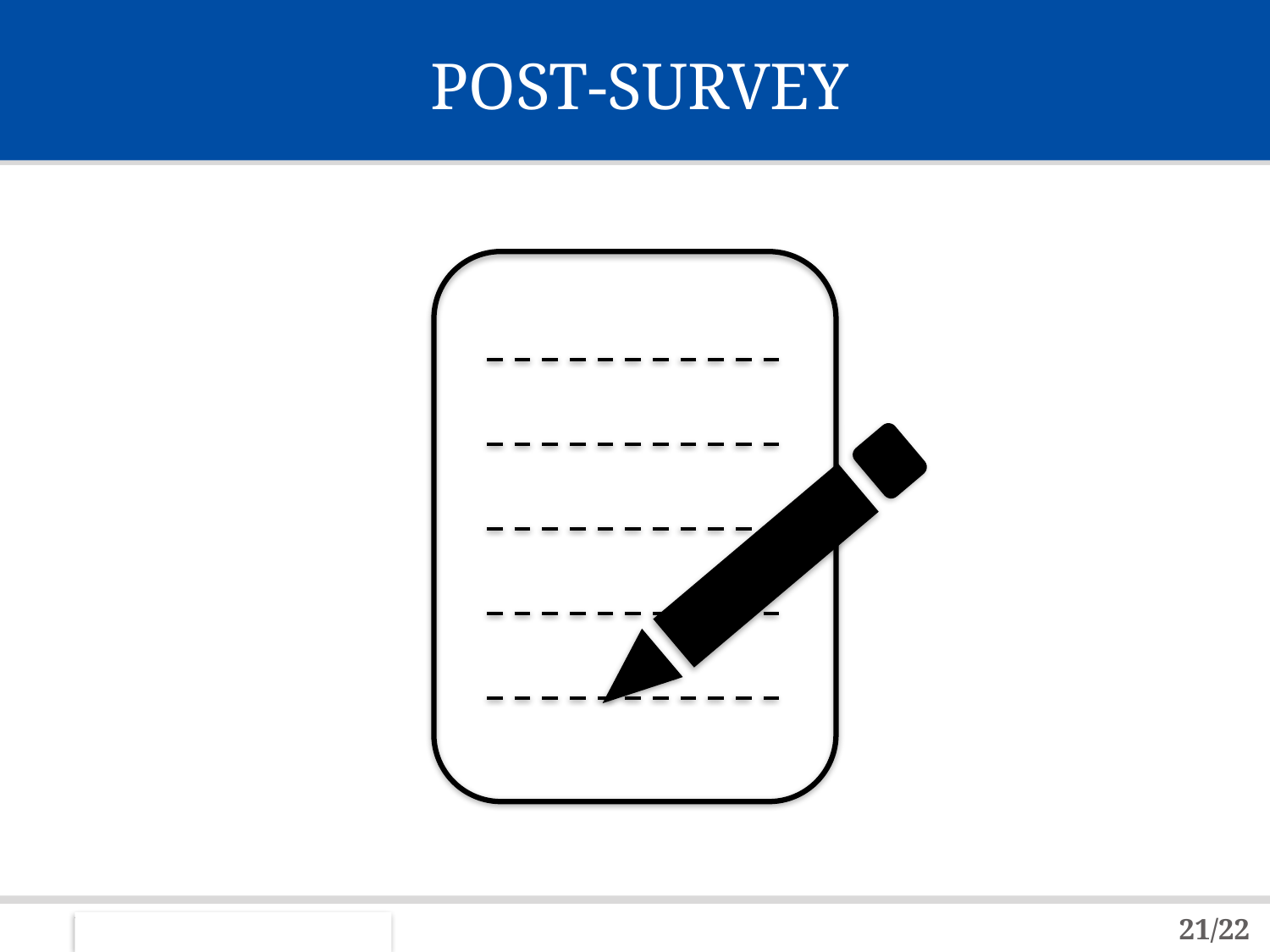

# POST-SURVEY
21/22

## Slide 23
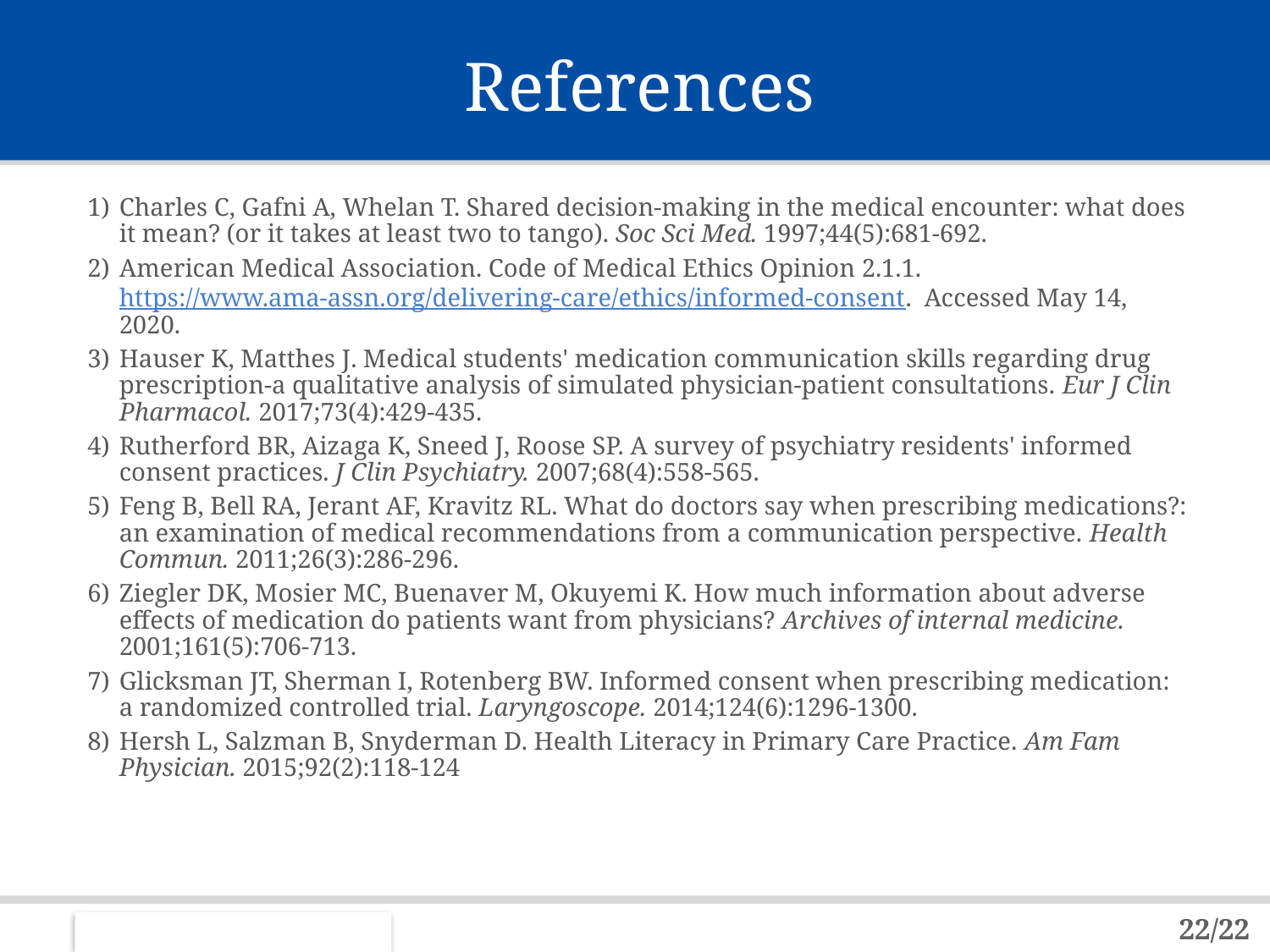

# References
Charles C, Gafni A, Whelan T. Shared decision-making in the medical encounter: what does it mean? (or it takes at least two to tango). Soc Sci Med. 1997;44(5):681-692.
American Medical Association. Code of Medical Ethics Opinion 2.1.1. https://www.ama-assn.org/delivering-care/ethics/informed-consent. Accessed May 14, 2020.
Hauser K, Matthes J. Medical students' medication communication skills regarding drug prescription-a qualitative analysis of simulated physician-patient consultations. Eur J Clin Pharmacol. 2017;73(4):429-435.
Rutherford BR, Aizaga K, Sneed J, Roose SP. A survey of psychiatry residents' informed consent practices. J Clin Psychiatry. 2007;68(4):558-565.
Feng B, Bell RA, Jerant AF, Kravitz RL. What do doctors say when prescribing medications?: an examination of medical recommendations from a communication perspective. Health Commun. 2011;26(3):286-296.
Ziegler DK, Mosier MC, Buenaver M, Okuyemi K. How much information about adverse effects of medication do patients want from physicians? Archives of internal medicine. 2001;161(5):706-713.
Glicksman JT, Sherman I, Rotenberg BW. Informed consent when prescribing medication: a randomized controlled trial. Laryngoscope. 2014;124(6):1296-1300.
Hersh L, Salzman B, Snyderman D. Health Literacy in Primary Care Practice. Am Fam Physician. 2015;92(2):118-124
22/22
