# Supplementary material for: When Doctor Means Teacher: An Interactive Workshop on Patient-Centered Education
Source: MedEdPORTAL. 2020 Dec 10;16:11053. doi: 10.15766/mep_2374-8265.11053 (PMC7732137; doi:10.15766/mep_2374-8265.11053)
Supplement: Supplementary file 1 — Facilitator Guide.docxPresurvey.docxSession 1 Patient Education Diagnoses.pptxVideo.mp4Session 1 Role-Play Scenarios.docxSession 1 Postsurvey.docxMedication Research Worksheet.docxSession 2 Patient Education Medications.pptxSession 2 Role-Play Scenarios.docxSession 2 Postsurvey.docx [file mep_2374-8265.11053-s001.zip › I. Session 2 Role-Play Scenarios.docx]

**SESSION 2 – PATIENT EDUCATION**

**MEDICATION EDUCATION ROLE PLAY MATERIALS**

**EXERCISE #1 – FLUOXETINE**

**Medication Education Role Play #1 – Fluoxetine – DOCTOR**

**YOU** are a primary care doctor at an outpatient clinic in a small town.

**YOUR PATIENT** is here for a routine check-up. However, in a waiting room screening form they scored 16/21 on the GAD-7 screening tool for generalized anxiety disorder. During the appointment they tell you they are generally very anxious and are interested in meeting with a therapist and also trying a medication. It is clear to you that fluoxetine would be a good choice.

- Demographics: 42yo, recently divorced, no kids, doctorate in chemical engineering
- Medical History: Irritable Bowel Syndrome, Migraines
- Family History: Bipolar Disorder (mother), Depression (brother), Panic Disorder (maternal aunt)
- Current Medications: Topiramate (migraine prophylaxis)
- Allergies: None

**YOUR TASK** is to provide education on **fluoxetine (Prozac).** Please allow between 7-8 minutes, including time for your patient to be involved in the discussion and ask questions. Make sure to include the following:

- The problem and indication *(what is the problem and why are you recommending this medication?)*
- The potential likelihood for benefit *(how do you expect it to help?)*
- The basic mechanism *(in layman-terms, how does it work?)*
- The risks/side effects *(including common minor side effects, rare major side effects, and any black box warnings)*
- Anticipatory guidance *(what should they do if they encounter these side effects?)*
- The alternate treatments (*what are the other options?)*
- The likely course without treatment *(what happens if they choose not to take it?)*
- The duration of use *(will they be on this forever?)*
- The dose and schedule *(how much and how often?)*

**CONSIDER** the following:

- How can you **engage your patient in shared decision-making**? It may be useful to ask for their input and thoughts on what you are sharing with them.
- **What does your patient understand** of the information you are sharing with them? It may be useful to use simple words/phrases, avoid giving too much information at once, and assess for their understanding.
- **What questions might you want answered** if you were in your patient’s shoes? It may be useful to anticipate these questions.
- **What barriers might your patient face** with remaining adherent to this medication? It may be useful to anticipate and explore these barriers.

**Medication Education Role Play #1 – Fluoxetine – PATIENT**

**YOU** are a patient going to meet with your primary care doctor for a routine check-up. In the waiting room you filled out a survey of anxiety symptoms and seemed to score quite highly, increasing your recent suspicions that your anxiety might be out of control over the past month. You know that you need help and are interested in meeting with a therapist and starting a medication.

- Demographics: 42yo, recently divorced, no kids, doctorate in chemical engineering
- Medical History: Irritable Bowel Syndrome, Migraines
- Family History: Bipolar Disorder (mother), Depression (brother), Panic disorder (maternal aunt)
- Current Medications: Topiramate (migraine prophylaxis)
- Allergies: None

**ADDITIONAL INFORMATION**

- Side effects are the biggest concern for you with any medication. When you were first diagnosed with migraines, the neurologist tried at least 3 other medications (you can’t remember all of the names) because you had a variety of side effects that made them intolerable to keep taking.
- Irritable bowel syndrome is one of the big stressors in your life and it seems to be exacerbated by your anxiety. You know that some medications can give people GI upset, and you are hoping that isn’t the case with the anti-anxiety medication.
- You recently got divorced but are enjoying a fulfilling new relationship in the past few weeks. Other friends have mentioned “psych meds” have some bad side effects on sexual performance, which seems like it might be a deal breaker in this new relationship.
- Being a chemical engineer, you’ve done some case studies on medications in the past and you are fascinated about the mechanism with which they work and achieve such drastic effects.

**YOUR TASK** is to assume the role of this patient as you meet your doctor who will provide information on fluoxetine and talk with you about his/her recommendations with regards to your health. Please make some of your concerns (above) known to your doctor. Also, be sure to ask questions if you think the patient you are role-playing may have some difficulty understanding.

**Medication Education Role Play #1 – Fluoxetine – OBSERVER**

**YOUR ROLES** as observer are to:

1. **Observe** the patient/physician discussion
2. **Keep your group on time (12-15 minutes total)**
   - 2-3 minutes to read scenario and prepare
   - 7-8 minutes for scenario role-play
   - 3-4 minutes for feedback
3. **Critically evaluate** the delivery of patient-centered education (use the checklist below and take notes)
4. **Facilitate** the effective use of feedback after the simulation (use the structure below)

**The Medication Education Observer Checklist** (The doctor…)

- used non-verbal communication strategies effectively (e.g. eye contact, sitting down)
- established rapport before initiating the medical discussion
- described the *indication* for the medication
- described the *potential for benefit* from the medication
- described the *basic mechanism* of the medication (e.g. using pictures, plain language)
- described the *risks/side effects (big and small)* of taking the medication (“need to know” vs. “nice to know”)
- gave some *anticipatory guidance* with regards to these side effects
- described *alternate treatments*
- described the *likely course of illness without treatment*
- described *dosing, scheduling, and duration of use* for the medication
- used patient-centered plain language
- assessed for the patient’s understanding (e.g. asking open-ended questions, using teach-back)
- allowed the patient the chance to ask questions
- showed empathy for the patient’s concerns and worries
- engaged the patient in the discussion enabling a shared decision-making process

**The Feedback Process (3-4 minutes total)**

- Ask the patient…
  - How they felt receiving the informed education
  - To describe one aspect of the education that the doctor performed well
  - To describe one aspect of the education that the doctor could improve
- Ask the doctor…
  - How they felt giving the informed education
  - To describe one aspect of the education that they performed well
  - To describe one aspect of the education that they could improve
- You (the observer)…
  - Identify one additional aspect of the education that the doctor performed well
  - Identify one additional aspect of the education that the doctor could improve
  - Go through the observer checklist with your group as a way to summarize the overall feedback session

As a group, write ONE lesson learned from this role play that would be worth sharing in the large group wrap-up:

**SESSION 2 – PATIENT EDUCATION**

**MEDICATION EDUCATION ROLE PLAY MATERIALS**

**EXERCISE #2 – METFORMIN**

**Medication Education Role Play #2 – Metformin – DOCTOR**

**YOU** are a primary care doctor at the Veteran’s Hospital.

**YOUR PATIENT** recently had an elevated hemoglobin A1c (7.3%) during annual screening labs, consistent with diabetes. They were unable to show any improvement with dietary modifications and exercise – repeat A1c was 7.4%. At this point, you think it wise to initiate the patient on metformin as first-line treatment.

- Demographics: 46-year-old, married with no kids, prior service in the Air Force, now a manager at a local restaurant
- Medical History: HTN, Stage 2 CKD (GFR 70ml/min), anxiety, overweight (BMI 29), Crohn’s disease
- Family History: Type 2 diabetes (mother, older sister, maternal aunt)
- Current Medications: amlodipine, lisinopril, sertraline, methotrexate
- Allergies: penicillin (hives)

**YOUR TASK** is to provide education on **metformin.** Please allow between 7-8 minutes, including time for your patient to be involved in the discussion and ask questions. Make sure to include the following:

- The problem and indication *(what is the problem and why are you recommending this medication?)*
- The potential likelihood for benefit *(how do you expect it to help?)*
- The basic mechanism *(in layman-terms, how does it work?)*
- The risks/side effects *(including common minor side effects, rare major side effects, and any black box warnings)*
- Anticipatory guidance *(what should they do if they encounter these side effects?)*
- The alternate treatments (*what are the other options?)*
- The likely course without treatment *(what happens if they choose not to take it?)*
- The duration of use *(will they be on this forever?)*
- The dose and schedule *(how much and how often?)*

**CONSIDER** the following:

- How can you **engage your patient in shared decision-making**? It may be useful to ask for their input and thoughts on what you are sharing with them.
- **What does your patient understand** of the information you are sharing with them? It may be useful to use simple words/phrases, avoid giving too much information at once, and assess for their understanding.
- **What questions might you want answered** if you were in your patient’s shoes? It may be useful to anticipate these questions.
- **What barriers might your patient face** with remaining adherent to this medication? It may be useful to anticipate and explore these barriers.

**Medication Education Role Play #2 – Metformin – PATIENT**

**YOU** are a patient at the local Veteran’s Hospital who is returning to your PCP for a follow-up appointment. On your annual screening labs, a blood test came back positive for diabetes. Your doctor wanted you to make changes to your diet and exercise habits before starting medication, but despite cutting back on a few of your guilty pleasures, it doesn’t seem to have made a meaningful difference.

- Demographics: 46-year-old, married with no kids, prior service in the Air Force, now a manager at a local restaurant
- Medical History: HTN, Stage 2 CKD (GFR 70ml/min), anxiety, overweight (BMI 29), Crohn’s disease
- Family History: Type 2 diabetes (mother, older sister, maternal aunt)
- Current Medications: amlodipine, lisinopril, sertraline, methotrexate
- Allergies: penicillin (hives)

**ADDITIONAL INFORMATION**

- Many of your family members have diabetes (see above) and they all take multiple medications for it. It seems that, despite these medications, they still have difficulty controlling their blood sugar and you are not convinced that diabetes medicines actually work.
- Of all your health conditions, Crohn’s disease is the most debilitating. You’ve recently gotten your diarrhea under control on methotrexate and don’t want to start a new medication that might upset your gastrointestinal system all over again.
- Last time you started a new medication (sertraline for anxiety), your doctor didn’t explain any of the risks or side effects. For the first couple weeks you actually felt more anxious and even suicidal at first. After learning that this is a known potential side effect, you fired your psychiatrist and are considering legal action for negligence.

**YOUR TASK** is to assume the role of this patient as you meet your doctor who will provide information on metformin and talk with you about his/her recommendations with regards to your health. Please make some of your concerns (above) known to your doctor. Also, be sure to ask questions if you think the patient you are role-playing may have some difficulty understanding.

**Medication Education Role Play #2 – Metformin – OBSERVER**

**YOUR ROLES** as observer are to:

1. **Observe** the patient/physician discussion
2. **Keep your group on time (12-15 minutes total)**
   - 2-3 minutes to read scenario and prepare
   - 7-8 minutes for scenario role-play
   - 3-4 minutes for feedback
3. **Critically evaluate** the delivery of patient-centered education (use the checklist below and take notes)
4. **Facilitate** the effective use of feedback after the simulation (use the structure below)

**The Medication Education Observer Checklist** (The doctor…)

- used non-verbal communication strategies effectively (e.g. eye contact, sitting down)
- established rapport before initiating the medical discussion
- described the *indication* for the medication
- described the *potential for benefit* from the medication
- described the *basic mechanism* of the medication (e.g. using pictures, plain language)
- described the *risks/side effects (big and small)* of taking the medication (“need to know” vs. “nice to know”)
- gave some *anticipatory guidance* with regards to these side effects
- described *alternate treatments*
- described the *likely course of illness without treatment*
- described *dosing, scheduling, and duration of use* for the medication
- used patient-centered plain language
- assessed for the patient’s understanding (e.g. asking open-ended questions, using teach-back)
- allowed the patient the chance to ask questions
- showed empathy for the patient’s concerns and worries
- engaged the patient in the discussion enabling a shared decision-making process

**The Feedback Process (3-4 minutes total)**

- Ask the patient…
  - How they felt receiving the education
  - To describe one aspect of the education that the doctor performed well
  - To describe one aspect of the education that the doctor could improve
- Ask the doctor…
  - How they felt giving the education
  - To describe one aspect of the education that they performed well
  - To describe one aspect of the education that they could improve
- You (the observer)…
  - Identify one additional aspect of the education that the doctor performed well
  - Identify one additional aspect of the education that the doctor could improve
  - Go through the observer checklist with your group as a way to summarize the overall feedback session

As a group, write ONE lesson learned from this role play that would be worth sharing in the large group wrap-up:

**SESSION 2 – PATIENT EDUCATION**

**MEDICATION EDUCATION ROLE PLAY MATERIALS**

**EXERCISE #3 – LITHIUM**

**Medication Education Role Play #3 – Lithium – DOCTOR**

**YOU** are an outpatient psychiatrist who runs a private practice clinic in a suburban area.

**YOUR PATIENT** is a 28-year-old with a history of depression. The last depressive episode was over 3 years ago, and you had been considering tapering their antidepressant. However, for the past month they are not sleeping much, talking slightly quicker, and apparently experiencing some marital difficulties due to out-of-character flirtatious behavior. You think this could be an emerging manic episode and that they would benefit from a mood stabilizer.

- Demographics: 28 years old, married (but going through conflict and counseling), two young children (3 and 5), working as a Nurse Practitioner at a pulmonology office
- Medical History: Hypothyroidism, depression, low back pain
- Family History: Bipolar disorder (sister), Diabetes/CKD (father)
- Current Medications: Levothyroxine, escitalopram, ibuprofen PRN
- Allergies: Tylenol

**YOUR TASK** is to provide education on **lithium.** Please allow between 7-8 minutes, including time for your patient to be involved in the discussion and ask questions. Make sure to include the following:

- The problem and indication *(what is the problem and why are you recommending this medication?)*
- The potential likelihood for benefit *(how do you expect it to help?)*
- The basic mechanism *(in layman-terms, how does it work?)*
- The risks/side effects *(including common minor side effects, rare major side effects, and any black box warnings)*
- Anticipatory guidance *(what should they do if they encounter these side effects?)*
- The alternate treatments (*what are the other options?)*
- The likely course without treatment *(what happens if they choose not to take it?)*
- The duration of use *(will they be on this forever?)*
- The dose and schedule *(how much and how often?)*

**CONSIDER** the following:

- How can you **engage your patient in shared decision-making**? It may be useful to ask for their input and thoughts on what you are sharing with them.
- **What does your patient understand** of the information you are sharing with them? It may be useful to use simple words/phrases, avoid giving too much information at once, and assess for their understanding.
- **What questions might you want answered** if you were in your patient’s shoes? It may be useful to anticipate these questions.
- **What barriers might your patient face** with remaining adherent to this medication? It may be useful to anticipate and explore these barriers.

**Medication Education Role Play #3 – Lithium – PATIENT**

**YOU** are meeting with your private psychiatrist in an office setting. They treat you for depression, however in the last month you’ve felt the opposite of depressed – you have not needed much sleep, have been talking and thinking more quickly, and have been doing some out-of-character things such as flirting with strangers. Due to this behavior, your marriage has been experiencing difficulties and your spouse dragged you back to the psychiatrist early. Overall, you like the way you feel, but you know that something is “off.” Your psychiatrist seems to have picked up on it.

- Demographics: 28 years old, married (but going through conflict and counseling), two young children (3 and 5), working as a Nurse Practitioner at a pulmonology office
- Medical History: Hypothyroidism, depression, low back pain
- Family History: Bipolar disorder (sister), Diabetes/CKD (father)
- Current Medications: Levothyroxine, escitalopram, ibuprofen PRN
- Allergies: Tylenol

**ADDITIONAL INFORMATION**

- Although you know something is “off”, being in this “manic” state feels pretty good – you can be productive without sleep and it is thrilling to flirt with people other than your spouse. Your psychiatrist thinks that medications are always the answer, but you are ambivalent about whether you need any medications right now since this is much better than feeling depressed.
- For the past year your primary care doctor has been constantly changing your levothyroxine dose as your thyroid levels seem to be all over the place. Finally, it seems to have stabilized on your current dose. You know that some medications interfere with the thyroid and you are wary of upsetting the recent homeostasis.
- Your sister has bipolar disorder and takes lithium. Although it helps her be more stable, she has gained over 50 lbs and has acne all over her face since starting lithium – this doesn’t seem worth it to you.

**YOUR TASK** is to assume the role of this patient as you meet your doctor who will provide information on lithium and talk with you about his/her recommendations with regards to your health. Please make some of your concerns (above) known to your doctor. Also, be sure to ask questions if you think the patient you are role-playing may have some difficulty understanding.

**Medication Education Role Play #3 – Lithium – OBSERVER**

**YOUR ROLES** as observer are to:

1. **Observe** the patient/physician discussion
2. **Keep your group on time (12-15 minutes total)**
   - 2-3 minutes to read scenario and prepare
   - 7-8 minutes for scenario role-play
   - 3-4 minutes for feedback
3. **Critically evaluate** the delivery of patient-centered education (use the checklist below and take notes)
4. **Facilitate** the effective use of feedback after the simulation (use the structure below)

**The Medication Education Observer Checklist** (The doctor…)

- used non-verbal communication strategies effectively (e.g. eye contact, sitting down)
- established rapport before initiating the medical discussion
- described the *indication* for the medication
- described the *potential for benefit* from the medication
- described the *basic mechanism* of the medication (e.g. using pictures, plain language)
- described the *risks/side effects (big and small)* of taking the medication (“need to know” vs. “nice to know”)
- gave some *anticipatory guidance* with regards to these side effects
- described *alternate treatments*
- described the *likely course of illness without treatment*
- described *dosing, scheduling, and duration of use* for the medication
- used patient-centered plain language
- assessed for the patient’s understanding (e.g. asking open-ended questions, using teach-back)
- allowed the patient the chance to ask questions
- showed empathy for the patient’s concerns and worries
- engaged the patient in the discussion enabling a shared decision-making process

**The Feedback Process (3-4 minutes total)**

- Ask the patient…
  - How they felt receiving the education
  - To describe one aspect of the education that the doctor performed well
  - To describe one aspect of the education that the doctor could improve
- Ask the doctor…
  - How they felt giving the education
  - To describe one aspect of the education that they performed well
  - To describe one aspect of the education that they could improve
- You (the observer)…
  - Identify one additional aspect of the education that the doctor performed well
  - Identify one additional aspect of the education that the doctor could improve
  - Go through the observer checklist with your group as a way to summarize the overall feedback session

As a group, write ONE lesson learned from this role play that would be worth sharing in the large group wrap-up:
