# Supplementary material for: When Doctor Means Teacher: An Interactive Workshop on Patient-Centered Education
Source: MedEdPORTAL. 2020 Dec 10;16:11053. doi: 10.15766/mep_2374-8265.11053 (PMC7732137; doi:10.15766/mep_2374-8265.11053)
Supplement: Supplementary file 1 — Facilitator Guide.docxPresurvey.docxSession 1 Patient Education Diagnoses.pptxVideo.mp4Session 1 Role-Play Scenarios.docxSession 1 Postsurvey.docxMedication Research Worksheet.docxSession 2 Patient Education Medications.pptxSession 2 Role-Play Scenarios.docxSession 2 Postsurvey.docx [file mep_2374-8265.11053-s001.zip › _Educational_Summary_Report_11053.pdf]

MedEdPORTAL® | The AAMC Journal of Teaching and Learning Resources

Original Publication Open Access

When Doctor Means Teacher: An Interactive Workshop on Patient-Centered Education

Thomas O. Mitchell, MD*, Matthew N. Goldenberg, MD, MSc

*Corresponding author: tomitch10@gmail.com

Abstract

Introduction: Increasingly, health care is delivered through a patient-centered model, and patients engage in shared decision-making with their medical providers. As a result, medical educators are placing more emphasis on patient-centered communication skills. However, few published curricula currently offer a comprehensive discussion of skills for providing patient-centered education (PCE), a key component of shared decision-making. We developed an interactive, two-session workshop aiming to improve students' abilities to provide PCE. **Methods:** Our workshop included didactic instruction, group discussion, and interactive simulations. The workshop was delivered to 50 clinical clerkship medical students. The first session concentrated on educating patients about their diagnoses, while the second session focused on providing patients with information about medications and other treatments. We used detailed and realistic role-play exercises as a core tool for student practice and demonstration of confidence. To evaluate the workshop, we used pre- and postsurveys. **Results:** The sessions were well received by students, who strongly agreed both before and after the workshop that PCE was an important skill. Students also strongly agreed that the role-play exercises were an effective tool for learning PCE. They demonstrated significant improvements in their confidence to name important elements of PCE and to deliver PCE in the future. **Discussion:** This workshop fills a curricular gap in offering a comprehensive and interactive curriculum for improving students' abilities to provide critical PCE. The format and content should be easily adaptable to various disciplines, learners, and teaching modalities.

Keywords
Patient Education, Health Literacy, Shared Decision-Making, Communication Skills, Patient-Centered, Role-Play, Primary Care, Psychiatry

Educational Objectives

By the end of this activity, learners will be able to:

1. Explain the importance of providing patient-centered education for diagnoses and medications.
2. Describe five key elements of patient-centered education for diagnoses and medications.
3. Demonstrate confidence in patient-centered education for diagnoses and medications.

Introduction

In recent decades, health care has increasingly been delivered through a patient-centered model.[1,2] Within this model, value is placed on an individual's specific needs and desired health outcomes, which then become the driving force behind health discussions and decisions.[3] A major role of patient-centered health care providers has been to educate, equip, and empower patients through shared decision-making (SDM).

We define patient-centered education (PCE) as providing patients with the information they need to meaningfully participate in the decision-making process,[4] while paying particular attention to their level of health literacy.[5] Within an SDM framework, high-quality PCE has been shown to improve a variety of outcomes including patient satisfaction, treatment adherence, and even health-related measures.[6-8] Additionally, evidence from qualitative studies eliciting perspectives of patients in a variety of settings suggests that a majority desire to be informed about their diagnoses and treatments while being included in decisions.[9-12] However, similar studies have also found a disconnect: Patients often do not feel heard, valued, or included[9,12]; doctors often fail to communicate adequate information[13]; and the information that is communicated is often difficult for patients to comprehend.[14,15]

Medical schools have steadily introduced curricula that emphasizes patient-centered communication skills, with many

Citation:
Mitchell TO, Goldenberg MN. When doctor means teacher: an interactive workshop on patient-centered education. *MedEdPORTAL*. 2020;16:11053. https://doi.org/10.15766/mep_2374-8265.11053

Copyright © 2020 Mitchell and Goldenberg This is an open-access publication distributed under the terms of the Creative Commons Attribution license.

focused on building rapport, using open-ended questioning, conveying empathy, and delivering bad news.[16-19] A number of experiential interventions involve students providing direct patient education[20] through isolated exercises such as translating medical documents into plain language,[14] writing letters to patients explaining common conditions,[21] following up with patients via telephone after an office visit,[22] and explaining medications for heart failure.[23] However, these cited interventions are one-off exercises in specific clinical scenarios, each emphasizing a different technique of patient education. Our workshop aims to be a comprehensive training focused on rationale, techniques, and practice for effectively educating patients and their families.

Role-play exercises are frequently used for skill development in clinical education, particularly when access to real or standardized patients is challenging. Despite mixed student sentiments associated with role-play exercises, they have been found to be a useful educational tool, especially when the role-plays are sufficiently planned, are well structured, and include situations that closely mirror the learner's reality.[24]

High-quality PCE is desired by patients, effective in improving patient outcomes, and currently not well executed by providers. Given the trend towards curricula emphasizing patient-centered skills, it is necessary that medical schools respond by incorporating robust education in how to provide PCE. We seek to fill this gap with this interactive, two-session workshop.

Methods

We designed an interactive, two-session workshop aimed at improving clerkship medical students' abilities to provide PCE. The sessions included didactic instruction, group discussion, and role-play exercises, as described in the facilitator guide (Appendix A). Both sessions followed a similar flow that closely mirrored the learning objectives: (1) a 15-minute instructor-led introduction and didactic portion that presented background information with both subjective and objective arguments to convince students of the importance of providing PCE, (2) a 20-minute interactive portion focused on discussing and practicing the necessary skills for providing PCE, and (3) a 45-minute portion dedicated to role-play exercises to demonstrate confidence in PCE. With a 10-minute wrap-up period, each session lasted approximately 1.5 hours.

We piloted the first session in late 2019 with 20 students in the primary care and psychiatry clerkship, close to the end of their clerkship year. Utilizing student feedback, we altered both sessions slightly. We then taught the full workshop in 2 consecutive weeks in early 2020 to a new group of students just beginning their clerkship year. Twenty-seven students participated in the first session, with three additional students (30 total) participating in the second session.

Students were encouraged to read "Health Literacy in Primary Care Practice"[5] as prereading prior to the first session. The first session began with a presurvey (Appendix B) and was guided by PowerPoint slides (Appendix C). After presenting background information (slides 4-10), we used a video created at our institution and author owned (Appendix D; slide 11) to discuss key elements of PCE. This video showed a student providing education to a patient about his diagnosis and included a combination of positive and negative elements that participants could identify. Using lessons learned from the video, we referenced the assigned reading to formally delineate important skills needed when providing PCE (slide 14). These skills included avoiding medical jargon, breaking down information or instructions into small concrete steps, limiting the focus of a visit to three key points or tasks, and assessing for comprehension. We further discussed key elements of PCE by referring to the Agency for Healthcare Quality and Research's Health Literacy Universal Precautions Toolkit (slide 15).[25] To provide more specific instruction on assessing for comprehension, we explained the teach-back method[26] in detail and offered examples (slide 16) of how to initiate this skill using nonshaming, open-ended questioning. We then asked students to pair up and practice these skills by breaking down confusing medical terminology into patient-centered language (slide 17). This exercise was followed by a debrief to address the challenges and key learning points. The remainder of the first session was dedicated to role-play exercises focused on providing PCE over three common diagnoses: hypertension, depression, and diabetes. (With some revision, these cases could be altered to reflect other relevant conditions as appropriate for the learners.) Students were separated into groups of three with each student having the opportunity to play the role of doctor, patient, and observer. Specific and detailed case background, instructions, and helpful hints were given for each case and each role (Appendix E). In order to mirror a realistic patient interaction, the doctor and patient had some consistent information but also some different, ambiguous, and/or contradictory information. Also, in order to facilitate a more active role for the observers, they were given a number of specific tasks including keeping time, monitoring a detailed checklist, and facilitating effective feedback. The students were sent to various small breakout rooms to enable privacy while completing the exercises. During this time, the instructors floated between groups to observe

Copyright © 2020 Mitchell and Goldenberg This is an open-access publication distributed under the terms of the Creative Commons Attribution license.

and offer real-time feedback. Upon completion, the instructor led a short wrap-up session and administered the postsurvey (Appendix F). Finally, a homework assignment (Appendix G) for the second session was handed out. This assignment guided students to systematically research characteristics of three medications (fluoxetine, metformin, lithium) as preparation for a similar role-play exercise during the next session.

The second session was also guided by PowerPoint slides (Appendix H) and followed a similar structure, but with a focus on educating patients about medications and other treatments. The session began with a brief recap of the first session. Given the focus on treatment, the instructor presented an overview of the relevant concepts of SDM and informed consent (slides 4-6). The instructor then shared background specifically on PCE for medications and why it was an important skill (slides 7-9). The article by Feng, Bell, Jerant, and Kravitz[13] was cited to highlight best practices and important elements when educating patients about medications (slide 11). We then shared our own conceptual framework for discussing medication side effects (slide 12), given that this can be a challenge for even the most experienced providers. Next, we asked students to pair up and practice specific aspects of medication-focused education such as describing a side effect, rationale for taking a medication, and so on (slide 13). This exercise was followed by a debrief to address the challenges and key learning points. The remainder of the second session was dedicated to role-play exercises (Appendix I) with a similar structure to those in the first session. Students played the roles of doctor, patient, and observer in specific and realistic scenarios in which fluoxetine, metformin, and lithium were being recommended. Students were able to use the assigned research they had done on these medications to aid them. (The role-play exercises and homework could conceivably be revised to cover education about other treatment interventions—e.g., surgical procedures, blood transfusion, etc.—depending on the audience.) The session concluded with a 10-minute instructor-led wrap-up session and a postsurvey (Appendix J).

We evaluated the workshop with surveys featuring statements related to the learning objectives, using a Likert scale for level of agreement. We also asked students about their experience of the role-play scenarios given that these were a core component of our workshop. Students completed a presurvey (Appendix B) prior to the first session and postsurveys (Appendices F and J) following each session. Students also had the option to provide additional feedback in a free-text response. Survey results from the pilot session were used to inform changes prior to full implementation. We compiled and analyzed responses from the first full two-session workshop. Results of the matched responses from pre- and postsurveys were compared using *t* tests, and statistical significance was defined as $p < .05$.

Results

Our workshop was implemented with 50 students total. In late 2019, 20 medical students participated in the pilot session during the primary care and psychiatry clerkship. In early 2020, 30 clerkship medical students participated in one or both sessions of the workshop, which were held over consecutive weeks. We facilitated the sessions, each of which lasted 1.5 hours.

At the conclusion of both sessions, students felt that the educational objectives had been met (see Table 1). Both before and after the intervention, students strongly agreed that delivering PCE was an important skill (4.9 preworkshop vs. 4.9 postsession 1 and 4.9 postsession 2), and there was not a significant change in those responses. However, students did demonstrate significant improvements in the other categories: They agreed more strongly that they could name the important elements of PCE (3.0 vs. 4.2 and 4.5), they reported feeling more confident in their ability to deliver PCE (3.4 vs. 3.9 and 4.1), and they increasingly agreed that they had had opportunities

Table 1. Pre- and Postsession Survey Results

Statement	Presession Mean ($N = 27$)[a]	Postsession 1 Mean ($N = 27$)[a]	*p*	Postsession 2 Mean ($N = 30$)[a]	*p*
Delivering patient-centered education (for diagnoses/medications) is an important skill for health care providers.	4.9	4.9	.646	4.9	.885
I can name the important elements of patient-centered education (for diagnoses/medications).	3.0	4.2	<.001[c]	4.5	<.001[c]
I am confident in my ability to deliver patient-centered education (for diagnoses/medications).	3.4	3.9	.002[c]	4.1	<.001[c]
I have had opportunities to practice delivering patient-centered education (for diagnoses/medications).	3.6	4.5	<.001[c]	4.6	<.001[c]
Role-plays are an effective tool for learning patient-centered education (for diagnoses/medications).[b]		4.3		4.4	

[a]Rated on a 5-point Likert scale (1 = *strongly disagree,* 2 *disagree,* 3 = *neutral,* 4 = *agree,* 5 = *strongly agree*).
[b]Statement not included on presession survey.
[c]Significant at $p < .05$.

Copyright © 2020 Mitchell and Goldenberg This is an open-access publication distributed under the terms of the Creative Commons Attribution license.

to practice delivering PCE (3.6 vs. 4.5 and 4.6). Finally, though this was not assessed in the presurvey, students felt after both sessions that role-plays were an effective tool for learning PCE (4.3 and 4.4).

Qualitative feedback from the free-response portion of the surveys was largely positive and is summarized thematically in Table 2. The main theme that emerged was the ability to have engaging, interactive, and realistic practice through role-play exercises. Areas for improvement, while few, mostly centered around logistical concerns and time constraints, such as a couple of students not feeling they had enough time to complete the exercise. A number of students also commented on their desire to have more faculty observe the role-play exercises so they could receive immediate feedback from those with more experience than their peers.

Discussion

This workshop filled an important gap in published curricular offerings as a comprehensive and interactive curriculum for improving medical students' abilities to provide PCE. Based on survey evaluation, students agreed that this was an important skill, and the interactive workshop increased their confidence to deliver and describe the key elements of PCE. They found the use of role-play exercises both helpful and enjoyable. Based on qualitative feedback, students were generally enthusiastic about the interactive and engaging nature of the workshop.

Table 2. Summary of Postsession Comments Grouped by Common Themes

Theme	Representative Comments
Strengths:	
Opportunity for skills practice	"Emphasis on practice and skill building."<br>"Role-playing with common meds is helpful."<br>"Saying the words out loud is harder than thinking them, so it is helpful to be forced to practice."<br>"Practice to translate scientific knowledge of medications into plain language."
Playing the role of patient	"Helpful to play the patient."<br>"Opportunity to role play from different points of view."<br>"Playing the patient was useful to imagine what it would be like."
Realistic and clear cases	"Concrete clinical scenarios."<br>"Clear role plays."<br>"Great cases that had realistic concerns."<br>"Working with complex but real scenarios."
Interaction with peers	"Great to exchange feedback with peers."<br>"Useful to learn from peers' approach."<br>"Working with our peers in a safe setting."
Areas for improvement:	
Timing and breakdown of session	"Lecture was too long."<br>"I didn't have time to go during role play."<br>"Allow for more time to practice."<br>"Time management."
Need for extra supervision/feedback	"Would like more feedback from instructors."<br>"Medical students evaluating medical students didn't work."<br>"Resident preceptors to provide feedback would be helpful."
Scenarios too detailed	"Patient scenarios too detailed."<br>"Explaining diagnosis and medication was too much."<br>"Narrowing the scope of information that needs to be communicated."

As facilitators, we had a largely positive experience developing and delivering this workshop. For the brief didactic portion of the workshop, students seemed to appreciate the rationale for teaching these skills and were highly engaged. For the interactive and discussion-based portion of the sessions, students were active participants and came up with meaningful responses. Finally, though logistically challenging at times, the role-play exercises produced some excellent interactions and allowed students to gain important insights both experientially and from their peers in a low-pressure environment. Moving forward, we plan to develop more streamlined practices for handing out materials, grouping students, separating into breakout rooms, and recalling students to the wrap-up portion of the session. The majority of constructive feedback and facilitator concerns stemmed from these logistical issues. We are also considering recruiting additional facilitators to observe the role-play exercises and offer real-time feedback.

While this workshop has been delivered at only one institution, in the primary care and psychiatry clerkship, with two groups of students, and by two facilitators, we do not anticipate significant barriers to its generalizability across other, similar settings. The interactive exercises do require some level of prerequisite medical knowledge for students to provide sufficient patient education, so one limitation is that this workshop may not fit well with students early in their preclinical training. Though students demonstrated significant improvements in their confidence to name and use PCE skills, we did not employ a skills-based assessment. Therefore, it is unclear whether these positive attitudinal changes will translate into meaningful improvements in students' ability to educate patients. Furthermore, it is not known how durable any improvement in student knowledge and confidence will be over time.

Moving forward, this workshop will continue to be delivered to additional groups of students at our institution. We believe it can be easily used at other institutions within specific clerkships or as part of an overall clinical skills curriculum. Potentially, this workshop can be modified for use in different clerkships

Copyright © 2020 Mitchell and Goldenberg This is an open-access publication distributed under the terms of the Creative Commons Attribution license.

(e.g., surgery, OB/GYN, etc.), though the content and role-play scenarios may need to be adapted with relevant diagnoses and interventions. The workshop would particularly lend itself to teaching a number of other important patient education scenarios in an interactive way, including informed consent for procedures, consent for blood transfusions, health and wellness counseling, and so on. The workshop could also be modified for use with residents, faculty, or other health care learners, given appropriate alterations in content.

Though this workshop was developed prior to the widespread use of virtual learning platforms during the coronavirus pandemic, we believe it could be adapted for teleconference classroom teaching with some minor alterations that would not meaningfully change the content or experience of the sessions. Most notably, the role-play portion of the workshop could be completed using the breakout room function available in many virtual learning platforms, so that learners could still engage with one another in small groups. The handouts explaining the role-play scenarios could be disseminated electronically, as could the pre- and postsurveys, which might improve the efficiency of data collection and analysis.

A further area of study would be to follow these students and ascertain whether implementation of this workshop translates into meaningful differences during interaction with patients over time. An intermediate step could include simulations with standardized patients, which would provide a more lifelike environment and allow for more objective feedback on performance.

An increased emphasis within medical school curricula on providing PCE and developing these valuable skills will benefit both patients and learners. This workshop is a model curriculum that is novel, comprehensive, interactive, engaging, and modifiable for multiple contexts.

Appendices

A. Facilitator Guide.docx
B. Presurvey.docx
C. Session 1 Patient Education Diagnoses.pptx
D. Video.mp4
E. Session 1 Role-Play Scenarios.docx
F. Session 1 Postsurvey.docx
G. Medication Research Worksheet.docx
H. Session 2 Patient Education Medications.pptx
I. Session 2 Role-Play Scenarios.docx
J. Session 2 Postsurvey.docx

All appendices are peer reviewed as integral parts of the Original Publication.

Thomas O. Mitchell, MD: Resident, Department of Psychiatry, Yale University School of Medicine; ORCID: https://orcid.org/0000-0003-3401-4235

Matthew N. Goldenberg, MD, MSc: Associate Professor, Department of Psychiatry, Yale University School of Medicine; ORCID: https://orcid.org/0000-0001-8859-6299

Acknowledgments
We wish to thank Dr. Walker Kernan and Dr. Michael Green for help with producing the educational video used in our materials.

Disclosures
None to report.

Funding/Support
None to report.

Ethical Approval
Reported as not applicable.

References

1. Mead N, Bower P. Patient-centredness: a conceptual framework and review of the empirical literature. *Soc Sci Med*. 2000;51(7): 1087-1110. https://doi.org/10.1016/S0277-9536(00)00098-8
2. Hoving C, Visser A, Mullen PD, van den Borne B. A history of patient education by health professionals in Europe and North America: from authority to shared decision making education. *Patient Educ Couns*. 2010;78(3):275-281. https://doi.org/10.1016/j.pec.2010.01.015
3. Constand MK, MacDermid JC, Dal Bello-Haas V, Law M. Scoping review of patient-centered care approaches in healthcare. *BMC Health Serv Res*. 2014;14:271. https://doi.org/10.1186/1472-6963-14-271
4. Siddharthan T, Rabin T, Canavan ME, et al. Implementation of patient-centered education for chronic-disease management in Uganda: an effectiveness study. *PloS One*. 2016;11(11): e0166411. https://doi.org/10.1371/journal.pone.0166411
5. Hersh L, Salzman B, Snyderman D. Health literacy in primary care practice. *Am Fam Physician*. 2015;92(2):118-124.
6. Shay LA, Lafata JE. Where is the evidence? A systematic review of shared decision making and patient outcomes. *Med Decis Making*. 2015;35(1):114-131. https://doi.org/10.1177/0272989X14551638

Copyright © 2020 Mitchell and Goldenberg This is an open-access publication distributed under the terms of the Creative Commons Attribution license.

7. Wilson SR, Strub P, Buist AS, et al; Better Outcomes of Asthma Treatment (BOAT) Study Group. Shared treatment decision making improves adherence and outcomes in poorly controlled asthma. *Am J Respir Crit Care Med*. 2010;181(6):566-577. https://doi.org/10.1164/rccm.200906-0907OC
8. Ong LML, de Haes JCJM, Hoos AM, Lammes FB. Doctor-patient communication: a review of the literature. *Soc Sci Med*. 1995; 40(7):903-918. https://doi.org/10.1016/0277-9536(94)00155-M
9. Benham-Hutchins M, Staggers N, Mackert M, Johnson AH, deBronkart D. “I want to know everything”: a qualitative study of perspectives from patients with chronic diseases on sharing health information during hospitalization. *BMC Health Serv Res*. 2017;17:529. https://doi.org/10.1186/s12913-017-2487-6
10. Dawes PJ, Davison P. Informed consent: what do patients want to know? *J R Soc Med*. 1994;87(3):149-152.
11. Hallett C, Gupta S, Priebe S. What do outpatients with schizophrenia and mood disorders want to learn about their illness? *Psychiatr Serv*. 2013;64(8):764-769. https://doi.org/10.1176/appi.ps.201200382
12. Tamirisa NP, Goodwin JS, Kandalam A, et al. Patient and physician views of shared decision making in cancer. *Health Expect*. 2017;20(6):1248-1253. https://doi.org/10.1111/hex.12564
13. Feng B, Bell RA, Jerant AF, Kravitz RL. What do doctors say when prescribing medications? An examination of medical recommendations from a communication perspective. *Health Commun*. 2011;26(3):286-296. https://doi.org/10.1080/10410236.2010.550020
14. Bittner A, Bittner J, Jonietz A, Dybowski C, Harendza S. Translating medical documents improves students’ communication skills in simulated physician-patient encounters. *BMC Med Educ*. 2016;16:72. https://doi.org/10.1186/s12909-016-0594-4
15. Tokuda Y, Okamoto S, Yoshioka Y, et al. The influence of medical jargon mixed with foreign terminology in the Japanese clinical environment. *Intern Med*. 2008;47(14):1329-1334. https://doi.org/10.2169/internalmedicine.47.0862
16. Alelwani SM, Ahmed YA. Medical training for communication of bad news: a literature review. *J Educ Health Promot*. 2014;3:51.
17. Boyle D, Dwinnell B, Platt F. Invite, listen, and summarize: a patient-centered communication technique. *Acad Med*. 2005;80(1):29-32. https://doi.org/10.1097/00001888-200501000-00008
18. Fortin AH, Haeseler FD, Angoff N, et al. Teaching pre-clinical medical students an integrated approach to medical interviewing: half-day workshops using actors. *J Gen Intern Med*. 2002;17(9): 704-708. https://doi.org/10.1046/j.1525-1497.2002.00628.x
19. Laidlaw TS, MacLeod H, Kaufman DM, Langille DB, Sargeant J. Implementing a communication skills programme in medical school: needs assessment and programme change. *Med Educ*. 2002;36(2):115-124. https://doi.org/10.1046/j.1365-2923.2002.01069.x
20. Vijn TW, Fluit CRMG, Kremer JAM, Beune T, Faber MJ, Wollersheim H. Involving medical students in providing patient education for real patients: a scoping review. *J Gen Intern Med*. 2017;32(9):1031-1043. https://doi.org/10.1007/s11606-017-4065-3
21. Mrduljaš Đujić N, Žitnik E, Pavelin L, et al. Writing letters to patients as an educational tool for medical students. *BMC Med Educ*. 2013;13:114. https://doi.org/10.1186/1472-6920-13-114
22. Saba GW, Chou CL, Satterfield J, et al. Teaching patient-centered communication skills: a telephone follow-up curriculum for medical students. *Med Educ Online*. 2014;19(1):22522. https://doi.org/10.3402/meo.v19.22522
23. Karpa K, Stollar K. Medication optimization and patient education in heart failure: a standardized patient case for clerkship students. *MedEdPORTAL*. 2016;12:10419. https://doi.org/10.15766/mep_2374-8265.10419
24. Nestel D, Tierney T. Role-play for medical students learning about communication: guidelines for maximising benefits. *BMC Med Educ*. 2007;7:3. https://doi.org/10.1186/1472-6920-7-3
25. Brega AG, Barnard J, Mabachi NM, et al. *AHRQ Health Literacy Universal Precautions Toolkit*. 2nd ed. Agency for Healthcare Research and Quality; 2015. AHRQ publication 15-0023-EF. https://www.ahrq.gov/health-literacy/quality-resources/tools/literacy-toolkit/healthlittoolkit2.html
26. Welcome to the Always Use Teach-back! training toolkit. Always Use Teach-back! Accessed July 31, 2020. http://www.teachbacktraining.org/home

Received: April 29, 2020
Accepted: August 21, 2020
Published: December 10, 2020

Copyright © 2020 Mitchell and Goldenberg This is an open-access publication distributed under the terms of the Creative Commons Attribution license.
